# Conformational Dependence of Triplet Energies in Rotationally Hindered N‐ and S‐Heterocyclic Dimers: New Design and Measurement Rules for High Triplet Energy OLED Host Materials

**DOI:** 10.1002/chem.202100036

**Published:** 2021-03-10

**Authors:** Iain A. Wright, Andrew Danos, Stephanie Montanaro, Andrei S. Batsanov, Andrew P. Monkman, Martin R. Bryce

**Affiliations:** ^1^ Department of Chemistry Durham University South Road Durham DH1 3LE UK; ^2^ Department of Chemistry Loughborough University Loughborough Leicestershire LE11 3TU UK; ^3^ Department of Physics Durham University South Road Durham DH1 3LE UK

**Keywords:** dihedral angle, heterocycles, OLED, photophysics, triplet

## Abstract

A series of four heterocyclic dimers has been synthesized, with twisted geometries imposed across the central linking bond by *ortho*‐alkoxy chains. These include two isomeric bicarbazoles, a bis(dibenzothiophene‐*S*,*S*‐dioxide) and a bis(thioxanthene‐*S*,*S*‐dioxide). Spectroscopic and electrochemical methods, supported by density functional theory, have given detailed insights into how *para*‐ vs. *meta*‐ vs. broken conjugation, and electron‐rich vs. electron‐poor heterocycles impact the HOMO–LUMO gap and singlet and triplet energies. Crucially for applications as OLED hosts, the triplet energy (*E*_T_) of these molecules was found to vary significantly between dilute polymer films and neat films, related to conformational demands of the molecules in the solid state. One of the bicarbazole species shows a variation in *E*_T_ of 0.24 eV in the different media—sufficiently large to “make‐or‐break” an OLED device—with similar discrepancies found between neat films and frozen solution measurements of other previously reported OLED hosts. From consolidated optical and optoelectronic investigations of different host/dopant combinations, we identify that only the lower *E*_T_ values measured in neat films give a reliable indicator of host/guest compatibility. This work also provides new molecular design rules for obtaining very high *E*_T_ materials and controlling their HOMO and LUMO energies.

## Graphical abstract

**A new series of high** ***E***_**T**_ heterocyclic dimers has been synthesized with large dihedral angles enforced by steric hindrance. A *meta*‐conjugated carbazole dimer displays the highest *E*_T_ of 3.07 eV in dilute polymer film, however in a neat film this falls to 2.83 eV. Similar variation is observed across the series. This work highlights that to reliably estimate host/guest compatibility, *E*_T_ determined from phosphorescence of neat films must be reported for new host materials. The currently accepted practice of reporting values from frozen solution or polymer films is no substitute.

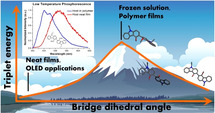

## Introduction

Carbazole is an important heterocyclic motif in organic electronics. The lone pair of the amine nitrogen participates in the delocalized π‐electron cloud, making carbazole notably electron rich. This gives carbazole and its derivatives optical and electronic properties well suited for use in emissive devices based upon organic molecules. Carbazole has great synthetic versatility permitting systematic variations through substitution, interconversion and coupling reactions, allowing optimisation of the optoelectronic properties of carbazole derivatives for many applications.[^1^, ^2^, ^3^, ^4^, ^5^, ^6^, ^7^, ^8^, ^9^, ^10^, ^11^] Among the most important of these applications are their use as hole transport materials (HTMs),^[12]^ or as host materials in the emissive layer (EML) of organic light emitting diodes (OLEDs).[^13^, ^14^, ^15^, ^16^, ^17^] In OLEDs the host material is doped at low levels with an emissive molecule such as an organometallic phosphor (PhOLED) or a thermally activated delayed fluorescent (TADF) molecule. The host material assists with charge transport in the EML while also preventing aggregation or concentration‐induced quenching of the emitter.

During OLED operation excitons in the EML are formed in a triplet:singlet ratio of 3:1. PhOLEDs and TADF‐OLEDs take advantage of these typically non‐emissive triplet states, achieving high efficiencies by harvesting them for emission.[^18^, ^19^, ^20^, ^21^] PhOLEDs convert singlets into triplets by heavy‐atom‐enhanced spin‐orbit‐allowed intersystem crossing before emitting through phosphorescence. TADF‐OLEDs instead convert triplets into singlets by reverse intersystem crossing (rISC), which can then decay by (delayed) fluorescence.^[22]^ To facilitate either of these emission mechanisms several energetic considerations must be made to ensure host/dopant compatibility. Firstly, the frontier highest occupied and lowest unoccupied molecular orbitals (HOMO and LUMO, respectively) must be favorably positioned with respect to the electron and hole transport layers to ensure efficient charge injection. Additionally, the triplet energy (*E*_T_) of the host must be larger than that of the guest, so that triplet excitons are confined on the triplet harvesting emitter instead of being quenched via the host.[^16^, ^23^] Understanding how to control these factors remains an important challenge in the design of molecular optoelectronic materials.

**Scheme 1:**
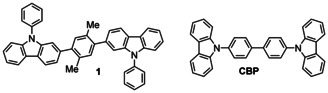
Molecular structures of twisted bicarbazole **1** and **CBP**.

Toward establishing rules for maximizing *E*_T_ and developing new host materials appropriate for high *E*_T_, deep‐blue emitters, we previously reported a large family of conformationally‐restricted π‐bridged bicarbazoles. In each, the two carbazole moieties were separated by 1,4‐phenylene π‐bridges and bound either *meta*‐ or *para*‐ to the nitrogen atom of the carbazole.^[24]^ Sterically demanding substituents were attached in the 2‐ and 5‐positions of the phenylene bridge which imposed a twist in the molecules. This structural modification disrupted conjugation and thereby increased *E*_T_. The positioning of the π‐bridge and the nature of its substituents had important consequences on the optoelectronic properties and led to host materials such as 1,4‐bis(9‐phenyl‐2‐carbazolyl)‐2,5‐dimethylbenzene **1** (Scheme 1) which outperformed the archetypical host material 4,4’‐bis(9‐carbazolyl)‐1,1’‐biphenyl (**CBP**) in OLED devices using the organometallic iridium phosphor FIrpic.

Despite this and other advances reported in recent years, carbazole‐containing EML hosts generally possess *E*_T_ too low for deep‐blue TADF emitters (*E*_T_<3 eV).^[25]^ However, we were motivated by the fact that *E*_T_ increases for the non‐planar host materials 4,4’‐bis(9‐carbazolyl)‐2,2’‐dimethylbiphenyl **CDBP** and 1,3‐bis(9‐carbazolyl)benzene **mCP** as conjugation length is decreased compared to planar **CBP** (structures of these and other host materials mentioned throughout this work are shown in Figure S1).[^26^, ^27^] Applying this approach to the previously reported bicarbazole series,^[24]^ removing the central 1,4‐phenylene π‐bridge and instead placing the alkoxy substituents directly onto adjacent carbazole rings of a simple carbazole‐carbazole dimer was anticipated to impose a larger dihedral angle between the carbazoles through steric clash. This modification would cause the conjugation length of the molecule to decrease further, leading to a desirable blue‐shift in optical properties and increased *E*_T_. We were also keen to explore and understand the direct consequences on the optoelectronic and charge transport properties of using different heteroatoms in place of nitrogen. Similarly, comparing different bond connectivity (*meta*‐ versus *para*‐) has previously revealed subtle yet significant differences in TADF compounds due to weaker electronic coupling across *meta*‐bridges[^28^, ^29^] with similar effects expected in the envisioned host materials.

**Scheme 2:**
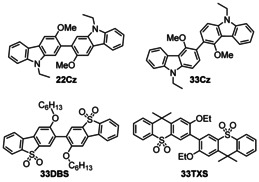
Molecular structures of the nitrogen (**22Cz** and **33Cz**) and sulfone (**33DBS** and **33TXS**) containing heterocyclic dimers. The numbering of the dimers denotes the positions of connectivity between the heterocycles according to IUPAC rules. Despite differences in IUPAC numbering, **22Cz** and **33DBS** both have linearly conjugated *p*‐quaterphenyl backbones.

Toward these goals of maximizing HTM and EML host suitability for deep‐blue emission in TADF‐OLEDs, we devised the twisted 2,2’‐bicarbazole **22Cz** alongside three analogs (Scheme 2): i) the *meta*‐conjugated 3,3’‐bicarbazole isomer **33Cz**, ii) the 3,3’‐bis(dibenzothiophene‐*S*,*S*‐dioxide) **33DBS** featuring an electron‐withdrawing sulfonyl group in place of the electron‐donating nitrogen atom, and iii) the 3,3’‐bis(thioxanthene‐*S*,*S*‐dioxide) **33TXS** which also features an electron withdrawing sulfone group. **33TXS** also features a clear break in conjugation due to the sp^3^ carbon atoms in the central 6‐membered ring of the thioxanthene—effectively decoupling the terminal phenyl rings from the central biphenyl moiety. It becomes apparent that there are several ways to segment these molecules when studying the photophysical implications of their molecular structures. For example, **22Cz** may be considered as a bis(biphenyl) derivative, a ring‐fused 2,2’‐dialkoxybiphenyl, or simply a twisted *p*‐quaterphenyl. The power of such an approach was well evidenced in a recent report by Fries et al, which revealed that the origins of room temperature phosphorescence (RTP) in tetra‐*N*‐phenylbenzidine were a consequence of its biphenyl core.^[30]^

The concise collection of compounds in Scheme 2 has allowed us to explore a wide range of design features including: the role of dihedral angles between the heterocycles, *meta*‐ versus *para*‐conjugation, electron donating versus electron withdrawing heteroatoms, and the effects of breaking or extending conjugation length on the HOMO–LUMO gaps (*E_g_*) and the singlet (*E*_S_) and triplet energies. We present the optical and electrochemical properties of these molecules and an assessment of their suitability as high *E*_T_ host materials in OLEDs. Additional information about their structural properties was obtained from single‐crystal X‐ray diffraction data. These experimental results are complemented by computational insights.

## Results and Discussion

### Synthesis

Significantly different routes were required to synthesize the isomeric bicarbazoles **22Cz** and **33Cz**.

**Scheme 3:**
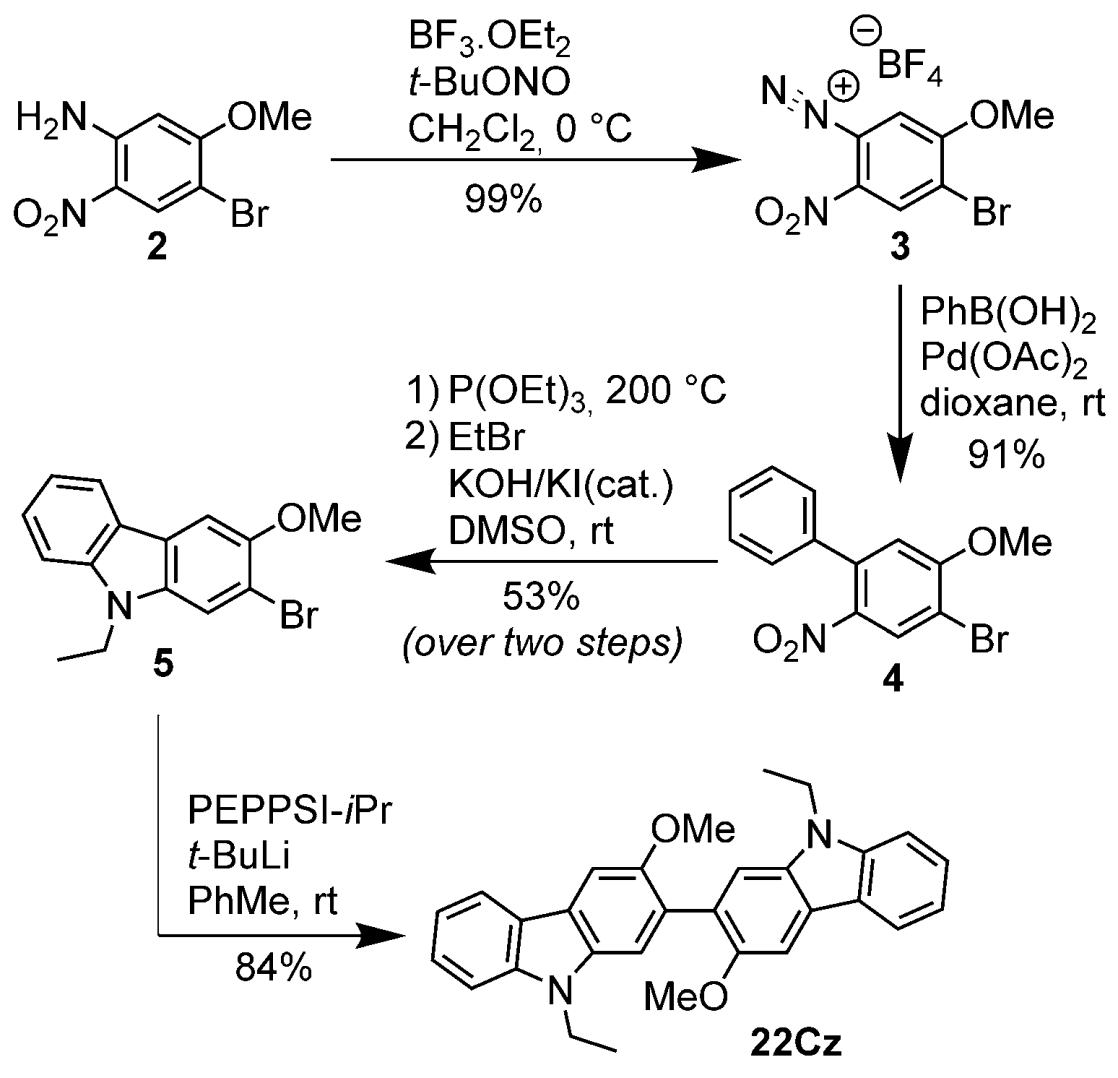
Synthesis of 2,2’‐bicarbazole **22Cz**.

**22Cz** was synthesized by the *tert*‐butyllithium‐mediated palladium‐catalyzed homocoupling of 2‐bromo‐9‐ethyl‐3‐methoxy‐9*H*‐carbazole **5** (Scheme 3).^[31]^ An alternative synthesis of **5** was previously reported by Kauffman et al via a rather arduous route beginning from *m*‐anisidine.^[32]^ We substantially simplified the route to **5** by starting from 4‐bromo‐5‐methoxy‐2‐nitroaniline **2**, which can be synthesized in large quantities from readily‐available 5‐chloro‐2‐nitroaniline according to the procedure of Sheibani and Wärnmark.^[33]^ **2** was converted to diazonium tetrafluoroborate salt **3** in 99 % yield and subsequently treated with phenylboronic acid in the presence of catalytic Pd(OAc)_2_ under air^[34]^ to give 2‐nitrobiphenyl derivative **4** in excellent yield. Subsequent Cadogan cyclization of **4** using P(OEt)_3_ at 200 °C for 20 minutes under microwave irradiation^[35]^ was followed immediately by alkylation with ethyl bromide to give **5**. Traditional convection heating gave comparable yields but requires significantly longer reaction times. The final PEPPSI‐*i*Pr/*t*BuLi mediated homocoupling of **5** yielded the desired product **22Cz** in 84 % yield.

**Scheme 4:**
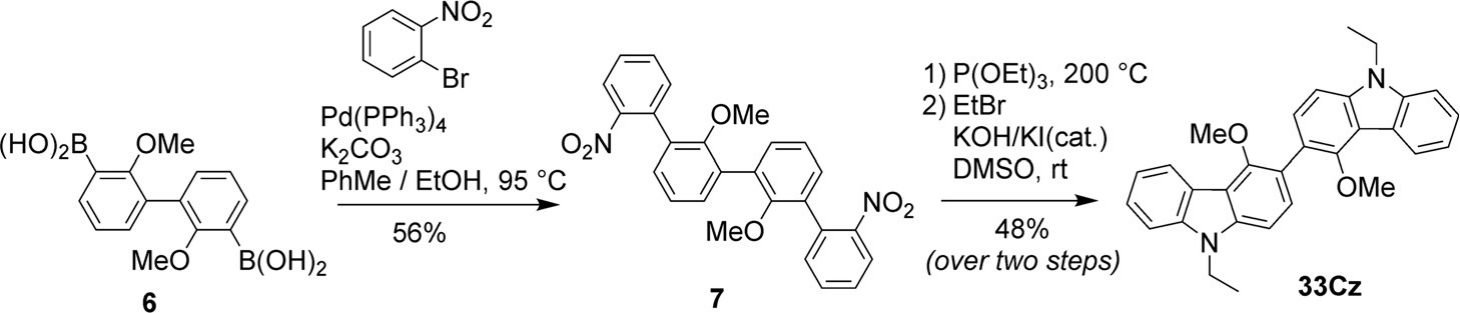
Synthesis of 3,3’‐bicarbazole **33Cz**.

The synthesis of **33Cz** (Scheme 4) began from the di(boronic acid) **6** which was easily synthesized in multi‐gram quantities according to the procedure of Kayal, Ducruet and Lee.^[36]^ Compound **6** was reacted with 1‐bromo‐2‐nitrobenzene under Suzuki coupling conditions to produce quaterphenyl **7** in 56 % yield. Subsequent two‐fold, two‐step cyclization/alkylation of **7**, as described for the synthesis of **22Cz**, yielded the desired product **33Cz** in 48 % yield over the final two steps.

**Scheme 5:**
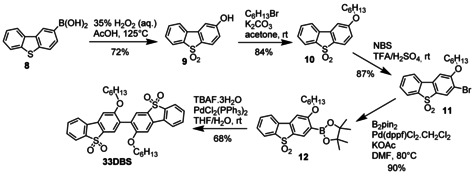
Synthesis of 3,3’‐bis(dibenzothiophene‐*S*,*S*‐dioxide) **33DBS**.

To synthesize **33DBS** (Scheme 5), in a modification of our previously reported procedure,^[37]^ dibenzothiophene‐2‐boronic acid **8** was oxidized to the sulfone and saponified in situ to produce the alcohol **9**. This was then deprotonated with K_2_CO_3_ and alkylated with *n*‐hexyl bromide under mild conditions to give 2‐hexyloxydibenzothiophene‐*S*,*S*‐dioxide **10**, the molecular structure of which was obtained using X‐ray crystallography (Figure S2). The longer hexyloxy chain was chosen to improve the solubility of the final compound **33DBS**. Bromination of **10** with NBS in a mixture of trifluoroacetic acid and sulfuric acid gave 3‐bromo‐2‐hexyloxydibenzothiophene‐*S*,*S*‐dioxide **11** selectively.^[38]^ The *tert*‐butyllithium‐mediated homocoupling approach employed in the final step toward **22Cz** failed when trying to dimerize **11** (and the similarly electron‐poor 3‐bromo‐2‐ethoxy‐9,9‐dimethyl‐9*H*‐thioxanthene‐*S*,*S*‐dioxide **16** vide infra) which may indicate that this methodology is less well suited to electron‐poor halides. This is substantiated somewhat in the original publication, as relatively few electron‐poor halides were employed and those that were relied upon the use of the PEPPSI‐*i*Pent catalyst.^[31]^ These reactions also displayed a notable drop in yield when the withdrawing group was *meta* to the halide rather than *ortho* or *para*, and therefore unable to stabilize the metallated intermediate. Instead, Miyaura borylation of **11** furnished boronate ester **12** in excellent yield, which was then homocoupled under mild conditions using PdCl_2_(PPh_3_)_2_ in the presence of fluoride,^[39]^ to produce the target dimer **33DBS** in 68 % yield.

**Scheme 6:**
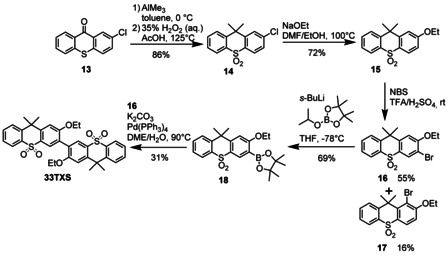
Synthesis of 3,3’‐bis(thioxanthene‐*S*,*S*‐dioxide) **33TXS**.

Finally, toward **33TXS** (Scheme 6), commercially available and inexpensive 2‐chlorothioxanthone **13** was treated with trimethylaluminium in anhydrous toluene, and the crude 9,9‐dimethyl‐9*H*‐thioxanthene intermediate was immediately oxidized with aqueous hydrogen peroxide in AcOH to provide **14** in 86 % yield over two steps. The generally higher solubility of the bent thioxanthone heterocycle compared to planar dibenzothiophenes allowed for the use of shorter solubilising alkoxy substituents compared to **33DBS**, therefore nucleophilic aromatic substitution with NaOEt in a mixture of DMF and EtOH produced **15**, followed by bromination to **16** using the same conditions as for dibenzothiophene‐*S*,*S*‐dioxide **11**. Compared to the synthesis of **11** this reaction was found to be less regioselective as significant quantities of the highly congested 1‐bromo isomer **17** were isolated after purification by column chromatography. Also, similarly to **11**, it was not possible to dimerize **16** directly. At this point significant differences in the reactivity of dibenzothiophene‐*S*,*S*‐dioxide and thioxanthene‐*S*,*S*‐dioxide became apparent. Attempts to produce **18** in an analogous fashion to **12** by using Miyaura borylation of **16** failed, therefore lithium‐halogen exchange followed by trapping with 2‐*iso‐*propoxy‐4,4,5,5‐tetramethyl‐1,3,2‐borolane was employed successfully. The fluoride mediated homocoupling reaction applied in the dimerization of **12** was also not successful in producing **33TXS** from **18**. In this instance, straightforward Suzuki coupling of **18** with previously isolated **16** gave the desired dimer **33TXS** in a modest yield after purification.

### Structural properties

**Figure 1:**
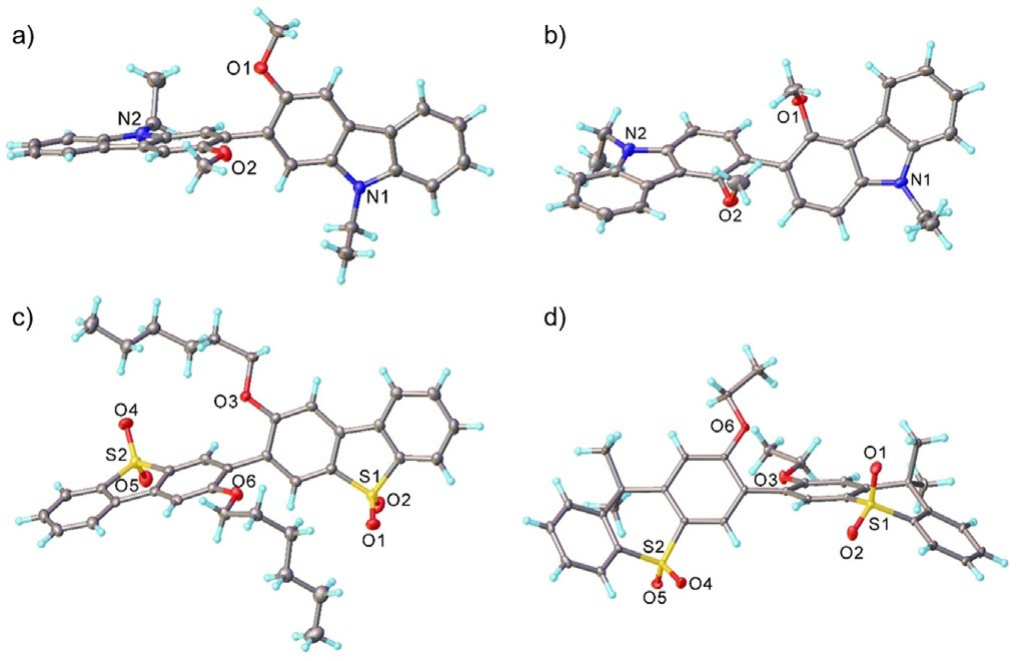
Molecular structures of a) **22Cz**, b) **33Cz**, c) **33DBS** and d) **33TXS**. Atomic displacement ellipsoids are drawn at the 50 % probability level.

Molecular structures determined by X‐ray crystallography (Figure 1) reveal that the planar carbazole moieties in **22Cz** are nearly perpendicular at 81.3°. This is much more twisted than the 25° dihedral angle observed for the parent dimer 9,9’‐diethyl‐2,2’‐bicarbazole,^[7]^ while in **33Cz** the carbazole planes are twisted by 57.5° about the connecting C−C bond (with methoxy groups in *anti*‐positions). The crystal structure of **33DBS** contains two independent molecules of broadly similar conformations, twisted about the central C−C bonds by 70.2° and 50.6°, with hexyloxy groups in *anti*‐positions. Each dibenzothiophene moiety shows a slight bend, measured by the dihedral angle between the benzene rings’ planes, viz. 2.8° and 10.1° in the first molecule, 5.8° and 11.4° in the second. In **33TXS**, thioxanthene moieties are folded along their S…CMe_2_ vectors by 32.5° and 33.7°; the molecule is twisted by 59.0° about the central C−C bond, with the two ethoxy groups in *syn*‐positions. It is noteworthy that the crystal packing of all these molecules display no π–π stacking interactions (Figures S3–S6). This is an important observation since we have previously shown the strong propensity for carbazole‐containing hosts and emitters to form intermolecular interactions which adversely affect *E*_T_.[^40^, ^41^, ^42^] Crystal data and experimental details are included in the Supporting Information (Table S1).

### Solution Properties

**Figure 2:**
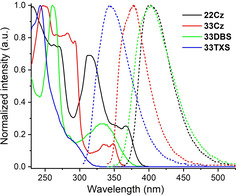
Absorption (solid lines) and emission spectra (dashed lines) of **22Cz**, **33Cz**, **33DBS** (*λ*_Exc_=330 nm) and **33TXS** (*λ*_Exc_=290 nm) obtained in CH_2_Cl_2_ solution.

UV/Vis absorption and fluorescence spectra were obtained in CH_2_Cl_2_ solution (Figure 2 and Table 1).

**Table 1:** Absorption and emission data for the dimers obtained in CH_2_Cl_2_ solution.

	*λ*_Abs_ [nm]	*λ*_Abs(onset)_ [nm]	*E*_g_^opt^ [eV]^[a]^	*λ*_max(PL)_ [nm]^[b]^	*E*_S1_ [eV]^[c]^
**22Cz**	232, 243(sh), 263, 270, 314, 367	395	3.14	401	3.37
**33Cz**	250, 283, 293, 335, 349	367	3.38	378	3.61
**33DBS**	281, 264, 332	387	3.21	403	3.48
**33TXS**	242, 285	330	3.76	343	4.02

[a] Optical HOMO–LUMO gaps (*E*_g_^opt^) calculated using the onset of the longest wavelength absorbance band. [b] For **22Cz**, **33Cz** and **33DBS** *λ*_Exc_=330 nm. For **33TXS** *λ*_Exc_=290 nm. [c] Singlet energies (*E*_S1_) calculated using the onset of the fluorescence band.

The absorption spectra are all dominated by intense π–π* transitions below 300 nm. The overall band shape of the carbazole dimers **22Cz** and **33Cz** is in good agreement with those of simple 9,9’‐diethyl‐2,2’‐ and 9,9’‐diethyl‐3,3’‐bicarbazole as published by Kato and co‐workers,^[7]^ albeit with some additional fine structure arising from transitions over the 9‐ethylcarbazole rings at longer wavelengths.^[43]^ The longest wavelength bands of both **33DBS** and **33TXS** (a shoulder in the case of **33TXS**) display a gaussian waveform with less fine structure than those of the carbazole dimers. In the case of **33DBS**, this is not representative of features in the UV/Vis spectra of the parent undecorated dibenzothiophene‐*S*,*S*‐dioxide heterocycle.^[44]^ This difference can be explained by the fact that the strongly electron‐donating alkoxy group is *para* to the electron‐withdrawing sulfone, therefore this feature is assigned to push‐pull effects between these groups. Across the series the energy of the onset of absorbance, corresponding to the optical HOMO–LUMO gap (*E*_g_^opt^), is dictated by the effective conjugation length of each molecule. Comparing the *para*‐conjugated **22Cz** with *meta*‐conjugated **33Cz**, the *E*_g_^opt^ increases from 3.14 eV to 3.38 eV accordingly. The *p*‐quaterphenyl backbones cause **22Cz** and **33DBS** to display the lowest energy absorbance features, and rather similar *E*_g_^opt^ values. In contrast, **33TXS** which has the smallest effective conjugation length, displays the largest *E*_g_^opt^.

The fluorescence spectra for all the compounds have very limited fine structure. A shoulder observed for sterically unhindered 2,2’‐ and 3,3’‐bicarbazole is retained for **33Cz**, but is absent for **22Cz**.^[7]^

UV/Vis spectroscopy reveals an interesting observation about the linearly conjugated dimers **22Cz** and **33DBS**; changing the heteroatom from an electron‐donating nitrogen in **22Cz** to an electron‐withdrawing sulfone in **33DBS** results in minimal change in the *E_g_* ^opt^.

**Figure 3:**
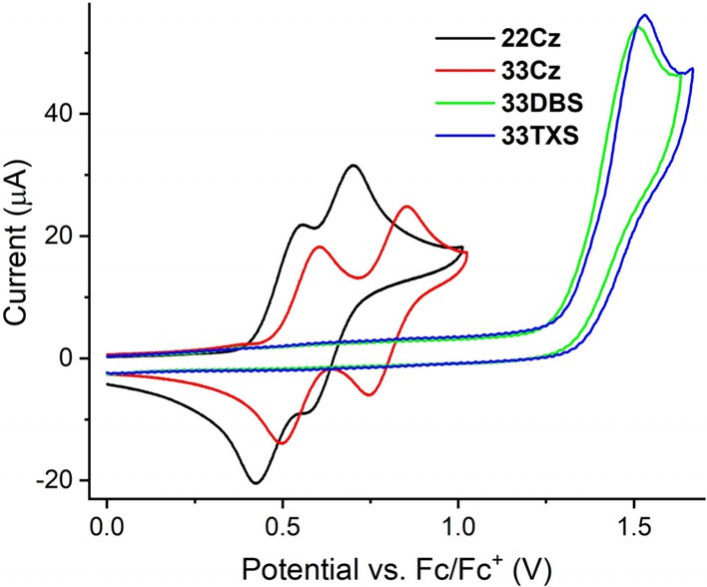
Cyclic voltammetry of **22Cz**, **33Cz**, **33DBS** and **33TXS**.

While UV/Vis and other optical measurements provide energy gaps, this does not provide any insight into the relative energies of the orbitals concerned. The energetic positioning of the frontier orbitals is nonetheless essential to make an informed selection of HTM to ensure good compatibility between other transport layers in devices. Cyclic voltammetry was therefore performed (in CH_2_Cl_2_ with tetra‐*n‐*butylammonium hexafluorophosphate as supporting electrolyte) to obtain insights into the position of the frontier orbitals. The results are presented in Figure 3 and Table 2. Carbazole dimers are known to be readily oxidized at low potentials.[^7^, ^17^, ^24^] Consistent with this, both **22Cz** and **33Cz** displayed two sequential and reversible single‐electron oxidations corresponding to radical cation formation on each of the carbazole rings. The peak separation between the anodic and cathodic peak potentials was slightly higher than the theoretical 59 mV (≈100 mV measured) but the anodic and cathodic peak currents were comparable. Compared to the parent dimers the electrochemical stability and reversibility of **22Cz** and **33Cz** are greatly improved by the presence of the electron rich *o*,*o*’‐dimethoxybiphenyl core.^[7]^ This inhibits anodic dimerization and polymerization pathways by localizing any radical cation or dicationic states to the core of the molecule.^[46]^ The diminished electronic communication between the adjacent carbazoles, moving from the *para*‐conjugated **22Cz** to the *meta*‐conjugated **33Cz**, is evident from the increased spacing between the oxidation events for **33Cz**. The longer effective conjugation length of **22Cz** results in it having the lowest oxidation potential. The onset of the first oxidation wave of each molecule was used to estimate the HOMO energy at −5.41 eV for **22Cz** and −5.56 eV for **33Cz**. Both **33DBS** and **33TXS** display a single irreversible oxidation at near‐identical potentials, with an onset approximately +0.90 V higher than that of the carbazole dimers. This gives **33DBS** and **33TXS** HOMO energies of −6.32 eV and −6.34 eV, respectively.

**Table 2:** Electrochemical data of the heterocyclic dimers.

	*E*_Ox1_ [V]	*E*_Ox2_ [V]	*E*_Ox(onset)_ [V]	HOMO [eV]^[a]^	LUMO [eV]
**22Cz**	+0.49	+0.63	+0.31	−5.41	−2.27
**33Cz**	+0.55	+0.80	+0.46	−5.56	−2.18
**33DBS**	+1.51		+1.22	−6.32	−3.11
**33TXS**	+1.53		+1.24	−6.34	−2.58

[a] HOMO energies calculated from the onset of the first oxidation wave using the HOMO of Fc/Fc+ as a reference point (−5.10 eV).^[45]^ [b] LUMO values were estimated using HOMO+*E*_g_^opt^.

The results for **22Cz** and **33DBS** are perhaps the most striking. Their *E*_g_^opt^ values are nearly identical (Δ*E*_g_^opt^ 0.07 eV; Table 1) but their oxidation potentials are 0.90 V apart. This reveals that while the energy difference between the HOMO and LUMO is very similar, the frontier orbitals for these two molecules are offset by 0.90 V solely due to the change in heteroatom. The comparable *E*_g_^opt^ implies that the HOMO and LUMO are distributed primarily over the conjugated *p*‐quaterphenyl backbone (with minimal density on the heteroatom, as confirmed by DFT, vide infra). This implies that the HOMO and LUMO energies can be tuned independently of the magnitude of the HOMO–LUMO gap simply by changing the heteroatom. This provides a valuable tool for the fine‐tuning of redox behavior independently of optical properties.

### DFT and frontier molecular orbital study

**Figure 4:**
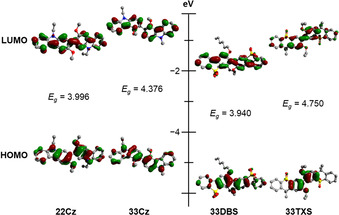
Molecular orbital plots and relative energies for the frontier orbitals of **22Cz**, **33Cz**, **33DBS**, and **33TXS** obtained from DFT (B3LYP/6‐31G**). For each molecule the HOMO is the lower and LUMO is the upper diagram.

Density functional theory (DFT) and time‐dependent DFT (TDDFT) provided further insights into the impact of structural changes on frontier orbital energies and distributions, as well as their effects on the singlet and triplet energies. Calculations were performed with ORCA v4.0.1.2[^47^, ^48^] using the B3LYP hybrid functional and 6‐31G** and def2‐SVP basis sets.[^49^, ^50^, ^51^, ^52^, ^53^] Ground state structural optimizations were performed prior to frontier orbital calculations (Table 3). Molecular orbital plots and the relative energies of the HOMO and LUMO of each dimer are shown in Figure 4.

**Table 3:** Orbital energies, energy gaps and dihedral angle of the central C−C bond obtained from DFT (B3LYP/6‐31G**).

B3LPY 6‐31G**	HOMO‐1 [eV]	HOMO [eV]	LUMO [eV]	LUMO+1 [eV]	*E*_g_^calc^ [eV]	*E*_T1_ [eV]	*E*_S1_ [eV]	Δ*E*_ST_ [eV]	*τ* [°]
**22Cz**	−4.843	−4.777	−0.781	−0.235	3.996	2.879	3.545	0.666	53.4/9
**33Cz**	−5.256	−4.824	−0.448	−0.424	4.376	3.274	3.829	0.555	47.3/4
**33DBS**	−6.422	−5.848	−1.908	−1.440	3.940	2.751	3.535	0.784	50.9
**33TXS**	−6.290	−5.849	−1.099	−0.953	4.750	3.344	4.177	0.833	47.4

In agreement with the crystallographically determined molecular structures, the DFT results predict that the structures of the molecules with linear connectivity **22Cz** (*τ*=53.4°) and **33DBS** (*τ*=50.9°) are more twisted across the central C−C bond than **33Cz** and **33TXS** (both *τ*≈47°). These values are also more twisted (by ≈15° for **22Cz** ^[7]^ and ≈10° for **33Cz** ^[54]^) than those reported for analogous non‐functionalized bicarbazoles calculated at similar levels of theory. While differences between **33DBS** and **33TXS** can be explained by the greater steric hindrance of the larger hexyloxy groups in **33DBS**, the wider dihedral angle in **22Cz** compared to **33Cz** occurs despite the longer conjugation length of the *p*‐quaterphenyl backbone of **22Cz**, which would typically be expected to have a greater planarizing effect on the molecule. This observation can be explained by the electron‐donating influence of the lone pair of the carbazole nitrogen. The effect is strongest at the 3‐position of the carbazole ring (*para* to the nitrogen), which is also the position of the central C−C bond in **33Cz**. This electronic effect thereby planarizes this “benzidine‐like” biphenyl region of **33Cz** by increasing the energetic penalty of disrupting conjugation across this region through twisting.

When comparing the DFT calculated structures with those obtained from crystallography the trend in variation of the dihedral angle from one molecule to another agreed, however the actual magnitudes did not. The X‐ray structures proved more twisted in almost all cases. This is attributed to intermolecular packing interactions within the crystal structure which can result in significant differences between predicted and experimental geometries of organic molecules.[^55^, ^56^] Further analysis of the crystal packing effects are included in the Supplementary Information.

The general trends in the DFT energy calculations agree well with our spectroscopic and electrochemical observations. In particular, the data highlight the stepwise increase in LUMO and *E_g_* when moving from the linearly conjugated *para*‐quaterphenyl systems of **22Cz** and **33DBS** to the *meta*‐conjugated **33Cz** and then the broken conjugation of **33TXS**.

The HOMO of all four molecules resides primarily over the central benzene rings of the *o*,*o’*‐dialkoxybiphenyl core. This is in contrast with the results of calculations on simple non‐hindered dimers where the HOMO is evenly distributed across the conjugated backbone.[^7^, ^44^] Due to their longer linear conjugation, the LUMO of both **22Cz** and **33DBS** extends in a quinoidal fashion across the full length of the *para*‐quaterphenyl backbone of each molecule. In the *meta*‐conjugated **33Cz** the LUMO (and LUMO+1, shown in Figure S7) is evenly distributed across both carbazoles with no contribution across the linking C−C bond. The LUMO of **33TXS** can only extend across the central biphenyl moiety with minor contributions from the terminal phenyl rings. **33Cz** is unique in this present series, with the *meta*‐conjugated dimer displaying some degeneracy between the LUMO and LUMO+1. Interestingly, our previous observation of significant degeneracy in the HOMO manifold of *para*‐conjugated 1,4‐phenylene bridged bicarbazoles, and in the LUMO manifold of *meta*‐conjugated 1,4‐phenylene π‐bridged bicarbazoles,^[24]^ is not reproduced as a general trend in these non‐π‐bridged dimers.

While the extent of conjugation between adjacent heterocycles dictates the spatial arrangement of the frontier orbitals, DFT indicates that the nature of the central *o*,*o’*‐dialkoxybiphenyl moiety controls their overall energy profile. Comparing the influence of the electron‐donating N atom to the electron‐withdrawing SO_2_ group in **22Cz** and **33DBS**, the *E*_g_ shows minimal change. Both the HOMO and LUMO are stabilized by essentially equal amounts (ca. 1 eV) by the SO_2_ group. Both **22Cz** and **33DBS** are consequently blue emitters with comparable *E*_g_ but with frontier orbitals of very different energies—effectively changing the energy space within which these materials can operate as HTMs and consolidating the observations made spectroscopically and electrochemically. **33DBS** and **33TXS** are predicted to have nearly identical HOMO energies, as was observed in the cyclic voltammetry, but the truly broken conjugation of **33TXS** results in reduced stabilization of the LUMO compared to **33DBS**, as was also found experimentally.

TDDFT was used to calculate the positions of the first 15 singlet and triplet states (Tables S5–S8) for each molecule. Linear conjugation in **22Cz** and **33DBS** gives rise to a larger difference between the first excited singlet and triplet energies (Δ*E*_ST_) compared to the *meta*‐conjugated **33Cz** and **33TXS**. From TDDFT, the *meta*‐linked **33Cz** and **33TXS** displayed the highest triplet energies overall, consistent with smaller electronic coupling across the *meta*‐linkage.[^26^, ^28^]

### Solid state photophysics

**Figure 5:**
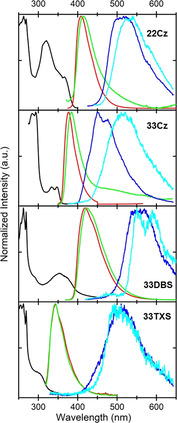
Black: absorbance in Zeonex, red: emission in Zeonex, dark blue: phosphorescence in Zeonex, green: neat film emission, sky‐blue: neat film phosphorescence.

The absorption and emission characteristics of the four dimers were initially measured as films of 0.5 wt/wt % in the cycloolefin polymer host Zeonex (Figure 5 and Table 4).

**Table 4:** Optical properties of solid‐state films.

	**Zeonex**					**Neat**		
	*λ*_Abs_ [nm]	*λ*_PL Onset, Peak_ [nm]	*E*_S1_ [eV]^[a]^	*λ*_Phos Onset, Peak_ [nm]	*E*_T1_ [eV]^[b]^	*λ*_PL Peak_ [nm]	*λ*_Phos Onset, Peak_ [nm]	*E*_T1_ [eV]^[b]^
**22Cz**	313, 320, 354, 366	385, 409	3.22	460, 514	2.70	415	468, 531	2.65
**33Cz**	284, 293, 334, 347	355, 377	3.49	404, 451, 473	3.07	385	438, 514	2.83
**33DBS**	290, 354, 371	382, 418	3.24	498, 557	2.49	423	521, 550, 591	2.38
**33TXS**	300	315, 343	3.94	417, 498	2.97	343	450, 509	2.76

[a] Singlet energy (*E*_S1_) calculated using the onset of the fluorescence band. [b] Triplet energy (*E*_T1_) calculated using the onset of the phosphorescence band.

The overall profiles and trends of the solid‐state absorption and fluorescence spectra in Zeonex are very similar to those obtained in CH_2_Cl_2_ solution for all four compounds, although the longest wavelength absorbance was slightly red‐shifted in Zeonex for **33DBS** (39 nm) and **33TXS** (15 nm). Fluorescence spectra also displayed slight red‐shifts across the series, with the smallest effect for **33TXS**. Phosphorescence was collected at 77 K under a stream of dry nitrogen at 80 ms delay following pulsed laser excitation (337 nm, 15 ms integration time). The triplet energies were determined using the onset wavelength of the phosphorescence emission band. The two *para*‐conjugated molecules **22Cz** and **33DBS** have significantly lower energy triplets than **33Cz** and **33TXS**, consistent with the shorter conjugation length of the latter two molecules. The singlet and triplet energies in Zeonex are also broadly similar with those determined in solution and by DFT. Uniquely in this series, green room temperature phosphorescence (RTP) lasting over 5 seconds was also observed for **22Cz** in Zeonex film. 1*H*‐Benzo[*f*]indole is a significant contaminant in commercial‐grade carbazole which was recently confirmed as the cause of yellow/orange RTP in various carbazole derivatives.[^57^, ^58^] As the synthetic route for **22Cz** does not employ commercial sources of carbazole, instead relying upon P(OEt)_3_ mediated cyclisation of intermediate **4**, this allows us to disregard the possibility that the RTP arises from residual 1*H*‐benzo[*f*]indole or similar isomeric impurity.

The *meta*‐conjugated **33Cz** was selected for further study as a high triplet (*E*_T_=3.07 eV) host material for blue TADF emitters. In contrast to phosphorescent emitters such as FIrpic (*E*_T_=2.62 eV)^[59]^ the significantly higher triplet energies of blue TADF materials with similar CIE coordinates (a consequence of their broader emission bands) requires the use of hosts with even higher triplet energies.^[13]^ This requirement significantly restricts the range of suitable host materials for blue TADF emitters, as most of the commonly used carbazole‐based hosts have triplet energies that are too low (<3.00 eV).[^25^, ^26^] The prospect of a high triplet carbazole‐based host for blue TADF OLEDs, with associated good charge transport properties endowed by the carbazole moiety, made **33Cz** an attractive candidate to study toward such applications.

**Figure 6:**
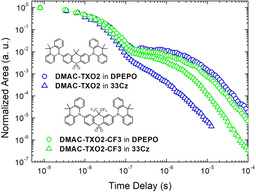
Emission decay profiles for **DMAC‐TXO2** and **DMAC‐TXO2‐CF3** in **DPEPO** and **33Cz** hosts.

**33Cz** was initially screened as a host for the previously reported and well‐studied blue TADF material 2,7‐bis(9,9‐dimethyl‐acridin‐10‐yl)‐9,9‐dimethylthioxanthene‐*S*,*S*‐dioxide **DMAC‐TXO2**.[^19^, ^20^, ^60^, ^61^, ^62^, ^63^] These reports establish the triplet energy of **DMAC‐TXO2** at 2.98 eV, and therefore potentially compatible with **33Cz** (*E*_T_=3.07 eV). A drop‐cast film of **33Cz** doped with 10 wt % **DMAC‐TXO2** was prepared and its time resolved emission spectra and kinetics recorded using previously described methods.[^28^, ^64^] Surprisingly, even though **DMAC‐TXO2** had previously demonstrated excellent TADF performance in other hosts,[^61^, ^65^] the **33Cz** film displayed lower delayed fluorescence (DF) intensity and absence of a clear DF ‘plateau’ in the μs time region. The DF decay kinetics were also far too rapid to attribute to normal rISC (with DF intensity falling below the sensitivity floor of our system after only ≈14 μs), and were instead reminiscent of a triplet quenching mechanism—despite the apparently compatible ordering of host and guest triplet energies. The emission decays for the **33Cz** film are shown in Figure 6, along with representative decays of the same emitter in the established high triplet host bis(2‐(diphenylphosphino)phenyl)ether oxide **DPEPO** (*E*_T_>3.00 eV).^[60]^ The DF emission from **DDMA‐TXO2** in **DPEPO** is consistent with the successful use of this host in high efficiency OLEDs.^[19]^

Furthermore, OLEDs fabricated using **33Cz** as a host for **DMAC‐TXO2** showed surprisingly poor performance. Modifying a previously reported device architecture (that had given maximum external quantum efficiencies of ≈20 %, using **DPEPO** as EML host and methods previously reported),^[60]^ **DMAC‐TXO2** devices in **33Cz** host gave at most ≈4.7 % EQE. This value is consistent with the TADF material acting as an efficient fluorescent dopant, but with no active triplet harvesting. The specific stack used was ITO|NPB (40 nm) | TCBPA (10 nm) | **DMAC**‐**TXO2** (13 %) in **33Cz** (20 nm) | TPBi (40 nm) | LiF/Al. Neither replacing the TCBPA with TAPC, nor varying the emissive layer thickness (from 20 to 40 nm) resulted in any improvement in the performance.

Taken in isolation, it is difficult to draw insight from the poor performance of the **33Cz**‐hosted TADF‐OLEDs. Many factors can contribute to an OLED structure giving poor performance, with promising host materials occasionally proving to be intrinsically incompatible with electrical excitation. In this case though, additional OLEDs fabricated using **33Cz** as host for a sky‐blue phosphorescent mesitylene‐substituted FIrpic derivative^[66]^ (structure shown in Figure S1) demonstrate that this is not the case for **33Cz**. These PhOLEDs were found to perform approximately as expected based on the previous report of this emitter using a thermally evaporated stack of ITO | NPB (40 nm) | TAPC (10 nm) | mesitylene‐FIrpic (12 %) in **33Cz** (30 nm) | TPBi (40 nm) | BCP (8 nm) | LiF/Al. These devices gave respectable performance with maximum EQE=5.4 %, which is less than the maximum value achieved in the initial report of this emitter (10.4 % in solution processed devices),^[66]^ but toward the upper end of the range reported for different stacks and dopant concentrations. In any case, this level of performance is certainly large enough to demonstrate that **33Cz** has the capacity to act as a functional OLED host for appropriate dopants. The poor performance of the **DMAC‐TXO2** doped **33Cz** devices therefore cannot be attributed solely to the hosting properties of **33Cz** itself. The triplet energy of the phosphorescent mesitylene‐FIrpic dopant was estimated from the reported electroluminescence spectrum at 2.72 eV, well below that of **DMAC‐TXO2** and of **33Cz** (in both Zeonex and neat films).

In addition, **33Cz** was screened as a host for the recently reported TADF material **DMAC‐TXO2‐CF3**. This emitter, with trifluoromethyl substituents on the 9‐position of the thioxanthene ring, exhibits green emission and possess a lower triplet energy (2.78 eV)^[62]^ than **DMAC‐TXO2**. Both properties are a result of the increased acceptor strength and enhanced CT character arising from the strong inductively withdrawing CF_3_ groups. In contrast to the blue **DMAC‐TXO2**, in photoluminescence measurements the structurally analogous **DMAC‐TXO2‐CF3** was found to give strong DF with reasonable decay kinetics in both **DPEPO** and **33Cz**, with no indication of triplet quenching in the DF region (Figure 6). Together with the low performance of the TADF‐OLEDs, these results stimulated us to reconsider the previously recorded high triplet energy of **33Cz**, stated above. Indeed, in conflict with the prior Zeonex measurements, these decay kinetics and device results are consistent with a **33Cz** triplet energy between that of **DMAC‐TXO2** (2.98 eV) and **DMAC‐TXO2‐CF3** (2.78 eV).

**Figure 7:**
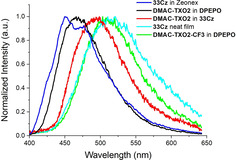
Phosphorescence of **DMAC‐TXO2** in **33Cz** and **DPEPO** (10 wt %), as well as from neat **33Cz**, **33Cz** in Zeonex (1 wt %), and **DMAC‐TXO2‐CF3** in **DPEPO** (10 wt %). In all cases emission was collected at cryogenic temperatures (80 K or lower), and long delay times (70 ms or longer) following pulsed excitation (337 or 355 nm).

That the triplet energy of **33Cz** measured in Zeonex is inappropriate for guiding OLED design was further demonstrated by low temperature phosphorescence measurements of a **DMAC‐TXO2‐**doped **33Cz** film (Figure 7). Instead of phosphorescence with the expected onset of 2.98 eV (from the **DMAC**‐**TXO2** guest) emission was recorded from an unknown species with a triplet energy of only 2.83 eV. Crucially, at this stage phosphorescence was also recorded for a drop‐cast neat film of **33Cz**, which demonstrated significantly different behaviour than the same material in Zeonex host. Instead, the phosphorescence from the neat film of **33Cz** closely matches that of the **DMAC‐TXO2** doped film, revealing that the unknown species is in fact the **33Cz** host, which takes on a significantly lowered triplet energy when in neat film. Additional phosphorescence spectra from the **33Cz** neat film, **33Cz** Zeonex film, and **DMAC‐TXO2‐CF3** in **DPEPO** are included in Figure 7 for comparison.

Ultimately, the contrasting performances of **DMAC‐TXO2** and **DMAC‐TXO2‐CF3** in **33Cz** as host demonstrate that the lower triplet energy inferred from phosphorescence of the **33Cz** neat film is the relevant value to consider for device applications. This assertion is further supported by the contrasting performance of **33Cz** OLEDs using high‐triplet TADF or lower‐triplet phosphorescent emissive dopants. Evidently, earlier Zeonex measurements gave a higher but ultimately inappropriate value in the present and in previous work.^[24]^ We expect that phosphorescence measurements in other polymer hosts or in frozen solvents, where the ‘host material’ of interest is itself dispersed in another host, can lead to similarly overestimated triplet energies of novel OLED host materials, and should be avoided. As illustrative examples, phosphorescence measurements from neat films of host materials **BCPO**,^[67]^ **2CzCbPy**,^[68]^ and **PPBi** ^[69]^ (Figure S9) performed in our laboratories indicate significantly lower triplet energies (2.72, 2.93, and 3.01 eV, respectively) than in the original reports of these materials from frozen solution (3.01, 2.97, and 3.30 eV, respectively). The revised lower *E*_T_ value for **PPBi** and consequent potential for host quenching of deep‐blue TADF triplets may be a contributing factor to the generally lower device EQEs achieved with this host compared to phosphine‐oxide‐based hosts.[^69^, ^70^] Similar differences in measurement technique are also likely responsible for the diversity of triplet energies reported for **DPEPO**.[^60^, ^71^, ^72^]

This effect of film composition on phosphorescence has been observed previously by others for **CBP** and its twisted analogue 4,4’‐bis(9‐carbazolyl)‐2,2’‐dimethylbiphenyl **CDBP**,[^27^, ^54^, ^73^] as well as several other materials.[^74^, ^75^] However, despite the logical implications for triplet energies and how these determine host/guest compatibility, it is not commonplace to report neat film phosphorescence of new OLED host materials. Instead, low temperature solution measurements currently dominate the literature in reports of this kind.[^69^, ^76^, ^77^, ^78^, ^79^, ^80^, ^81^, ^82^, ^83^, ^84^, ^85^, ^86^, ^87^] A recent study by Forrest and Thompson^[73]^ has demonstrated that such solution measurements do not give appropriate triplet energies for the design of OLEDs, while the results here show that phosphorescence in dilute polymer films is also not suitable in this regard. We therefore suggest that neat film phosphorescence measurements should become the accepted standard for reporting *E*_T_ of novel OLED host materials. The scarcity of truly high *E*_T_ host materials appropriate for blue and deep‐blue TADF‐OLEDs makes correct reporting of new hosts all the more crucial.

Following these insights for **33Cz**, neat films of **22Cz**, **33Cz**, **33DBS** and **33TXS** were prepared by drop‐casting and their photoluminescence was re‐measured for comparison with their Zeonex films (shown in Figure 5). For all four molecules there is a significant red‐shift in the phosphorescence onsets and the spectrum when moving to neat films, indicating a lowering of *E*_T_. For the example of **DDMA‐TXO2** and **33Cz**, this clearly has catastrophic consequences on its potential as a host for blue TADF‐OLEDs. These results are indicative of packing effects in the solid‐state, which distort the geometries of the molecules and in this case lead to increased planarization with a consequential decrease in *E*_T_ across the series. Despite an overall drop of 0.21 eV **33TXS** retains a comparatively high *E*_T_ and spectral shape is preserved between solution, Zeonex, and neat films. We attribute this to the central biphenyl chromophore being more effectively protected from major conformational changes due to steric demands of the non‐conjugated terminal phenyl rings.

Interestingly, the red‐shift in the phosphorescence spectrum on moving to neat films is considerably smaller for **22Cz** than for **33Cz**. Considering this alongside the DFT results, the reduced influence of concentration is attributed to the extended linear conjugation of *para*‐linked **22Cz** compared to *meta*‐linked **33Cz**. The triplet state of **22Cz** is stabilized by delocalization along the backbone of the molecule, yielding a “whole‐molecule” triplet state, as opposed to a characteristic triplet state wavefunction of an individual carbazole unit,^[40]^ as in **33Cz**. While the extensively delocalized “whole‐molecule” triplet wavefunction will have a lower intrinsic *E*_T_, it also appears to be more resilient to the host packing effects that reduce *E*_T_ of **33Cz** in neat film. This is because the electronic coupling between carbazole units is already large for **22Cz**, and so it cannot increase significantly upon further planarization. In contrast, **33Cz** has almost no coupling across the central bridging bond, but this can increase dramatically upon packing‐induced planarization, leading to a large change in the nature of the electronic state and its energy. Also arising from the difference in their connectivity, **33Cz** requires a less restrictive environment (larger free volume) to accommodate geometry changes associated with transitions between excited states. Rod‐like **22Cz** can in contrast undergo bridge rotations without changing its shape significantly, thus requiring a smaller surrounding free volume to transition between states.

Confirming this structural analysis, further scrutiny of the TDDFT results (Tables S5–S8) revealed that the oscillator strength for the S_0_→S_1_ transition in **33Cz** was very small at 0.008, compared to 0.304 for **22Cz**. The S_0_→S_3_ transition was the first intense transition at 0.670 for **33Cz** and at 0.625 for **22Cz**. Calculated structures of the S_3_ and S_1_ states for both dimers displayed significant changes in bridge dihedral angle upon excitation, both becoming more planar as summarized in Table S9. By applying geometry constraints to **33Cz** the variation in *E*_T_ as the dihedral angle was taken from 0° to 180° was also obtained (Figure S7). This clearly shows that the *E*_T_ is suppressed as the molecule becomes co‐planar. *E*_T_ increases by 0.30 eV moving from 0° to 90° then drops by 0.27 eV moving to 180°, with a graphical shape evocative of Mt Fuji (c.f. Figure S8 and TOC graphic). Variations in *E*_S_ and *E*_g_ are similar. S_1_ structures were also calculated for **33DBS** and **33TXS** also show tighter dihedral angles in the excited state indicating that trend is consistent across the series.

Reiterating, both **22Cz** and **33Cz** planarize to different extents upon excitation; however, as **22Cz** is a linear and more compact molecule, the changes in dihedral angle between the carbazole rings will result in simple twisting motions about the central bond. This minimizes the impacts that the restrictive environment of the neat film has on its optoelectronic properties. For the non‐linear **33Cz** a scissoring motion will occur upon excitation, which requires more space to execute without hindrance. Consequently, the energies of the electronic states in **33Cz** are more sensitive to packing or aggregation effects in the neat film as this scissoring motion is inhibited. This insight reveals that developing high *E*_T_ molecular materials that rely on twisted structures also requires that this twist be held in place by extremely rigid substituents or linking groups. Otherwise, packing‐associated planarization or restriction of molecular motion will lead to significant variation in the *E*_T_ of neat films as is observed in the present series.

While intrinsically planar OLED hosts might be expected to be exempt from this planarizing effect, similar effects on phosphorescence have previously been reported in both planar **CBP** and its twisted analog 4,4’‐bis(9‐carbazolyl)‐2,2’‐dimethylbiphenyl (**CDBP**).^[27]^ For these systems the effect on *E*_T_ in neat film was attributed to different propensities to form excimers, also arising from differences between delocalized “whole‐molecule” and localized “single‐side” triplet state wavefunctions. For both mechanisms, the identification that “whole‐molecule” conjugation leads to more stable *E*_T_ has useful implications in further EML host design—although it may be difficult to balance that advantage with the unavoidably lower *E*_T_ associated with large conjugated systems.

Packing‐induced changes in the bridge dihedral angles of our host materials will not uniformly influence the entire ensemble of molecules in the neat film. Some molecules will exist in loose packing environments similar to those found in Zeonex films, retaining highly twisted bridges and high individual singlet and triplet energies. Other molecules will have their excited state energies significantly lowered by increased planarization. This expected structural inhomogeneity will lead to a distribution of bridge dihedral angles and associated singlet and triplet energies in the film, and evidence for this distribution is found in the emission spectra. As well as the discussed changes in the phosphorescence spectrum, we also observed red‐shifts in fluorescence when moving from Zeonex to neat films, accompanied by the appearance of an extended red emission tail (Figure 5). This tail emission is attributed to the more significantly planarized molecules in the neat films, which emit at lower energies and longer wavelengths due to their increased conjugation length. As the steady‐state fluorescence spectrum is not time‐gated, contributions from both the twisted and planarized molecules are observed simultaneously in the film. The net result is a fluorescence spectrum with similar onset and peak wavelength (coming from the molecules that remain twisted, as in Zeonex), as well as a red‐shifted tail—or in the case of **33Cz**, a secondary emission peak at around 480 nm from the planarized molecules. In time‐gated phosphorescence measurements emission from only the lowest energy planarized triplet states is observed, as triplet diffusion in the film allows long‐lived excitons to find the lowest energy sites before emission.

Within the series of compounds **33TXS** displays the smallest change in fluorescence and phosphorescence profile upon moving from Zeonex to neat films and retains a high *E*_T_. Consistent with the explanations above, for **33TXS** the non‐conjugated terminal phenyl rings occupy a significant volume, which helps to shield the central biphenyl group from matrix effects and allows it to dictate the photophysical properties of the molecule. Taking advantage of its intrinsically high *E*_T_ biphenyl core (elevated yet higher by twisting), further modification of this structural motif may allow high *E*_T_ values to be achieved regardless of changes in molecular conformation.

Finally, we note that our neat film phosphorescence measurements have been performed on drop‐cast films. While in our experience we do not observe significant differences in *E*_T_ (or emission decay kinetics) to arise from different film deposition methods, measurements of neat thermally evaporated films are strictly the most appropriate for determining host *E*_T_ values guiding the design of evaporated OLEDs. Nonetheless, the convenience of solution‐processing makes it a useful tool for screening new host materials. Similar arguments of convenience likely also explain the widespread use of frozen‐solution and polymer film measurements—practices that this work demonstrates must be critically reassessed.

## Conclusions

A new series of high *E*_T_ molecules has been synthesized displaying large dihedral angles enforced between two heterocycles, with the *meta*‐conjugated carbazole dimer **33Cz** displaying the highest *E*_T_ of 3.07 eV in dilute polymer film. For the linearly conjugated **22Cz** and **33DBS** with *para*‐quaterphenyl backbones the HOMO–LUMO gap is controlled by the long conjugation length of the molecule, but the relative energies of the HOMO and LUMO themselves are controlled by the nature of the heteroatom in the cycle. Upon using **33Cz** as a host material for a blue TADF emitter we identify that environmental and packing effects on *E*_T_ (impacting the conformational freedom of the molecule) can confound attempts to predict host/guest compatibility. In neat film the *E*_T_ of **33Cz** drops to 2.83 eV, a decrease of 0.24 eV compared to Zeonex films. This change in *E*_T_ precludes **33Cz** as a suitable host for blue TADF dopants with *E*_T_>3.00 eV. Comparing across the other materials in the series, these packing effects can be further exacerbated by the linkage pattern of the dimer (*meta*>*para*) and the presence of sterically shielding groups. While a pre‐twisted structure is therefore found to be suitable for designing high *E*_T_ materials, we identify that the structure must also be sufficiently compact and/or rigid to prevent packing‐induced planarization exerting detrimental downwards pressure on *E*_T_. These points are demonstrated well by **33TXS**, where the terminal phenyl rings are not conjugated to the biphenyl core and serve to partially protect the biphenyl from packing effects in the neat film.

Finally, this work highlights that to reliably estimate host/guest compatibility, *E*_T_ determined from phosphorescence of neat films must be reported for new host materials. The currently accepted practice of reporting values from frozen solution or polymer films is no substitute.

## Experimental Section

Additional experimental details, supporting figures, packing diagrams and single‐crystal data, calculation details, and NMR spectra are provided in the Supporting Information. Deposition numbers 1999181, 1999182, 1999183, 1999184, and 1999185 contain the supplementary crystallographic data for this paper. These data are provided free of charge by the joint Cambridge Crystallographic Data Centre and Fachinformationszentrum Karlsruhe Access Structures service www.ccdc.cam.ac.uk/structures.

## Conflict of interest

The authors declare no conflict of interest.

## Supporting information

As a service to our authors and readers, this journal provides supporting information supplied by the authors. Such materials are peer reviewed and may be re‐organized for online delivery, but are not copy‐edited or typeset. Technical support issues arising from supporting information (other than missing files) should be addressed to the authors.

Supplementary
