# Supplementary material for: Conformational Dependence of Triplet Energies in Rotationally Hindered N‐ and S‐Heterocyclic Dimers: New Design and Measurement Rules for High Triplet Energy OLED Host Materials
Source: Chemistry. 2021 Mar 10;27(21):6545–56. doi: 10.1002/chem.202100036 (PMC8251716; doi:10.1002/chem.202100036)
Supplement: Supplementary file 1 — Supplementary [file CHEM-27-6545-s001.pdf]

Chemistry–A European Journal

Supporting Information

Conformational Dependence of Triplet Energies in Rotationally Hindered N- and S-Heterocyclic Dimers: New Design and Measurement Rules for High Triplet Energy OLED Host Materials

Iain A. Wright,*[a, b] Andrew Danos,[c] Stephanie Montanaro,[b] Andrei S. Batsanov,[a] Andrew P. Monkman,*[c] and Martin R. Bryce*[a]

Supporting Information

Conformational Dependence of Triplet Energies in Rotationally-Hindered *N*- and *S*-Heterocyclic Dimers: New Design and Measurement Rules for High Triplet Energy OLED Host Materials

Iain A. Wright,*[a,b] Andrew Danos,[a] Stephanie Montanaro,[b] Andrei S. Batsanov,[a] Andrew P. Monkman*[c] and Martin R. Bryce*[a]

[a] *Department of Chemistry, Durham University, South Road, Durham, DH1 3LE, UK*

[b] *Department of Chemistry, Loughborough University, Loughborough, Leicestershire, LE11 3TU, UK*

[c] *Department of Physics, Durham University, South Road, Durham, DH1 3LE, UK*

Table of Contents

Synthetic Methods **S2**

Figure S1 Structures of Other Materials Mentioned in this Work **S12**

Optoelectronic Methods **S13**

X-Ray Crystallography **S14**

Computational Results **S21**

FigureS9 Neat Film Phosphoresence of Other Hosts **S26**

Copies of NMR Spectra **S27**

Synthetic Methods

General experimental

All commercially available chemicals were used without further purification. Reactions requiring an inert atmosphere were performed under a blanket of argon gas, which was dried over a phosphorus pentoxide column. Anhydrous solvents were dried through an HPLC column on an Innovative Technology Inc. solvent-purification system. Column chromatography was performed using 40–60 µm mesh silica gel. Analytical thin-layer chromatography (TLC) was performed on plates precoated with silica gel (Merck, silica gel 60F254) and visualized using UV light (254, 315, 365 nm). NMR spectra were recorded on Bruker Avance 400 MHz and Varian Mercury 400 MHz spectrometers. Melting points were determined in open-ended capillaries using a Stuart Scientific SMP3 melting point apparatus at a ramping rate of 1 °C/min. Atmospheric solids analysis probe (ASAP) mass spectra were recorded on a Waters Xevo QTOF spectrometer. Elemental analyses were obtained on an Exeter Analytical Inc. CE-440 elemental analyzer.

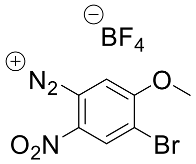

4-Bromo-5-methoxy-2-nitrobenzenediazonium tetrafluoroborate (3)

4-Bromo-5-methoxy-2-nitroaniline **2** (3.09 g, 12.5 mmol) was dissolved in CH_2Cl_2 (25 mL) and the solution was cooled in an ice bath to 0 °C with stirring. $BF_3.OEt_2$ (1.9 mL, 15.0 mmol) was added slowly to followed immediately by *t*-BuONO (90%, 2.22 mL, 18.75 mmol). After 5 minutes the cooling bath was removed and the reaction was left to stir overnight. The resulting suspension was filtered through a glass sinter and the solid washed with CH_2Cl_2 to give the crude diazonium tetrafluoroborate **3** (4.34 g, 99%). The crude product is ready to use, however, it can be further purified by dissolving in MeCN and stirring with activated charcoal for 15 min prior to removal of the charcoal by filtration through celite and precipitation of the diazonium tetrafluoroborate by addition of Et_2O. The pure salt can then be isolated by filtration and dried under air.

^{1}H-NMR (400 MHz, CD_3CN): δ = 8.91 (s, 1H), 8.33 (s, 1H), 4.17 (s, 1H)

^{19}F-NMR (376 MHz, CD3CN) δ = -151.5

^{11}B-NMR (128 MHz, CD3CN) δ = -1.21

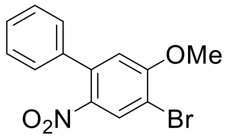

4-Bromo-5-methoxy-2-nitro-1,1'-biphenyl (4)

Under air, diazonium tetrafluoroborate **3** (2.50 g, 7.20 mmol) and phenylboronic acid (0.88 g, 7.20 mmol) were suspended in 1,4-dioxane (25 mL). $Pd(OAc)_2$ (5 mol%, 30 mg, 0.13 mmol) was added and the reaction was stirred for 3 h at room temperature prior to removal of solvent under reduced pressure.

Purification was achieved by column chromatography on SiO_2 (0 to 10% EtOAc/hexane) to give **4** as a pale yellow solid (2.03 g, 91 %). mp. 112–113 °C

^{1}H-NMR (400 MHz, $CDCl_3$): δ = 8.24 (s, 1H), 7.48 – 7.39 (m, 3H), 7.30 (dd, J = 6.5, 2.9 Hz, 2H), 6.82 (s, 1H), 3.98 (s, 3H)

^{13}C-NMR (101 MHz, $CDCl_3$): δ = 158.9, 141.9, 138.7, 137.7, 130.1, 128.8 (2C), 128.6, 127.8 (2C), 114.2, 110.6, 57.0

MS (ASAP): m/z = 308.0 $[M+H]^+$

HRMS (ASAP): m/z = calculated for $C_{13}H_{10}BrNO_3$ $[M]^+$: 306.9844; found: 306.9845

Elemental analysis: Found C, 50.73; H, 3.29; N, 4.52 Calculated C, 50.67; H, 3.27; N, 4.55

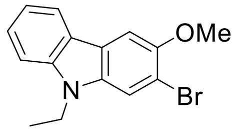

2-Bromo-9-ethyl-3-methoxy-9*H*-carbazole (5)

The cyclization and alkylation of **4** (1.85 g, 6.00 mmol) to **5** was performed analogously to the synthesis of **1**.

Purification was achieved by column chromatography on SiO_2 (0 to 10% EtOAc/hexane) to give **5** as a colorless crystalline solid (0.97 g, 53 % over two steps). mp. 114–115 °C

^{1}H-NMR (400 MHz, $CDCl_3$): δ = 8.04 (d, J = 7.9 Hz, 1H), 7.61 (s, 1H), 7.60 (s, 1H), 7.48 (ddd, J = 8.2, 7.1, 1.2 Hz, 1H), 7.38 (d, J = 8.2 Hz, 1H), 7.21 (ddd, J = 7.9, 7.1, 1.2 Hz, 1H), 4.31 (q, J = 7.2 Hz, 2H), 4.02 (s, 3H), 1.42 (t, J = 7.2 Hz, 3H)

^{13}C-NMR (101 MHz, $CDCl_3$): δ = 149.8, 140.5, 135.3, 126.1, 122.7, 122.5, 120.4, 118.9, 113.3, 110.7, 108.9, 103.6, 57.3, 37.9, 14.0

MS (ASAP): m/z = 304.0 $[M+H]^+$

HRMS (ASAP): m/z = calculated for $C_{15}H_{14}BrNO$ $[M+H]^+$: 303.0259; found: 303.0270

Elemental analysis: Found C, 59.37; H, 4.66; N, 4.77 Calculated C, 59.23; H, 4.64; N, 4.60

N.B. It is worth noting that although the NMR spectra are in good agreement with previously reported data, the melting points measured for intermediates **4** (112–113 °C, previously 90–91 °C) and **5** (114–115 °C, previously 85.5–87.5 °C) are substantially higher than those reported previously.[1]

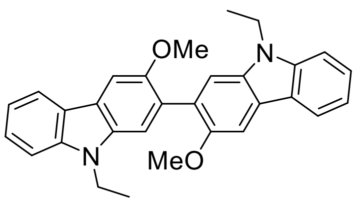

9,9'-Diethyl-3,3'-dimethoxy-9*H*,9'*H*-2,2'-bicarbazole (22Cz) Under an atmosphere of nitrogen **5** (740 g, 2.42 mmol) and Pd-PEPPSI-*i*Pr (5 mol%, 82 mg, 0.12 mmol) were dissolved in dry toluene (40 mL) and degassed with stirring for 10 minutes. To this stirred mixture at room temperature was added a solution of *tert*-butyllithium (1.7 M in pentane, 1.14

mL, 1.94 mmol) over 1 h. The reaction was left to stir overnight then quenched with MeOH before the solvent was removed under reduced pressure. The residue was redissolved in CH_2Cl_2 (50 mL) and transferred to a separating funnel before being washed with water (100 mL). The aqueous phase was extracted with a further portion of CH_2Cl_2 (2 × 50 mL) and the combined organic phases were dried over $MgSO_4$ which was removed by filtration prior to evaporation of the solvent under reduced pressure.

Purification was achieved by passing the material through a short column of SiO_2 (eluting with EtOAc) to give crude **22Cz** as an off-white powder. Recrystallization from EtOAc yielded **22Cz** as white needles (457 mg, 84%). mp. 242–244 °C

^{1}H-NMR (400 MHz, $CDCl_3$): δ = 8.11 (d, J = 7.9 Hz, 2H), 7.72 (s, 2H), 7.47 (ddd, J = 8.1, 7.1, 1.1 Hz, 2H), 7.43 (s, 2H), 7.41 (d, J = 8.1 Hz, 2H), 7.22 (ddd, J = 7.9, 7.1, 1.0 Hz, 1H), 4.37 (q, J = 7.2 Hz, 4H), 3.92 (s, 6H), 1.45 (t, J = 7.2 Hz, 6H)

^{13}C-NMR (101 MHz, $CDCl_3$): δ = 151.8, 140.6, 134.9, 128.4, 125.5, 123.1, 122.6, 120.4, 118.4, 111.5, 108.6, 103.0, 57.0, 37.8, 14.1

MS (ASAP): m/z = 449.2 $[M+H]^+$

HRMS (ASAP): m/z = calculated for $C_{30}H_{29}N_2O_2$ $[M+H]^+$: 449.2189; found: 449.2216

Elemental analysis: Found C, 79.71; H, 6.24; N 6.10, Calculated C, 80.33; H, 6.29; N, 6.25

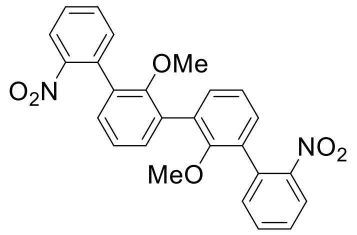

2-Methoxy-3-{2-methoxy-2'-nitro-[1,1'-biphenyl]-3-yl}-2'-nitro-1,1'-biphenyl (7)

Under an atmosphere of N_2 diboronic acid **6** (1.00 g, 3.30 mmol), 1-bromo-2-nitrobenzene (1.47 g, 7.29 mmol), and $Pd(PPh_3)_4$ (8 mol%, 0.30 g, 0.26 mmol) were added to a stirred mixture of thoroughly degassed toluene (75 mL) and EtOH (25 mL). Solid K_2CO_3 (2.28 g, 16.5 mmol) was then added and the reaction was heated to 95 °C for 16 h. The reaction mixture was filtered through fluted filter paper while still hot and any solid residues were then washed by passing EtOAc (100 mL) through the filter paper. H_2O (200 mL) was then added before the mixture was transferred to a separating funnel. The aqueous layer was removed and the organic phase washed with more H_2O (2 × 100 mL) and brine before being dried over $MgSO_4$. The solvent was removed under reduced pressure and the residue was recrystallized repeatedly from acetone to yield **7** as an off-white solid (0.84 g, 56%). mp. 194–196 °C

^{1}H-NMR (400 MHz, $CDCl_3$): 8.01 (dd, J = 8.4, 1.3 Hz, 2H), 7.68 (td, J = 7.6, 1.3 Hz, 2H), 7.56 – 7.50 (m, 4H), 7.44 (dd, J = 7.4, 2.0 Hz, 2H), 7.31 (dd, J = 7.5, 2.0 Hz, 2H), 7.26 (t, J = 7.5 Hz, 2H), 3.22 (s, 6H)

^{13}C-NMR (101 MHz, DMSO-*d*6): δ = 154.2, 148.9, 133.4, 132.5, 132.2, 131.8, 131.3, 131.0, 129.5, 129.0, 124.1, 124.0, 60.1

MS (ASAP): m/z = 457.1 $[M+H]^+$

HRMS (ASAP): m/z = calculated for $C_{26}H_{20}N_2O_6$ $[M]^+$:456.1321; found: 456.1325

Elemental analysis: Found C, 68.39; H, 4.50; N, 5.97 Calculated C, 68.42; H, 4.42; N, 6.14

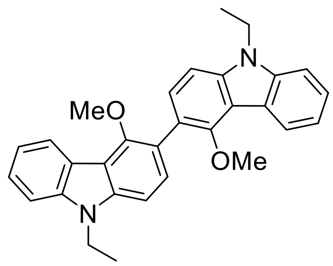

9,9'-Diethyl-4,4'-dimethoxy-9*H*,9'*H*-3,3'-bicarbazole (33Cz)

Compound **7** (456 mg, 1.00 mmol) was added to a microwave vial containing freshly distilled $P(OEt)_3$ (4.35 mL, 25.0 mmol) and a magnetic stirrer bar. The vial was sealed with a Teflon cap before being transferred to a microwave reactor and heated to 200 °C under microwave irradiation with stirring for 20 minutes. Upon cooling the vial was opened and the reaction mixture was poured into a 100 mL conical flask. 2 M HCl (30 mL) was then added cautiously and the reaction mixture was stirred for 1 h in order to quench the $P(OEt)_3$. The resulting aqueous suspension was transferred to a separating funnel and then extracted with EtOAc (3 × 30 mL). The combined organic extracts were washed with H_2O (50 mL) before drying over $MgSO_4$ and removal of solvent under reduced pressure.

The residue thus obtained was then dissolved in degassed DMSO (10 mL) under a nitrogen atmosphere alongside ethylbromide (0.30 mL, 4.00 mmol) and few crystals of KI. KOH (449 mg, 8.00 mmol) was then added and the reaction was allowed to stir overnight at room temperature. The reaction was quenched with H_2O (50 mL) and then extracted with EtOAc (3 × 30 mL). The combined organic extracts were then washed with 2M HCl (30 mL) and H_2O (3 × 30 mL) before being dried over $MgSO_4$. The solvent was then removed under reduced pressure.

Purification was achieved by column chromatography on SiO_2 (eluting 10% EtOAc/hexane) to give pure **33Cz** as a white solid (217 mg, 48% over two steps). If required, further purification may be achieved by recrystallization from a mixture of EtOAc and hexane. mp. 206–208 °C

^{1}H-NMR (400 MHz, $CDCl_3$): δ = 8.39 (d, J = 7.7 Hz, 2H), 7.58 (d, J = 8.3 Hz, 2H), 7.49 (ddd, J = 7.9, 6.9, 1.2 Hz, 2H), 7.44 (d, J = 7.9 Hz, 2H), 7.30 – 7.26 (m, 2H), 7.26 – 7.23 (m, 1H), 4.42 (q, J = 7.2 Hz, 2H), 3.70 (2, 6H), 1.51 (t, J = 7.2 Hz, 3H)

^{13}C-NMR (101 MHz, $CDCl_3$): δ = 153.9, 141.2, 139.8, 129.9, 125.2, 123.3, 121.9, 121.8, 119.1, 116.0, 108.1, 104.0, 60.1, 37.8, 13.9

MS (ASAP): m/z = 449.2 $[M+H]^+$

HRMS (ASAP): m/z = calculated for $C_{30}H_{29}N_2O_2$ $[M+H]^+$: 449.2229; found: 449.2222

Elemental analysis: Found C, 80.08; H, 6.29; N, 6.32 Calculated C, 80.33; H, 6.29; N, 6.25

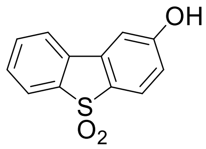

2-Hydroxydibenzothiophene-*S*,*S*-dioxide (9)

Dibenzothiophene-2-boronic acid **9** (5.00 g, 22 mmol) was dissolved in AcOH with stirring (200 mL) and was heated to 60 °C for 1 h then 125 °C overnight. After cooling to approximately 60 °C, the mixture was poured over ice to precipitate analytically pure **9** as a pale yellow powder which was isolated by filtration, washed with copious amounts of water and dried under air. The material was recrystallized from a mixture of EtOH and H_2O as small pale-yellow crystals (3.68 g, 72%). mp. 256–258 °C

^{1}H-NMR (400 MHz, DMSO-*d*6): δ = 10.82 (s, 1H), 8.09 (d, J = 7.7 Hz, 1H), 7.91 (d, J = 7.5 Hz, 1H), 7.77 (d, J = 8.4 Hz, 1H), overlapping with 7.79 – 7.73 (m, 1H), 7.63 (td, J = 7.6, 0.9 Hz, 1H), 7.47 (d, J = 2.2 Hz, 1H), 6.98 (dd, J = 8.4, 2.2 Hz, 1H)

^{13}C-NMR (101 MHz, acetone-*d*6): δ = 162.9, 138.2, 134.2, 133.5, 130.8, 130.7, 127.3, 123.8, 122.5, 121.6, 117.2, 109.0

MS (ASAP): m/z = 233.0 $[M+H]^+$

HRMS (ASAP): m/z = calculated for $C_{12}H_9O_3S$ $[M+H]^+$: 233.0272; found: 233.0273

Elemental analysis: Found C, 62.22; H, 3.53; N, 0.06 Calculated C, 62.06; H, 3.47; N, 0.00

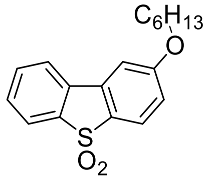

2-(Hexyloxy)dibenzothiophene-*S*,*S*-dioxide (10)

Under air, 9 (1.00, 4.31 mmol) was dissolved in DMF (50 mL) prior to addition of K_2CO_3 (1.20, 8.61 mmol). The reaction was then stirred for 30 minutes. Hexyl bromide (0.91 mL, 16.47 mmol) was added and the reaction was allowed to continue stirring at room temperature overnight. The reaction was quenched with H_2O (50 mL) and then extracted with EtOAc (3 × 20 mL). The combined organic extracts were then washed with 2M HCl (20 mL) and H_2O (3 × 30 mL) before being dried over $MgSO_4$. The solvent was then removed under reduced pressure and the residue was recrystallized from MeOH to yield **10** as an off-white powder (1.02 g, 75%). mp. 111–112 °C

^{1}H-NMR (400 MHz, $CDCl_3$): δ = 7.81 (d, J = 7.6 Hz, 1H), 7.75 (d, J = 7.6 Hz, 1H), 7.73 (d, 8.5 Hz, 1H), 7.63 (td, J = 7.6, 1.1 Hz, 1H), 7.53 (td, J = 7.6, 1.0 Hz, 1H), 7.24 (d, J = 2.2 Hz, 1H), 6.98 (dd, J = 8.5, 2.2 Hz, 1H), 4.08 (t, J = 6.5 Hz, 2H), 1.90 – 1.77 (m, 2H), 1.53 – 1.44 (m, 2H), 1.40 – 1.31 (m, 4H) 0.92 (t, J = 7.1 Hz, 3H)

^{13}C-NMR (101 MHz, $CDCl_3$): δ = 164.0, 139.0, 134.1, 133.8, 131.6, 130.6, 129.6, 123.9, 122.2, 121.6, 115.9, 107.8, 69.0, 31.7, 29.1, 25.8, 22.7, 14.2

MS (ASAP): m/z = 317.1 $[M+H]^+$

HRMS (ASAP): m/z = calculated for $C_{18}H_{21}O_3S$ $[M+H]^+$: 317.1211; found: 317.1214

Elemental analysis: Found C, 68.10; H, 6.41; N, 0.01 Calculated C, 68.33; H, 6.37; N, 0.00

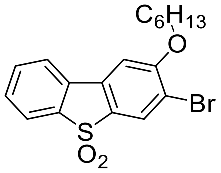

3-Bromo-2-(hexyloxy)dibenzothiophene-*S*,*S*-dioxide (11)

NBS (0.58 g, 3.25 mmol) was added in portions to a stirred solution of **10** (1.01 g, 3.20 mmol) in a mixture of trifluoroacetic acid (27 mL) and H_2SO_4 (3 mL) and the reaction was left to stir overnight at room temperature. The reaction mixture was then poured over ice and the resulting precipitate of crude **11** was isolated by filtration and washed with copious amounts of water.

Purification was achieved by recrystallization from a mixture of toluene and methanol to give pure **11** as white crystals (0.67 g, 53%). mp. 181–183 °C

^{1}H-NMR (400 MHz, $CDCl_3$): δ = 7.96 (s, 1H), 7.79 (d, J = 7.5 Hz, 1H), 7.73 (d, J = 7.6 Hz, 1H), 7.62 (td, J = 7.6, 1.1 Hz, 1H), 7.54 (td, J = 7.5, 0.9 Hz, 1H), 7.16 (s, 1H), 4.16 (t, J = 6.4 Hz, 2H), 1.96 – 1.86 (m, 2H), 1.60 – 1.50 (m, 2H), 1.43 – 1.33 (m, 4H), 0.93 (t, J = 7.0 Hz, 3H)

^{13}C-NMR (101 MHz, $CDCl_3$): δ = 160.2, 138.5, 133.9, 133.0, 131.0, 130.8, 127.2, 122.3, 121.4, 114.4, 104.9, 70.1, 31.6, 29.0, 25.7, 22.7, 14.2

MS (ASAP): m/z = 395.0 $[M+H]^+$

HRMS (ASAP): m/z = calculated for $C_{18}H_{20}O_3SBr$ $[M+H]^+$: 395.0317; found: 395.0323

Elemental analysis: Found C, 54.64; H, 4.81; N, -0.06 Calculated C, 54.69; H, 4.84; N, 0.00

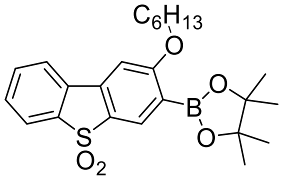

2-Hexyloxy-3-(4,4,5,5-tetramethyl-1,3,2-dioxaborolan-2-yl)dibenzothiophene-*S*,*S*-dioxide (12)

Under an atmosphere of N_2, **11** (500 mg, 1.26 mmol), bis(pinacolato)diboron (480 mg, 1.89 mmol), $Pd(dppf)Cl_2.CH_2Cl_2$ (5 mol%, 57 mg, 0.07 mmol) and KOAc (310 mg, 3.15 mmol) were dissolved in anhydrous DMF (20 mL) and the reaction mixture was heated to 80 °C for 14 h. Upon cooling the reaction mixture was filtered and the solids washed with $CHCl_3$ (50 mL). The combined filtrates were transferred to a separating funnel and washed with H_2O (4 × 50 mL). The organic phase was dried over $MgSO_4$ and the solvent removed under reduced pressure. **12** can be recrystallized from MeCN however this results in very significant losses, possibly due to decomposition, 2D TLC also indicated that **12** has limited stability towards SiO_2. Therefore, in order to remove catalyst residues the material was dissolved in $CHCl_3$ and passed quickly through a short plug of SiO_2 (eluting $CHCl_3$) prior to removal of solvent to give **12** as a white solid (201 mg, ca. 90%) which was used without further purification.

^{1}H-NMR (400 MHz, $CDCl_3$): δ = 8.13 (s, 1H), 7.81 (d, J = 7.6 Hz, 1H), 7.77 (d, J = 7.6 Hz, 1H), 7.62 (td, J = 7.6, 1.2 Hz, 1H), 7.53 (td, J = 7.6, 1.0 Hz, 1H), 7.14 (s, 1H), 4.11 (t, J = 6.2 Hz, 2H), 1.86 (dt, J = 14.3, 6.2 Hz, 2H), 1.63 – 1.48 (m, 2H), 1.42 – 1.31 (m, 16H), 0.93 (t, J = 7.0 Hz, 3H)

^{13}C-NMR (101 MHz, $CDCl_3$): δ = 168.4, 139.3, 136.4, 133.6, 131.6, 130.9, 130.8, 128.9, 122.2, 121.7, 103.6, 84.2, 69.1, 31.8, 29.3, 25.8, 25.0, 22.8, 14.3

N.B. One aromatic carbon does not resolve, most likely the one bound to boron.[2]

^{11}B-NMR (128 MHz, $CDCl_3$): δ = 30.43

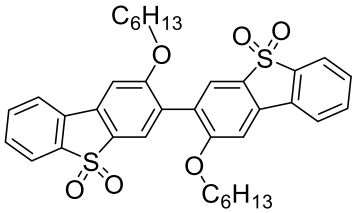

3,3'-Bi(2-(hexyloxy)dibenzothiophene-*S*,*S*-dioxide) (33DBS)

Boronic ester **12** (200 mg, 0.45 mmol), tetrabutylammonium fluoride trihydrate (170 mg, 0.54 mmol) and $PdCl_2(PPh_3)_2$ (10 mol%, 35 mg, 0.05 mmol) were dissolved in THF (8mL) and H_2O (2mL) in an open flask under air and stirred overnight. CH_2Cl_2 (20 mL) and H_2O (10 mL) were added to the reaction mixture and the aqueous layer was removed. The organic phase was dried over $MgSO_4$ and the solvent was removed under reduced pressure. The crude product was then redissolved in a small volume of CH_2Cl_2 and passed through a plug of SiO_2 (eluting CH_2Cl_2).

Purification was achieved by recrystallization from EtOH to give pure **33DBS** as colorless needles (97mg, 68%). mp. 198–199 °C

^{1}H-NMR (400 MHz, $CDCl_3$): δ = 7.84 (d, J = 7.6 Hz, 2H), 7.81 (d, J = 7.6 Hz, 2H), 7.74 (s, 2H), 7.65 (td, J = 7.6, 1.1 Hz, 2H), 7.55 (td, J = 7.6, 0.9 Hz, 2H), 7.27 (s, 2H), 4.09 (t, J = 6.5 Hz, 4H), 1.72 (dt, J = 13.1, 6.5 Hz, 4H), 1.35 (dt, J = 14.2, 7.0 Hz, 4H), 1.31 – 1.20 (m, 8H), 0.83 (t, J = 6.9 Hz, 6H)

^{13}C-NMR (101 MHz, $CDCl_3$): δ = 161.3, 139.1, 133.8, 131.6, 130.6, 129.2, 128.3, 125.3, 122.3, 121.5, 104.1, 69.3, 31.5, 29.0, 25.7, 22.6, 14.1

MS (ASAP): m/z = 631.2 $[M+H]^+$

HRMS (ASAP): m/z = calculated for $C_{36}H_{38}O_6S_2$ $[M]^+$: 630.2110; found: 630.2116

Elemental analysis: Found C, 68.30; H, 6.03; N, -0.03, Calculated C, 68.55; H, 6.07; N, 0.00

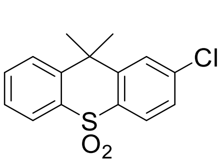

2-Chloro-9,9-dimethyl-9*H*-thioxanthene-*S*,*S*-dioxide (14)

Under an atmosphere of N_2, 2-chlorothioxanthone **13** (5.92 g, 24 mmol) was dissolved in toluene (100 mL) and the solution was cooled to 0 °C. $AlMe_3$ (2.0 M in toluene, 25 mL, 50

mmol) was added dropwise with stirring before the cooling bath was removed and the solution was allowed to warm to room temperature and left to stir overnight. The reaction mixture was then poured over a mixture of crushed ice (approximately 250 g) and conc. HCl (20 mL). The resulting precipitate was isolated by filtration over a ground glass sinter and washed with copious amounts of H_2O. The solid was then washed through the sinter with $CHCl_3$ (200 mL) and the resulting solution was dried over $MgSO_4$ prior to removal of the solvent under reduced pressure to yield the intermediate 2-chloro-9,9-dimethyl-9*H*-thioxanthene as an off-white solid. The crude intermediate was then re-dissolved in AcOH (60 mL) prior to addition of H_2O_2 (35% in water, 15 mL) with stirring and the reaction was heated to 125 °C for 48 h. After cooling to room temperature the reaction mixture was poured over ice and the resulting precipitate of crude **14** was isolated by filtration and washed with water then dried under a stream of air.

Purification was achieved by recrystallization from MeOH to give **14** as white needles (4.82 g) and a further crop of crystals (1.21 g) was obtained by further concentrating the mother liquor (6.03 g, 86% overall). mp. 139–140 °C

^{1}H-NMR (400 MHz, $CDCl_3$): δ = 8.18 (dd, J = 7.8, 1.3 Hz, 1H), 8.13 (d, J = 8.4 Hz, 1H), 7.74 (d, J = 8.2, 0.8 Hz, 1H), overlapping with 7.72 (d, J = 2.0 Hz, 1H), 7.61 (ddd, J = 8.1, 7.7, 1.6 Hz, 1H), 7.53 (dd, J = 7.7, 1.1 Hz, 1H), overlapping with 7.49 (dd, J = 8.4, 1.9 Hz, 1H), 1.88 (s, 6H)

^{13}C-NMR (101 MHz, $CDCl_3$): δ = 148.2, 145.5, 139.6, 136.9, 135.7, 133.4, 128.1, 128.0, 126.4, 126.2, 125.9, 124.6, 39.7, 31.0

MS (ASAP): m/z = 293.0 $[M+H]^+$

HRMS (ASAP): m/z = calculated for $C_{15}H_{14}ClSO_2$ $[M+H]^+$: 293.0403; found: 293.0410

Elemental analysis: Found C, 61.56; H, 4.48; N, -0.01, Calculated C, 61.54; H, 4.48; N, 0.00

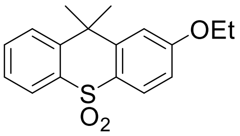

2-Ethoxy-9,9-dimethyl-9*H*-thioxanthene-*S*,*S*-dioxide (15)

Chloride **14** (5.29 g, 18.1 mmol) was dissolved in DMF (200 mL) and NaOEt (21 wt% in EtOH, 60 mL, 181 mmol) was added before the reaction was heated with stirring to 100 °C for 18 h. Upon cooling to room temperature the mixture was poured into H_2O (1000 mL) and the crude product was isolated by filtration as an off-white solid which was then recrystallized from a mixture of EtOH and hexane to give pure **15** as white needles (3.96 g, 72%). mp. 143–144 °C

^{1}H-NMR (400 MHz, $CDCl_3$): δ = 8.18 (dd, J = 7.7, 1.3 Hz, 1H), 8.12 (d, J = 8.7 Hz, 1H), 7.72 (dd, J = 8.1, 0.8 Hz, 1H), 7.57 (td, J = 7.7, 1.6 Hz, 1H), 7.49 (td, J = 7.6, 1.1 Hz, 1H), 7.22 (d, J = 2.4 Hz, 1H), 6.96 (dd, J = 8.7, 2.4 Hz, 1H), 4.12 (q, J = 7.0 Hz, 2H), 1.86 (s, 6H), 1.46 (t, J = 7.0 Hz, 3H)

^{13}C-NMR (101 MHz, $CDCl_3$): δ = 162.4, 148.3, 145.9, 137.4, 132.7, 128.8, 127.6, 126.8, 125.7, 124.2, 113.2, 111.9, 64.1, 39.5, 30.9, 14.8

MS (ASAP): m/z = 303.1 $[M+H]^+$

HRMS (ASAP): m/z = calculated for $C_{17}H_{19}O_3S$ $[M+H]^+$: 303.1055; found: 303.1059

Elemental analysis: Found C, 67.29; H, 6.04; N, -0.06, Calculated C, 67.52; H, 6.00; N, 0.00

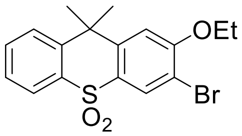

3-Bromo-2-ethoxy-9,9-dimethyl-9*H*-thioxanthene-*S*,*S*-dioxide (16)

NBS (356 mg, 2.00 mmol) was added in portions to a stirred solution of **15** in a mixture of trifluoroacetic acid (19 mL) and H_2SO_4 (1 mL) and the reaction was left to stir overnight at room temperature. The reaction mixture was then poured over ice and the resulting precipitate of crude **16** was isolated by filtration and washed with copious amounts of water.

Purification was achieved by column chromatography on SiO_2 (eluting 0-20% EtOAc in hexane). The first band to elute from the column was the 1-bromo isomer **17** followed by the desired product **16**. Recrystallization from MeOH gave **16** as colorless crystals (421 g, 55%). mp. 194–196 °C

^{1}H-NMR (400 MHz, $CDCl_3$): δ = 8.32 (s, 1H), 8.17 (dd, J = 7.8, 1.4 Hz, 1H), 7.72 (dd, J = 8.1, 0.8 Hz, 1H), 7.59 (td, J = 7.7, 1.6 Hz, 1H), 7.51 (td, J = 7.6, 1.1 Hz, 1H), 7.15 (s, 1H), 4.21 (q, J = 7.0 Hz, 2H), 1.87 (s, 6H), 1.53 (t, J = 7.0 Hz, 3H)

^{13}C-NMR (101 MHz, $CDCl_3$): δ = 158.7, 147.4, 145.5, 137.1, 132.9, 129.9, 129.5, 127.7, 125.7, 124.3, 111.4, 109.7, 65.4, 39.6, 30.9, 14.7

MS (ASAP): m/z = 381.0 $[M+H]^+$

HRMS (ASAP): m/z = calculated for $C_{17}H_{17}BrO_3S$ $[M]^+$:380.0082; found: 380.0072

Elemental analysis: Found C, 53.49; H, 4.50; N, -0.03, Calculated C, 53.55; H, 4.49; N, 0.00

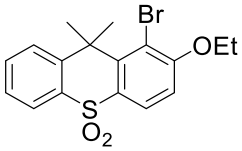

1-Bromo-2-ethoxy-9,9-dimethyl-9*H*-thioxanthene-*S*,*S*-dioxide (17)

Isolated as a minor product from the synthesis of **16**. **17** was recrystallized from MeOH as colorless crystals (123 mg, 16% from **15**) mp. 162–163 °C

^{1}H-NMR (400 MHz, $CDCl_3$): δ = 8.19 (d, J = 8.8 Hz, 1H), 8.08 (dd, J = 7.9, 1.2 Hz, 1H), 7.71 (dd, J = 8.3, 0.8 Hz, 1H), 7.64 (ddd, J = 8.4, 7.2, 1.5 Hz, 1H), 7.48 (ddd, J = 8.2, 7.2, 1.2 Hz, 1H), 7.06 (d, J = 8.8 Hz, 1H), 4.20 (q, J = 7.0 Hz, 2H), 2.16 (s, 6H), 1.54 (t, J = 7.0 Hz, 3H)

^{13}C-NMR (101 MHz, $CDCl_3$)): δ = 159.8, 147.8, 144.2, 133.1, 132.6, 129.3, 128.9, 127.5, 124.5, 122.3, 114.3, 111.9, 65.9, 40.9, 29.7, 14.7

MS (ASAP): m/z = 381.0 $[M+H]^+$

HRMS (ASAP): m/z = calculated for $C_{17}H_{18}BrO_3S$ $[M+H]^+$:381.0155; found: 381.0176

Elemental analysis: Found C, 53.73; H, 4.51; N, -0.06, Calculated C, 53.55; H, 4.49; N, 0.00

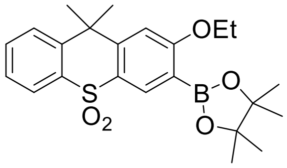

3-(4,4,5,5-Tetramethyl-1,3,2-dioxaborolan-2-yl)2-ethoxy-9,9-dimethyl-9*H*-thioxanthene-*S*,*S*-dioxide (18)

Under an atmosphere of N_2, **16** (200 mg, 0.52 mmol), was dissolved in anhydrous THF (5 mL) and the reaction mixture was cooled to -78 °C. *sec*-Butyl lithium (1.4 M in cyclohexane, 0.45 mL, 0.63 mmol) was added and the reaction was left to stir for 1 h during which time the solution turned yellow. Subsequently, 2-isopropoxy-4,4,5,5-tetramethyl-1,3,2-borolane (0.16 mL, 0.78 mmol) was added. After 15 min the cooling bath was removed and the reaction was allowed to warm to room temperature and stirred for 5 h. The reaction was quenched with H_2O (20 mL) and the crude product extracted with EtOAc (3 × 25 mL) and dried over $MgSO_4$ before the solvent was removed under reduced pressure to give crude **18** as an off-white solid (232 mg, 69 %). 2D TLC and NMR analysis indicated that the material has limited stability therefore it was used as obtained and without further purification.

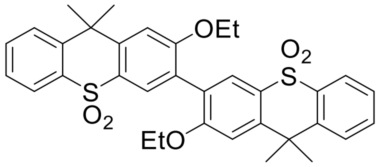

3,3'-Bi(2-ethoxy-9,9-dimethyl-9*H*-thioxanthene-*S*,*S*-dioxide) (33TXS)

Boronic ester **18** (232 mg, 0.55 mmol) bromide **16** (250 mg, 0.66 mmol) and K_2CO_3 (240 mg, 1.74 mmol) were combined in a mixture of dimethoxyethane (10 mL) and H_2O (2 mL) and the mixture was degassed thoroughly with N_2 for 30 minutes prior to addition of $Pd(PPh_3)_4$ (5 mol%, 34 mg, 0.03 mmol). The reaction was heated with stirring to 90 °C for 14 h. H_2O (20 mL) was added and the crude product extracted with EtOAc (3 × 25 mL) and dried over $MgSO_4$ before the solvent was removed under reduced pressure. The material was dissolved in CH_2Cl_2 and passed through a short column of SiO_2 (eluting CH_2Cl_2) then recrystallized from a mixture of hot EtOH and toluene to give colorless needles of **33TXS** (102 mg, 31%). mp. 272–273 °C

^{1}H-NMR (400 MHz, $CDCl_3$): δ = 8.19 (dd, J = 7.7, 1.5 Hz, 2H), 8.17 (s, 2H), 7.75 (dd, J = 8.1, 0.7 Hz, 2H), 7.59 (td, J = 7.7, 1.5 Hz, 2H), 7.50 (td, J = 7.6, 1.1 Hz, 2H), 7.23 (s, 2H), 4.16 (q, J = 7.0 Hz, 4H), 1.93 (s, 12H), 1.38 (t, J = 7.0 Hz, 6H)

^{13}C-NMR (101 MHz, $CDCl_3$): δ = 159.6, 147.8, 145.8, 137.6, 132.7, 128.4, 128.1, 127.6, 125.6, 125.0, 124.2, 108.8, 64.6, 39.6, 31.1, 14.7

MS (ASAP): m/z = 602.2 $[M]^+$, 603.2 $[M+H]^+$

HRMS (ASAP): m/z = calculated for $C_{34}H_{34}O_6S_2$ $[M+H]^+$:602.1797; found: 602.1783

Elemental analysis: Found C, 67.70; H, 5.69; N, 0.15, Calculated C, 67.75; H, 5.69; N, 0.00

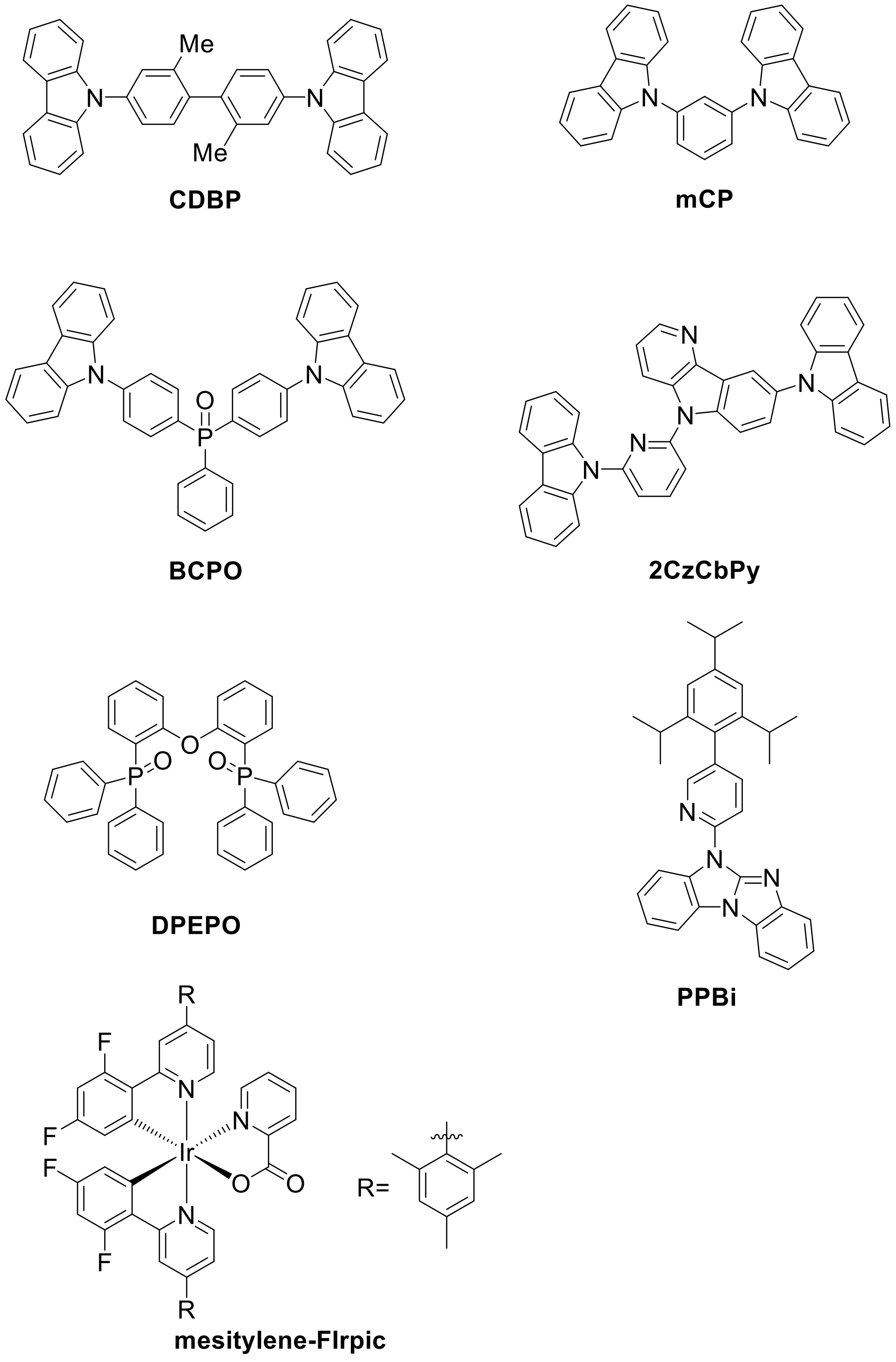

Figure S1 Structures of other hole transport and host materials mentioned in this work and the iridium phosphor mesitylene-FIrpic used in device studies.

Optoelectronic Methods

Solution state UV-Vis spectra were obtained using a Shimadzu UV-1800 UV−vis spectrophotometer. Solution state photoluminescence spectra were recorded on a SPEX Fluoromax luminescence spectrometer.

Films were prepared by drop casting from toluene solutions (with or without dissolved Zeonex) onto quartz or sapphire substrates preheated to 70 °C and allowed to rest until thoroughly dried (~3 minutes). Emission spectra of films were recorded on a Fluoromax 3 (Horiba Jobin Yvon) luminescence spectrometer, and absorption on a Shimadzu UV-3600. Phosphorescence spectra were collected using a gated CCD (Standford Computer Optics) triggered by a pulsed nitrogen laser (337 nm excitation). The sample was suspended in a stream of dry nitrogen at 80 K, and emission collected 80 ms after laser excitation for a collection duration of 15 ms.

Cyclic voltammetry was recorded using a Princeton Applied Research VersaSTAT 3. A glassy carbon disk, Pt wire, and Ag/Ag^+ ($AgNO_3$ in acetonitrile) were used as the working, counter, and reference electrodes, respectively. Measurements were corrected to the ferrocene/ferrocenium redox couple as an internal standard. Methylene chloride was used as the solvent with an analyte molarity of *ca.* 10^{-5} M in the presence of 10^{-1} M (n-Bu_4N)(PF_6) as a supporting electrolyte. Solutions were degassed with Ar and experiments run under a blanket of Ar.

Organic light-emitting diodes (OLEDs) were fabricated on patterned indium-tin-oxide (ITO) coated glass (VisionTek Systems) with a sheet resistance of 15 Ω/cm^2 using vacuum thermal evaporation. The substrates were sonicated for 15 minutes each in acetone and then isopropyl alcohol. After oxygen-plasma cleaning, the substrates were loaded into a Kurt J. Lesker Super Spectros 200 deposition chamber. All organic and cathode layers were thermally evaporated at a pressure below 10^{-7} mbar, at evaporation rates in the range of 0.1-0.5 Å/s. Characterization of the OLED devices was conducted in a 10-inch integrating sphere (Labsphere) coupled with a calibrated fibre spectrometer (Ocean OpticsUSB4000) and connected to a Keithley 2400 source measure unit. The **33Cz** hosted TADF OLED discussed in the main text had a pixel size of 2×4 mm, while the **33Cz** hosted PhOLED was 4×4 mm

X-Ray Crystallography

Single-crystal X-ray diffraction experiments were carried out on a Bruker D8 Venture 3-circle diffractometer using a PHOTON 100 CMOS area detector, Incoatec IμS microsources with focusing mirrors and a Cryostream 700 open-flow N_2 gas cryostat (Oxford Cryosystems). The structures were solved by direct methods using SHELXS 2013/1 program[3] and refined by full-matrix least squares using SHELXL 2018/3 software[4] on OLEX2 platform.[5] Crystal data and experimental details are listed in Table S1. In Fig S2-S6 C atoms are shown grey, N blue, O red, S yellow; H atoms are omitted.

Table S1. Crystal data and experimental details.

**Compound**	**33Cz**	**22Cz**	**33DBS**	**33TXS**	**10**
CCDC	1999181	1999182	1999183	1999184	1999185
Formula	$C_{30}H_{28}N_2O_2$	$C_{30}H_{28}N_2O_2$	$C_{36}H_{38}O_6S_2$	$C_{34}H_{34}O_6S_2$	$C_{18}H_{20}O_3S$
$D_{calc.}$/ g cm^{-3}	1.267	1.267	1.340	1.398	1.328
μ/mm^{-1}	0.08	0.62	1.92	0.23	0.22
Formula Weight	448.54	448.54	630.78	602.73	316.40
T/K	120	120	120	120	120
Crystal System	orthorhombic	monoclinic	monoclinic	orthorhombic	monoclinic
Space Group	$Pca2_1$ (no.29)	Cc (no.9)	$P2_1/c$ (no.14)	$Pca2_1$ (no.29)	$P2_1/n$ (no.14)
a/Å	9.7221(5)	5.0667(4)	15.0444(8)	13.6833(5)	7.3036(8)
b/Å	30.1218(15)	29.828(2)	13.8146(8)	8.3892(3)	15.6629(15)
c/Å	8.0288(4)	15.6083(12)	30.4129(16)	24.9540(10)	14.1952(15)
β/°	90	94.544(4)	98.378(3)	90	103.048(4)
V/Å^3	2351.2(2)	2351.5(3)	6253.3(6)	2864.5(2)	1581.9(3)
Z	4	4	8	4	4
Wavelength/Å	0.71073	1.54184	1.54184	0.71073	0.71073
Radiation type	Mo-K_α	Cu-K_α	Cu-K_α	Mo-K_α	Mo-K_α
$2\theta_{max}$/°	50	133.1	133.3	65	50
Reflections total	21309	8215	78336	69161	12238
unique	4133	3106	10838	10369	2796
with $I > 2\sigma(I)$	3747	2595	7407	9240	1953
R_{int}	0.048	0.055	0.119	0.041	0.071
Parameters, restraints	315, 1	312, 293	802, 0	392, 1	200, 0
$\Delta\rho_{min,max}$/eÅ^{-3}	0.21, -0.21	0.16, -0.17	0.30, -0.31	0.41, -0.33	0.27, -0.40
Goodness of fit	1.171	1.049	1.007	1.063	0.933
R_1, wR_2 (all data)	0.057, 0.106	0.060, 0.095	0.086, 0.100	0.044, 0.092	0.065, 0.091
R_1, wR_2 [$I > 2\sigma(I)$]	0.049, 0.104	0.043, 0.088	0.046, 0.087	0.035, 0.088	0.040, 0.083

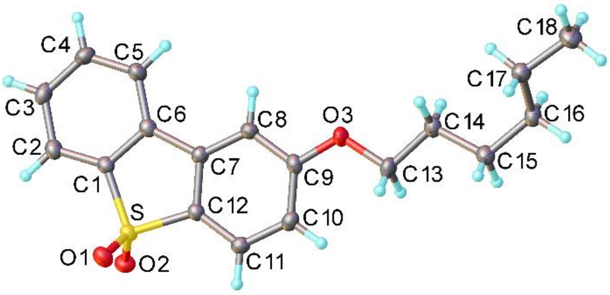

(a)

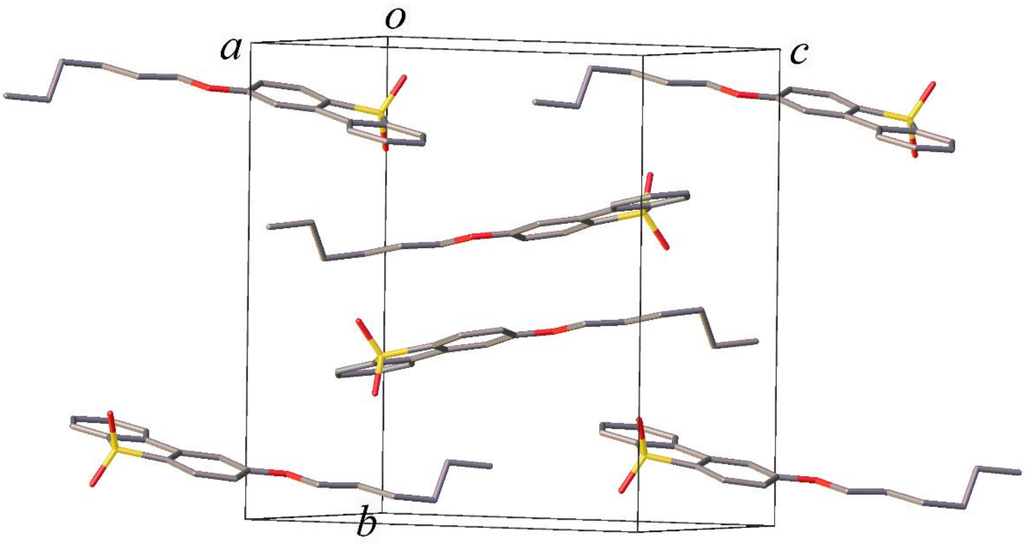

(b)

Figure S2 (a) Molecular structure of **10**; atomic displacement ellipsoids are drawn at the 50% probability level. **(b)** Molecular packing of **10**.

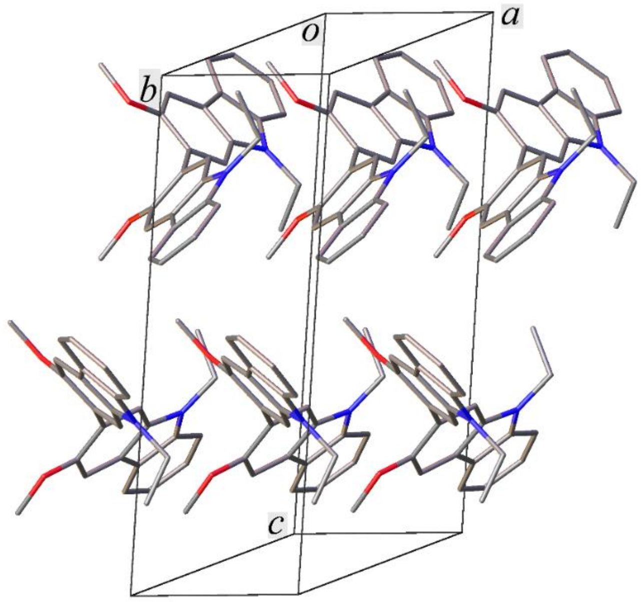

a)

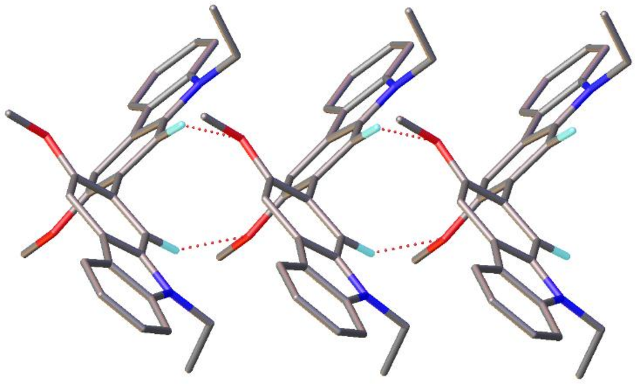

b)

Figure S3 (a) Crystallographically determined molecular packing for **22Cz**. **(b)** Strong pairwise interactions between adjacent **22Cz** molecules highlighted.

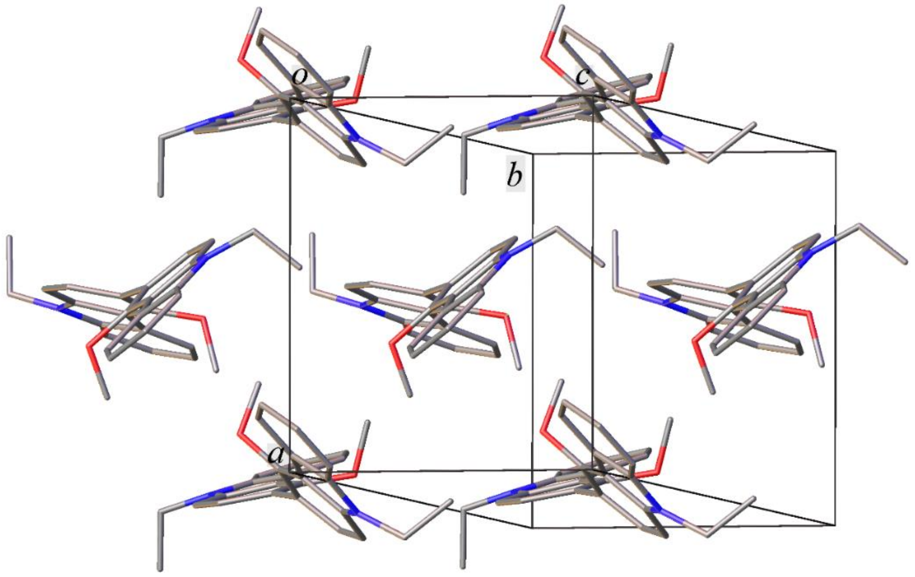

Figure S4 Crystallographically determined molecular packing for **33Cz**

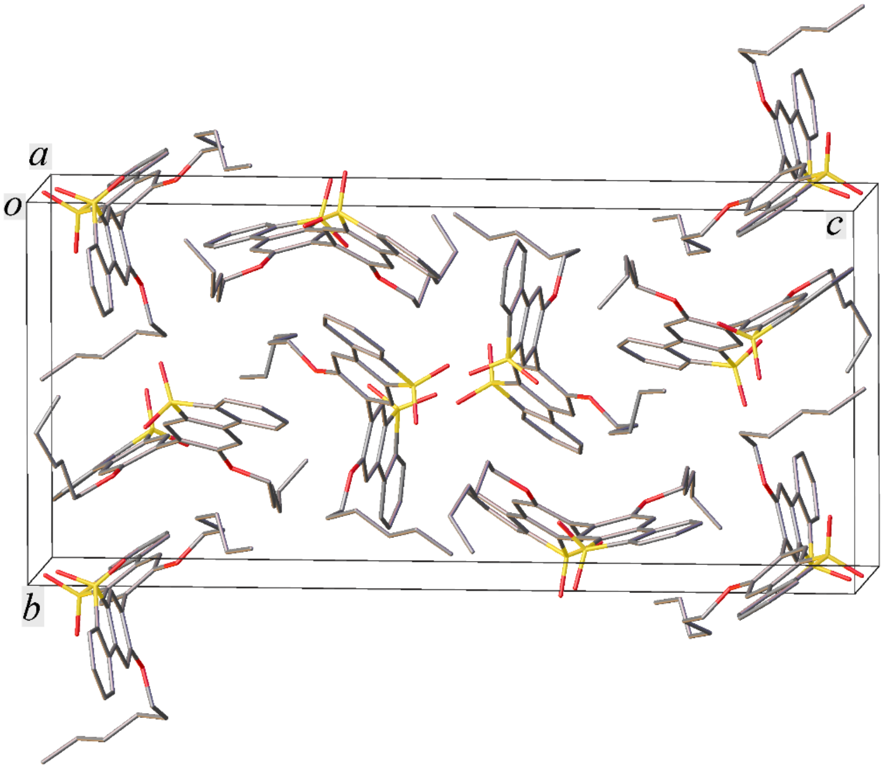

Figure S5 Crystallographically determined molecular packing for **33DBS**.

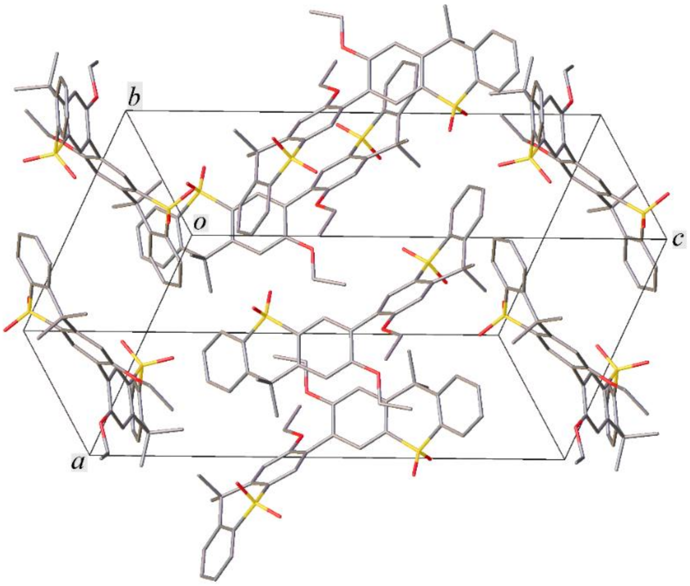

Figure S6 Crystallographically determined molecular packing for **33TXS**.

As shown in Table S2, the observed twist angles in the solid state tend to be wider than the calculated ones in isolated molecules, although the actual differences vary. The effect can be due to packing interactions. To investigate this, intermolecular interactions in the crystal structures were calculated by PIXEL approach[6] with the molecular electron density at the HF/3-21G approximation, using *CrystalExplorer 17* software.[7,8] The results are presented in Tables S3 and S4.

Table S2. Crystal packing effects

compound	τ, °		m.p., °C	D_x, g/cm^3 at 120 K	E_{pk}, kJ/mol	E_{max}, kJ/mol
	X-ray	calcd.				
**22Cz**	81.3	53.4	242 – 244	1.267	−429	−122
**33Cz**	57.5	47.3	206 – 208	1.267	−404	−71
**33DBS**	70.2, 50.6	50.9	198 – 199	1.340		−124
**33TXS**	59.0	47.4	272 – 273	1.398	−516	−119

Isomers **22Cz** and **33Cz** have the same density in the solid state but the former has, in accordance with its higher m.p., much higher total packing energy (E_{pk}). This is primarily due to the strong (E_{tot} =−122 kJ/mol) pairwise interactions shown in Fig. S3b, comprising both a large dispersion contribution due to parallel (though not π-π stacked!) arrangement of the carbazole units and a large electrostatic contribution due mainly to the relatively short (H…O 2.34 – 2.39 Å) intermolecular C-H…O hydrogen bonds. (Note that the only parts of the molecular surface with substantially negative electrostatic potential are the oxygen atoms.) These features can coexist only if the molecule is twisted almost perpendicularly, thus the τ angle is 28° wider than the isolated-molecule minimum. No such pattern exists, or indeed can be sterically favourable, in the isomer **33Cz**. In this structure, each molecule has six others lying alongside it, however, each pairwise interaction is weaker than in **22Cz**, E_{tot} ranging from −41 to −71 kJ/mol. In this case, the twist angle is also wider than predicted, but only by 10°.

Crystal structures of **33DBS** and **33TXS** do not allow such straightforward comparisons, both because of different composition and because the packing is strongly influenced by polar SO_2 groups, Coulombic interactions decaying slowly with distance. Nevertheless, two symmetrically independent molecules of **33DBS** drastically reveal the role of packing effects, one of them having practically the predicted twist angle and the other, nearly 20° wider.

Table S3. Interaction energies (kJ/mol) between molecules in the crystal of **22Cz:** electrostatic, polarisation, dispersion, repulsive and total

	N	E_ele	E_pol	E_dis	E_rep	E_tot
	2	-6.3	-2.7	-35.5	14.4	-28.5
	2	-43.6	-19.6	-138.2	73.1	-122.4
	2	-7.7	-3.3	-34.3	20.5	-24.3
	2	0.0	-0.4	-6.2	2.1	-4.1
	2	-2.3	-1.6	-20.3	10.0	-13.6
	2	-2.9	-1.6	-20.4	11.1	-13.4
	2	-2.0	-0.4	-9.2	4.9	-6.6
	2	0.3	-0.1	-2.4	0.1	-1.8

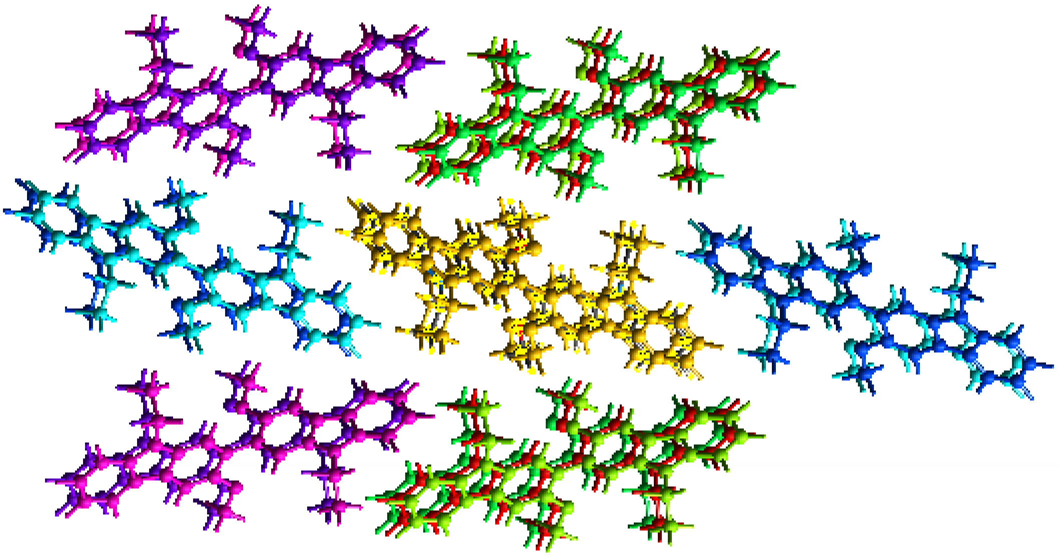

Reference molecule is highlighted, surrounding molecules indicated by colour in the table.

Table S4. Interaction energies (kJ/mol) between molecules in the crystal of **33Cz:** electrostatic, polarisation, dispersion, repulsive and total

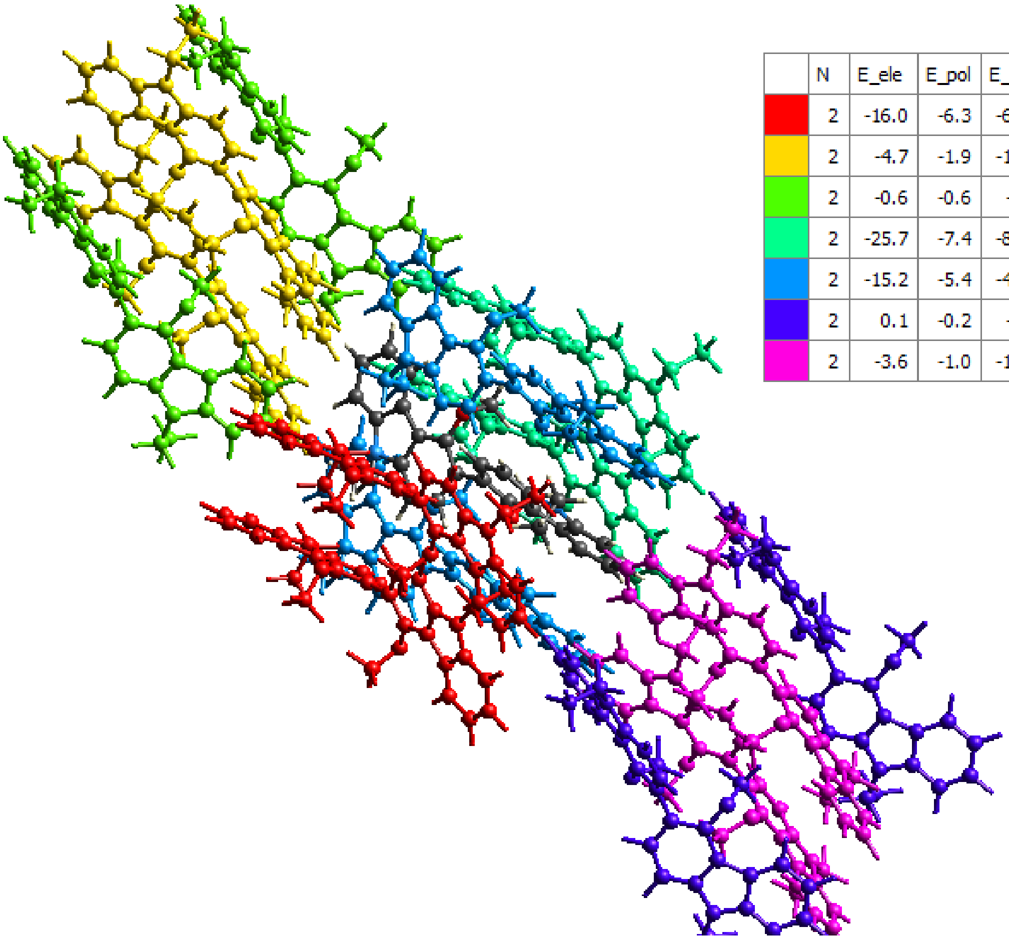

	N	E_ele	E_pol	E_dis	E_rep	E_tot
	2	-16.0	-6.3	-68.2	33.1	-55.0
	2	-4.7	-1.9	-19.0	12.8	-12.7
	2	-0.6	-0.6	-7.2	0.5	-7.1
	2	-25.7	-7.4	-89.2	50.1	-70.8
	2	-15.2	-5.4	-41.2	18.3	-41.3
	2	0.1	-0.2	-3.7	0.0	-3.4
	2	-3.6	-1.0	-16.1	8.6	-11.9

Reference molecule is shown black, surrounding molecules indicated by colour in the table.

Computational Results

All calculations were carried out using ORCA v4.0.1.2[9,10] and molecular orbital diagrams were generated using Avogadro v1.2.0[11] and POV-Ray. Optimised geometries were determined using B3LYP with the 6–31G** basis set.[12–14] All S_0 geometries were true minima based on no imaginary frequencies found. Electronic structures and TDDFT calculations were also carried out using the optimised geometries.

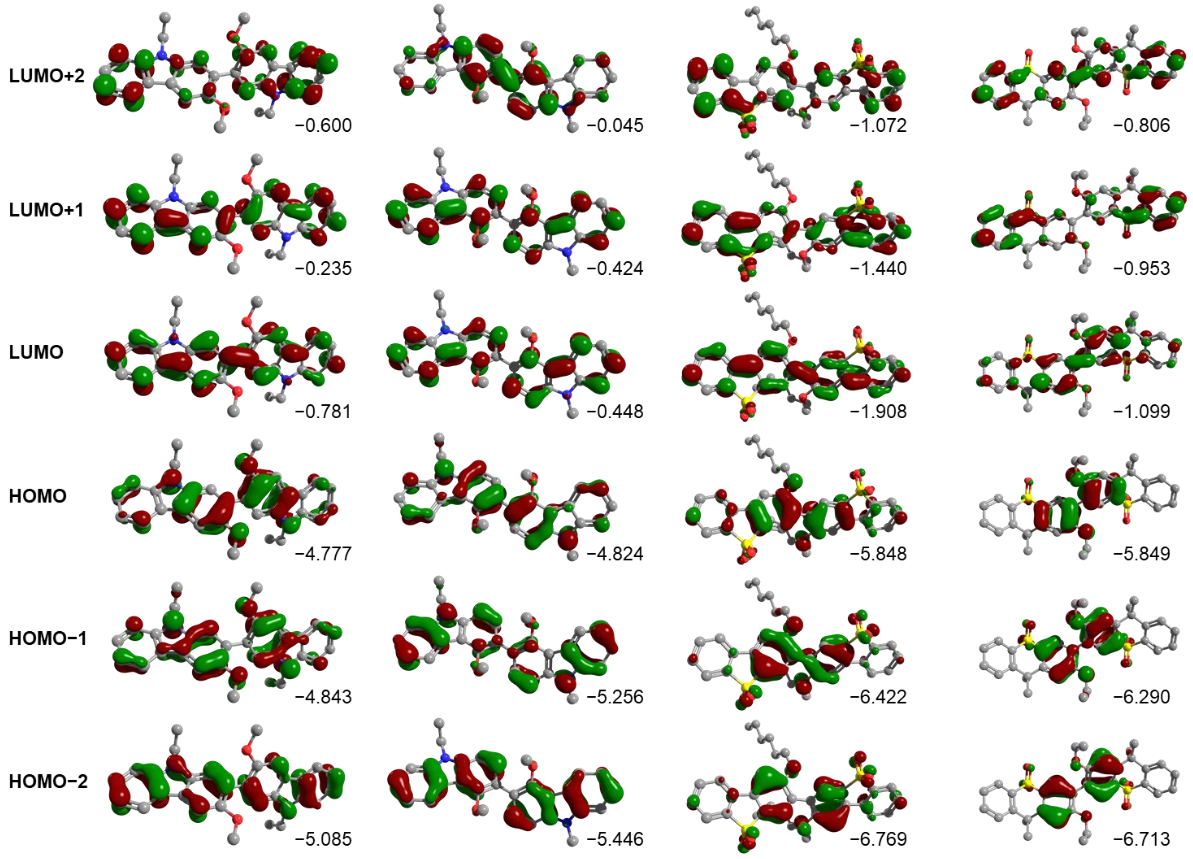

Figure S7 Frontier molecular orbital wavefunctions and energies for **22Cz**, **33Cz**, **33DBS** and **33TXS**.

Table S5. Transitions to the first 15 singlet and first 15 triplet states calculated for the optimised ground state geometry of **22Cz** at TD-B3LYP/def2-SVP.

No.	Transition	Energy (cm^{-1})	Wavelength (nm)	*f*	Orbital contribution
1	$S_0 \rightarrow T_1$	23229	431	0	HOMO→LUMO (79%)
2	$S_0 \rightarrow T_2$	24032	416	0	H-1→LUMO (76%), HOMO→L+2 (19%)
3	$S_0 \rightarrow T_3$	25212	397	0	H-2→LUMO (71%), H-1→L+1 (14%)
4	$S_0 \rightarrow T_4$	27013	370	0	H-3→LUMO (35%), H-2→L+1 (34%), HOMO→L+1 (13%)
5	$S_0 \rightarrow S_1$	28582	350	0.304	HOMO→LUMO (94%)
6	$S_0 \rightarrow S_2$	28928	346	0.013	H-1→LUMO (95%)
7	$S_0 \rightarrow T_5$	30860	324	0	H-1→LUMO (15%), HOMO→L+1 (56%)
8	$S_0 \rightarrow T_6$	30944	323	0	H-5→LUMO (11%), H-1→L+1 (42%), HOMO→LUMO (10%)
9	$S_0 \rightarrow S_3$	31236	320	0.625	H-2→LUMO (84%)
10	$S_0 \rightarrow T_7$	31412	318	0	H-4→LUMO (13%), H-1→L+2 (30%), HOMO→L+3 (23%)
11	$S_0 \rightarrow T_8$	31471	318	0	H-2→LUMO (11%), H-1→L+1 (23%), H-1→L+3 (22%), HOMO→L+2 (17%)
12	$S_0 \rightarrow S_4$	32460	308	0.001	HOMO→L+1 (86%)
13	$S_0 \rightarrow S_5$	33040	303	0.137	H-1→L+1 (87%)
14	$S_0 \rightarrow T_9$	34262	292	0	H-6→LUMO (14%), H-3→L+1 (18%), H-2→L+6 (10%), HOMO→L+4 (16%)
15	$S_0 \rightarrow T_{10}$	34450	290	0	H-3→LUMO (35%), H-2→L+1 (24%)
16	$S_0 \rightarrow T_{11}$	34868	287	0	H-3→LUMO (12%), H-2→L+1 (19%), H-2→L+3 (17%), H-1→L+4 (12%)
17	$S_0 \rightarrow T_{12}$	34925	286	0	H-3→L+1 (18%), H-3→L+3 (11%), H-2→L+2 (32%)
18	$S_0 \rightarrow S_6$	35152	285	0.004	H-3→LUMO (11%), H-2→L+1 (87%)
19	$S_0 \rightarrow T_{13}$	35467	282	0	H-5→LUMO (30%), H-4→LUMO (11%), H-4→L+1 (11%),
20	$S_0 \rightarrow T_{14}$	35495	282	0	H-5→LUMO (11%), H-5→L+1 (12%), H-4→LUMO (30%), H-1→L+2 (10%)
21	$S_0 \rightarrow S_7$	35723	280	0.002	H-3→LUMO (57%), H-1→L+2 (14%), HOMO→L+3 (14%)
22	$S_0 \rightarrow T_{15}$	36137	277	0	H-2→L+3 (21%), HOMO→L+3 (22%)
23	$S_0 \rightarrow S_8$	38214	262	0.163	H-3→L+1 (39%), H-1→L+3 (14%), HOMO→L+2 (36%)
24	$S_0 \rightarrow S_9$	39793	251	0.014	HOMO→L+3 (56%)
25	$S_0 \rightarrow S_{10}$	39829	251	0.056	H-5→LUMO (23%), HOMO→L+2 (35%), HOMO→L+3 (11%)
26	$S_0 \rightarrow S_{11}$	40246	249	0.001	H-4→LUMO (54%), H-1→L+2 (32%)
27	$S_0 \rightarrow S_{12}$	40433	247	0.175	H-5→LUMO (21%), H-1→L+3 (62%)
28	$S_0 \rightarrow S_{13}$	41166	243	0.489	H-5→LUMO (29%), H-3→L+1 (33%), HOMO→L+4 (10%)
29	$S_0 \rightarrow S_{14}$	41311	242	0.011	H-4→LUMO (19%), H-2→L+3 (20%), H-1→L+2 (33%)
30	$S_0 \rightarrow S_{15}$	42046	238	0.389	H-2→L+2 (63%)

Table S6. Transitions to the first 15 singlet and first 15 triplet states calculated for the optimised ground state geometry of **33Cz** at TD-B3LYP/def2-SVP.

No.	Transition	Energy (cm^{-1})	Wavelength (nm)	*f*	Orbital contribution
1	$S_0 \rightarrow T_1$	26405	379	0	H-2→L+1 (11%), H-1→LUMO (23%), HOMO→L+1 (36%), HOMO→L+2 (19%)
2	$S_0 \rightarrow T_2$	26563	377	0	H-1→L+1 (14%), HOMO→LUMO (78%)
3	$S_0 \rightarrow T_3$	26916	372	0	H-3→LUMO (22%), H-2→L+1 (12%), HOMO→L+1 (40%), HOMO→L+2 (16%)
4	$S_0 \rightarrow T_4$	27495	364	0	H-3→L+1 (24%), H-2→LUMO (40%), H-1→L+1 (20%)
5	$S_0 \rightarrow T_5$	29429	340	0	H-2→L+1 (19%), H-1→LUMO (18%), HOMO→L+2 (50%)
6	$S_0 \rightarrow S_1$	30842	324	0.008	HOMO→LUMO (89%)
7	$S_0 \rightarrow S_2$	31148	321	0.041	HOMO→L+1 (87%)
8	$S_0 \rightarrow T_6$	31800	315	0	H-1→L+1 (13%), H-1→L+2 (31%), HOMO→L+3 (21%)
9	$S_0 \rightarrow T_7$	32576	307	0	H-4→L+1 (12%), H-3→LUMO (10%), H-2→L+2 (10%), H-1→LUMO (20%), HOMO→L+1 (10%), HOMO→L+4 (13%)
10	$S_0 \rightarrow T_8$	33675	297	0	H-3→L+1 (18%), H-1→L+1 (24%), H-1→L+2 (25%), HOMO→LUMO (12%)
11	$S_0 \rightarrow S_3$	34164	293	0.670	H-1→LUMO (39%), HOMO→L+2 (52%)
12	$S_0 \rightarrow T_9$	34330	291	0	H-3→LUMO (14%), H-2→L+2 (29%), H-1→LUMO (14%), H-1→L+3 (16%)
13	$S_0 \rightarrow S_4$	34756	288	0.001	H-1→L+1 (83%)
14	$S_0 \rightarrow T_{10}$	34826	287	0	H-3→L+2 (56%), H-1→L+2 (10%)
15	$S_0 \rightarrow S_5$	35061	285	0.480	H-1→LUMO (47%), HOMO→L+2 (31%)
16	$S_0 \rightarrow T_{11}$	35146	285	0	H-1→L+4 (21%), HOMO→L+3 (12%), HOMO→124 (14%)
17	$S_0 \rightarrow T_{12}$	35188	284	0	H-3→L+3 (11%), H-2→L+2 (41%), HOMO→L+4 (18%)
18	$S_0 \rightarrow T_{13}$	35999	278	0	H-5→LUMO (11%), H-4→L+1 (12%), H-2→L+1 (31%), H-1→LUMO (15%)
19	$S_0 \rightarrow T_{14}$	36094	277	0	H-3→L+1 (11%), H-2→LUMO (35%), H-1→L+1 (21%)
20	$S_0 \rightarrow S_6$	36352	275	0.0001	H-2→LUMO (64%), H-1→L+2 (23%)
21	$S_0 \rightarrow S_7$	36940	271	0.180	H-3→LUMO (32%), H-2→L+1 (61%)
22	$S_0 \rightarrow S_8$	37209	269	0.001	H-2→LUMO (11%), H-1→L+2 (22%)
23	$S_0 \rightarrow T_{15}$	37372	268	0	H-5→L+1 (10%), H-3→L+1 (12%)
24	$S_0 \rightarrow S_9$	38328	261	1.508	H-3→LUMO (46%), H-2→L+1 (16%), H-2→L+2 (11%), HOMO→L+2 (11%)
25	$S_0 \rightarrow S_{10}$	38671	259	0.004	H-3→L+1 (11%), H-1→L+2 (26%), HOMO→L+3 (54%)
26	$S_0 \rightarrow S_{11}$	39946	250	0.072	H-2→L+2 (51%), HOMO→L+4 (28%)
27	$S_0 \rightarrow S_{12}$	40877	245	0.004	H-4→LUMO (14%), H-3→L+2 (59%)
28	$S_0 \rightarrow S_{13}$	41928	239	0.163	H-5→LUMO (11%), H-4→L+1 (25%), H-1→L+3 (10%), HOMO→L+4 (33%)
29	$S_0 \rightarrow S_{14}$	42231	237	0.047	H-4→LUMO (30%), H-3→L+2 (10%), H-1→L+2 (10%), HOMO→L+3 (17%)
30	$S_0 \rightarrow S_{15}$	42858	233	0.015	H-4→LUMO (29%), H-2→LUMO (11%), HOMO→L+7 (11%)

Table S7. Transitions to the first 15 singlet and first 15 triplet states calculated for the optimised ground state geometry of **33DBS** at TD-B3LYP/def2-SVP.

**No.**	**Transition**	**Energy (cm^{-1})**	**Wavelength (nm)**	***f***	**Orbital contribution**
1	$S_0 \rightarrow T_1$	22180	451	0	HOMO→LUMO (85%)
2	$S_0 \rightarrow T_2$	24865	402	0	H-3→LUMO (10%), H-1→LUMO (29%), HOMO→L+1 (50%)
3	$S_0 \rightarrow S_1$	28487	351	0.702	HOMO→LUMO (96%)
4	$S_0 \rightarrow T_3$	29211	342	0	H-3→LUMO (32%), H-2→L+1 (13%), H-1→LUMO (42%)
5	$S_0 \rightarrow T_4$	29607	338	0	H-3→L+1 (16%), H-2→LUMO (66%)
6	$S_0 \rightarrow T_5$	29888	335	0	HOMO→L+2 (66%)
7	$S_0 \rightarrow T_6$	30332	330	0	H-1→L+2 (13%), HOMO→L+3 (57%)
8	$S_0 \rightarrow S_2$	31315	319	0.001	H-1→LUMO (42%), HOMO→L+1 (51%)
9	$S_0 \rightarrow T_7$	32092	312	0	H-3→LUMO (15%), H-1→LUMO (15%), HOMO→L+1 (28%), HOMO→L+3 (11%)
10	$S_0 \rightarrow T_8$	32128	311	0	H-4→LUMO (11%)
11	$S_0 \rightarrow S_3$	32601	307	0.006	H-1→LUMO (46%), HOMO→L+1 (42%)
12	$S_0 \rightarrow T_9$	32709	306	0	H-5→LUMO (13%), H-3→L+2 (13%), H-2→L+3 (15%)
13	$S_0 \rightarrow S_4$	33010	303	0.002	H-2→LUMO (22%), HOMO→L+2 (66%)
14	$S_0 \rightarrow T_{10}$	33173	302	0	H-1→L+1 (38%), HOMO→L+2 (11%)
15	$S_0 \rightarrow S_5$	34089	293	0.001	H-3→LUMO (13%), HOMO→L+3 (68%)
16	$S_0 \rightarrow T_{11}$	34342	291	0	H-5→L+1 (18%), H-4→LUMO (34%)
17	$S_0 \rightarrow T_{12}$	34356	291	0	H-5→LUMO (12%), H-5→L+2 (13%), H-4→L+1 (14%)
18	$S_0 \rightarrow T_{13}$	34721	288	0	H-5→LUMO (24%), H-5→L+2 (11%), H-4→L+3 (15%)
19	$S_0 \rightarrow T_{14}$	34901	287	0	H-5→L+3 (18%), H-4→L+2 (18%), H-1→L+1 (25%)
20	$S_0 \rightarrow S_6$	35625	281	0.327	H-2→LUMO (57%), HOMO→L+2 (17%)
21	$S_0 \rightarrow S_7$	35823	279	0.002	H-1→L+1 (91%)
22	$S_0 \rightarrow T_{15}$	36042	278	0	H-4→LUMO (13%), HOMO→L+4 (23%), HOMO→L+5 (12%)
23	$S_0 \rightarrow S_8$	36365	275	0.012	H-5→LUMO (16%), H-3→LUMO (59%)
24	$S_0 \rightarrow S_9$	37647	266	0.117	H-4→LUMO (39%), H-1→L+3 (15%), HOMO→L+2 (11%)
25	$S_0 \rightarrow S_{10}$	37725	265	0.008	H-5→LUMO (17%), H-1→L+2 (42%), HOMO→L+3 (10%)
26	$S_0 \rightarrow S_{11}$	38386	261	0.0001	H-7→L+1 (13%), H-6→LUMO (75%)
27	$S_0 \rightarrow S_{12}$	38559	259	0.001	H-7→LUMO (67%), H-6→L+1 (18%)
28	$S_0 \rightarrow S_{13}$	38661	259	0.001	H-2→L+1 (60%), H-1→L+2 (10%)
29	$S_0 \rightarrow S_{14}$	39221	255	0.180	H-4→LUMO (12%), H-3→L+1 (14%), H-1→L+3 (57%)
30	$S_0 \rightarrow S_{15}$	39971	250	0.015	H-5→LUMO (17%), H-1→L+2 (18%), HOMO→L+4 (12%)

Table S8. Transitions to the first 15 singlet and first 15 triplet states calculated for the optimised ground state geometry of **33TXS** at TD-B3LYP/def2-SVP.

No.	Transition	Energy (cm^{-1})	Wavelength (nm)	*f*	Orbital contribution
**1**	$S_0 \rightarrow T_1$	26950	371	0	HOMO→LUMO (70%)
**2**	$S_0 \rightarrow T_2$	29998	333	0	H-1→LUMO (27%), HOMO→L+1 (37%)
**3**	$S_0 \rightarrow T_3$	31626	316	0	H-3→L+1 (12%), HOMO→L+2 (28%)
4	$S_0 \rightarrow T_4$	32065	312	0	H-6→L+3 (10%), H-4→L+1 (21%), H-3→LUMO (10%), H-3→L+2 (10%)
5	$S_0 \rightarrow T_5$	32530	307	0	H-3→L+1 (10%), HOMO→L+2 (23%), HOMO→L+6 (19%)
6	$S_0 \rightarrow T_6$	33223	301	0	H-1→LUMO (32%), H-1→L+2 (27%), HOMO→L+5 (10%)
7	$S_0 \rightarrow T_7$	34089	293	0	HOMO→LUMO (10%)
8	$S_0 \rightarrow T_8$	34665	289	0	H-2→L+5 (11%), HOMO→L+5 (10%), HOMO→L+7 (12%)
9	$S_0 \rightarrow S_1$	34898	297	0.294	HOMO→LUMO (79%), HOMO→L+2 (11%)
10	$S_0 \rightarrow S_2$	35014	286	0.001	H-1→LUMO (11%), HOMO→L+1 (80%)
11	$S_0 \rightarrow T_9$	35604	281	0	H-2→L+1 (12%), H-1→LUMO (17%), HOMO→L+1 (26%)
12	$S_0 \rightarrow S_3$	35624	281	0.086	HOMO→LUMO (14%), HOMO→L+2 (76%)
13	$S_0 \rightarrow T_{10}$	35771	280	0	H-2→LUMO (29%), H-1→L+1 (23%), HOMO→L+2 (20%)
14	$S_0 \rightarrow T_{11}$	36147	277	0	H-6→L+1 (36%), H-5→LUMO (13%), H-5→L+2 (21%)
15	$S_0 \rightarrow T_{12}$	36158	277	0	H-6→LUMO (13%), H-6→L+2 (21%), H-5→L+1 (35%)
16	$S_0 \rightarrow S_4$	36867	271	0.007	H-1→LUMO (63%), HOMO→L+1 (15%), HOMO→L+5 (10%)
17	$S_0 \rightarrow T_{13}$	37029	270	0	H-6→L+4 (12%), H-5→L+3 (20%), H-5→L+4 (14%)
18	$S_0 \rightarrow T_{14}$	37030	270	0	H-6→L+3 (20%), H-6→L+4 (13%), H-5→L+4 (12%)
19	$S_0 \rightarrow T_{15}$	38182	262	0	HOMO→L+1 (14%), HOMO→L+3 (11%), HOMO→L+5 (25%)
20	$S_0 \rightarrow S_5$	38525	260	0.225	H-1→L+1 (84%)
21	$S_0 \rightarrow S_6$	39358	254	0.003	H-1→L+2 (10%), HOMO→L+3 (78%)
22	$S_0 \rightarrow S_7$	39572	253	0.0001	H-1→L+2 (76%)
23	$S_0 \rightarrow S_8$	39900	251	0.038	H-2→LUMO (10%), HOMO→L+4 (69%)
24	$S_0 \rightarrow S_9$	40152	249	0.195	H-2→LUMO (44%), HOMO→L+4 (13%), HOMO→L+6 (11%)
25	$S_0 \rightarrow S_{10}$	41070	244	0.003	HOMO→L+5 (43%), HOMO→L+7 (12%)
26	$S_0 \rightarrow S_{11}$	41762	240	0.015	H-6→LUMO (19%), H-5→L+1 (20%), H-3→L+3 (12%)
27	$S_0 \rightarrow S_{12}$	41798	239	0.001	H-6→L+1 (16%), H-5→LUMO (16%), H-4→L+3 (10%), HOMO→L+5 (14%)
28	$S_0 \rightarrow S_{13}$	42121	237	0.014	H-4→LUMO (10%), H-2→L+1 (51%)
29	$S_0 \rightarrow S_{14}$	42129	237	0.050	H-4→LUMO (18%), H-2→L+1 (14%), HOMO→L+6 (17%)
30	$S_0 \rightarrow S_{15}$	42300	236	0.008	H-3→LUMO (52%)

Table S9 Oscillator strengths and dihedral angles for the S_0, S_1 and S_3 states of **22Cz** and **33Cz** and the S_0 and S_1 states of **33DBS** and **33TXS**.

Dimer	Singlet State	*f*	τ (°)
**22Cz**	S_3	0.625	37
	S_1	0.304	39
	S_0	-	53
**33Cz**	S_3	0.670	29
	S_1	0.008	37
	S_0	-	47
**33DBS**	S_1	0.702	36
	S_0	-	51
**33TXS**	S_1	0.294	34
	S_0	-	47

Figure S8 Variation of E_g, E_{S1} and E_{T1} with bridging dihedral angle for **33Cz**.

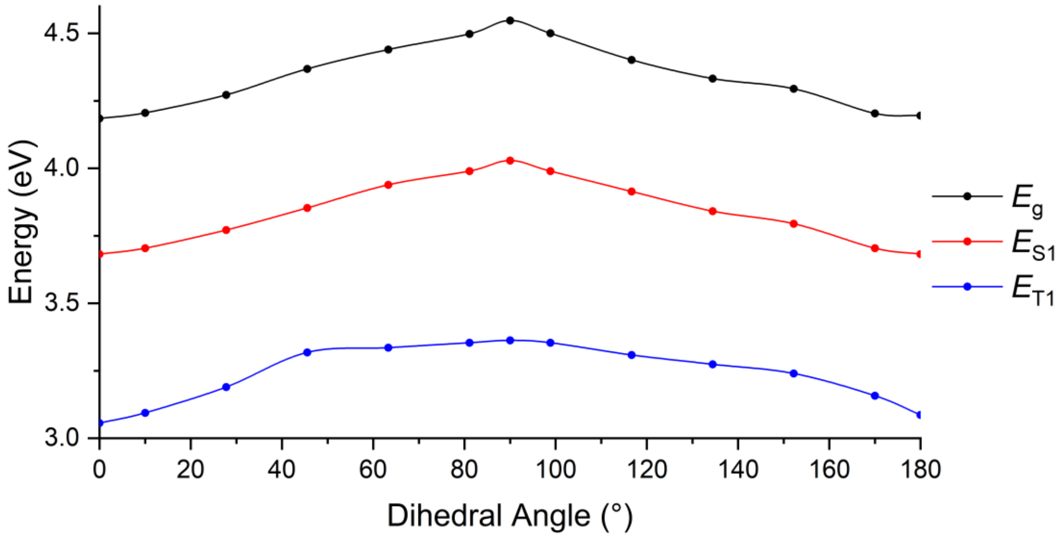

Figure S9 Phosphorescence spectra from neat films of other host materials (structures shown in Figure S1). The sample was suspended in a stream of dry nitrogen at 80 K, and emission collected 80 ms after laser excitation for a collection duration of 15 ms.

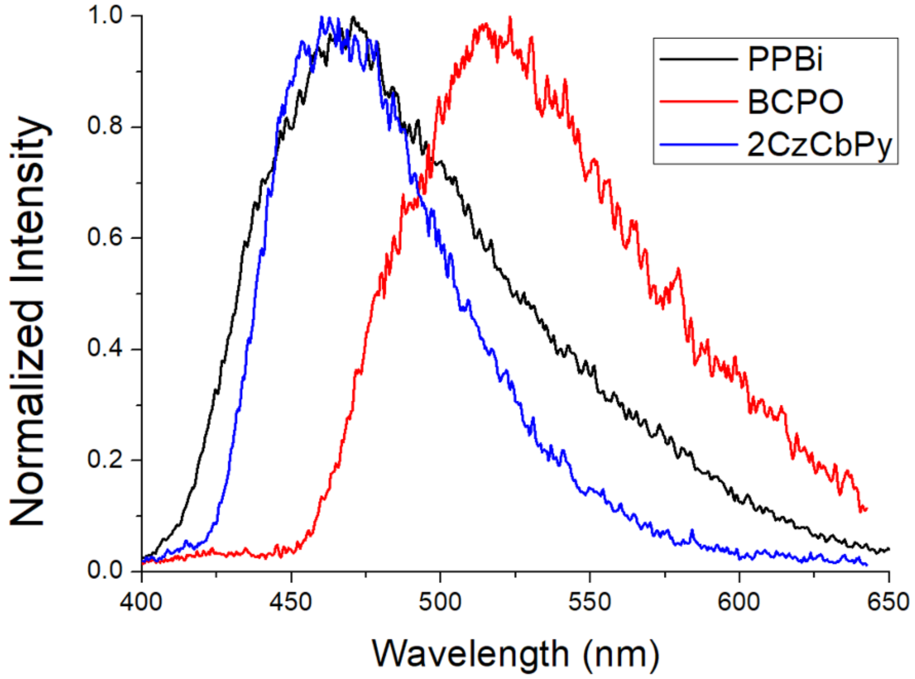

Copies of NMR Spectra:

^{1}H NMR of **8**

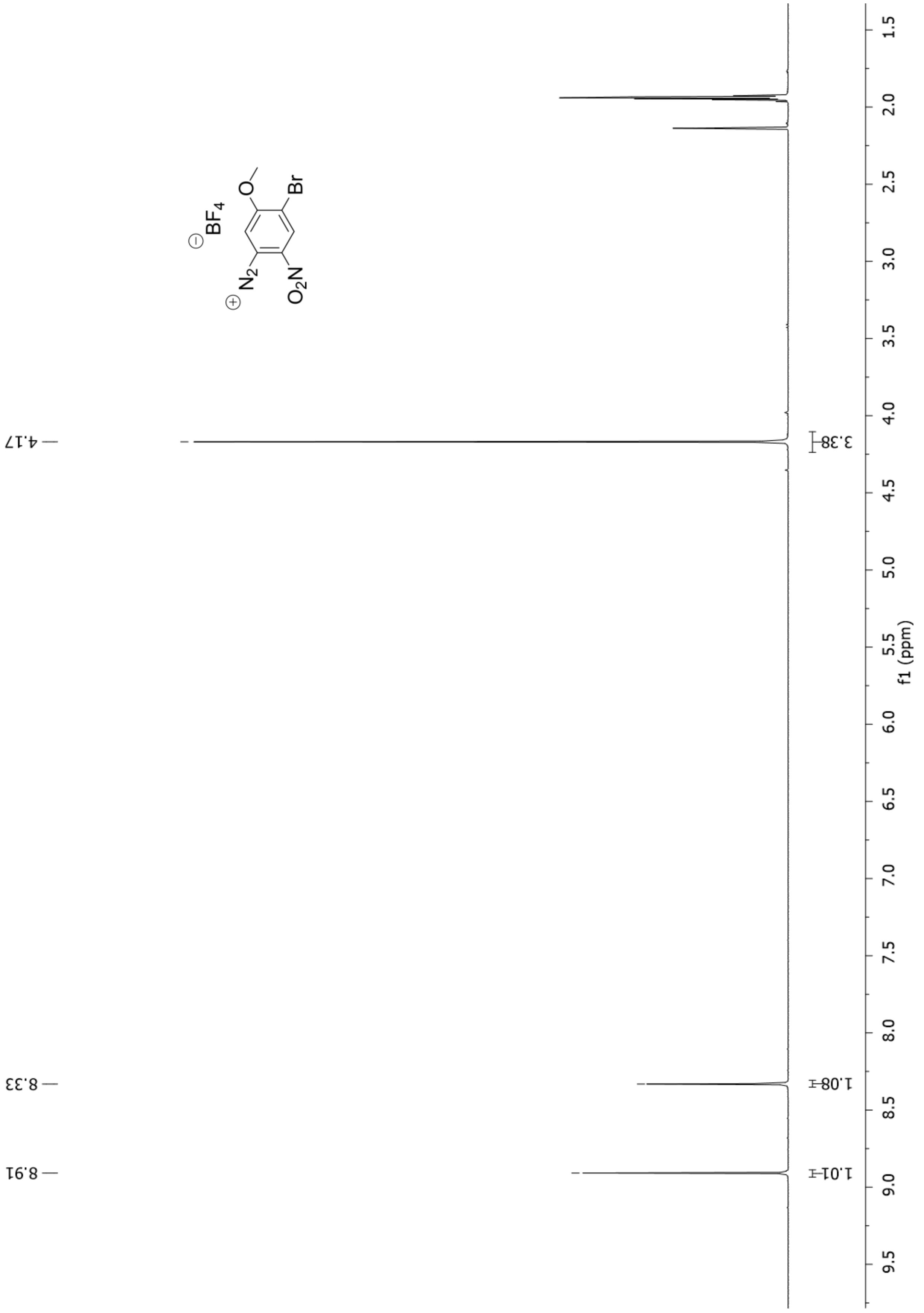

^{19}F NMR of **8**

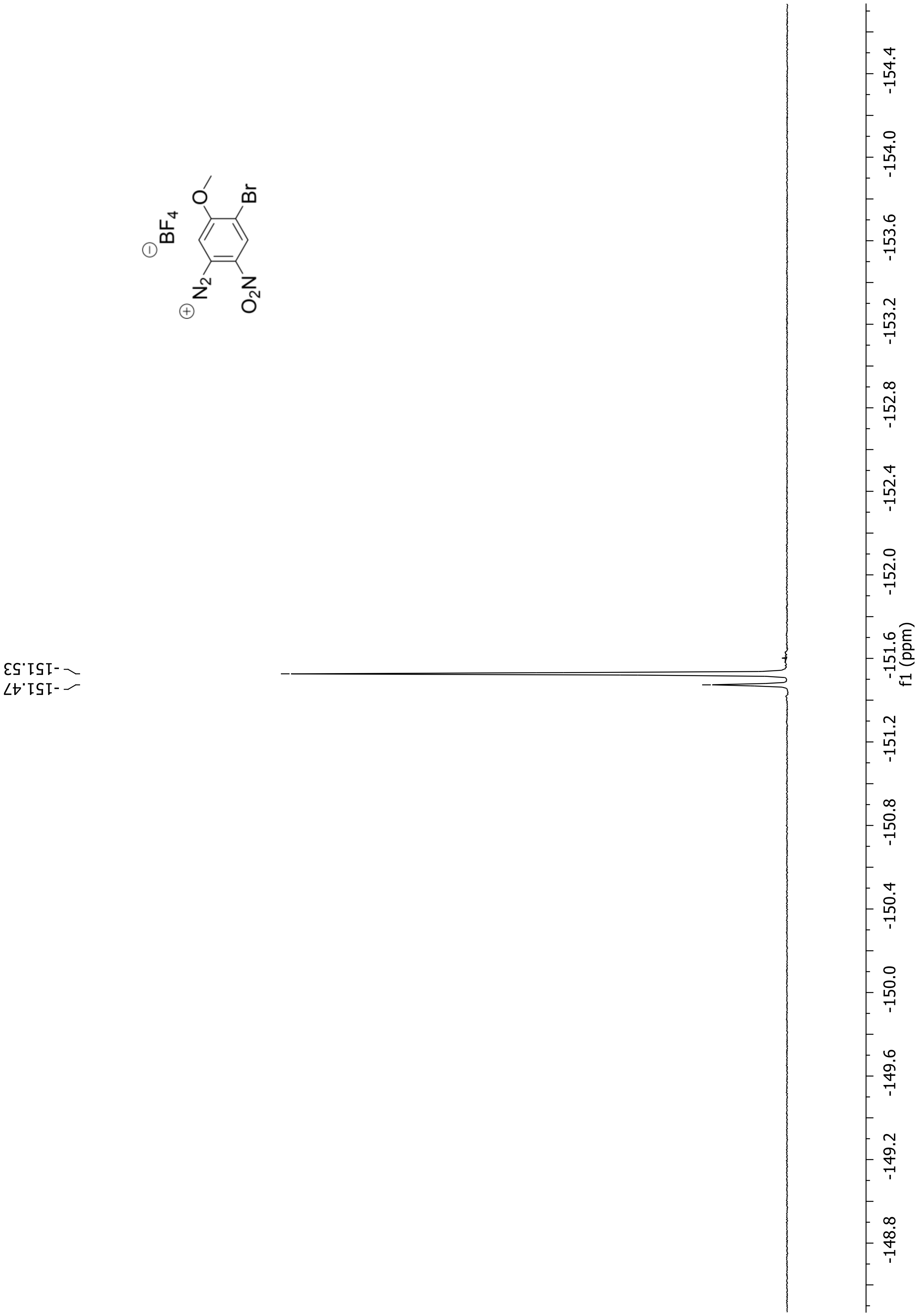

BF4
N2
O2N
O
Br
-151.53
-151.47
-154.4
-154.0
-153.6
-153.2
-152.8
-152.4
-152.0
-151.6
-151.2
-150.8
-150.4
-150.0
-149.6
-149.2
-148.8
f1 (ppm)

^{11}B NMR of **8**

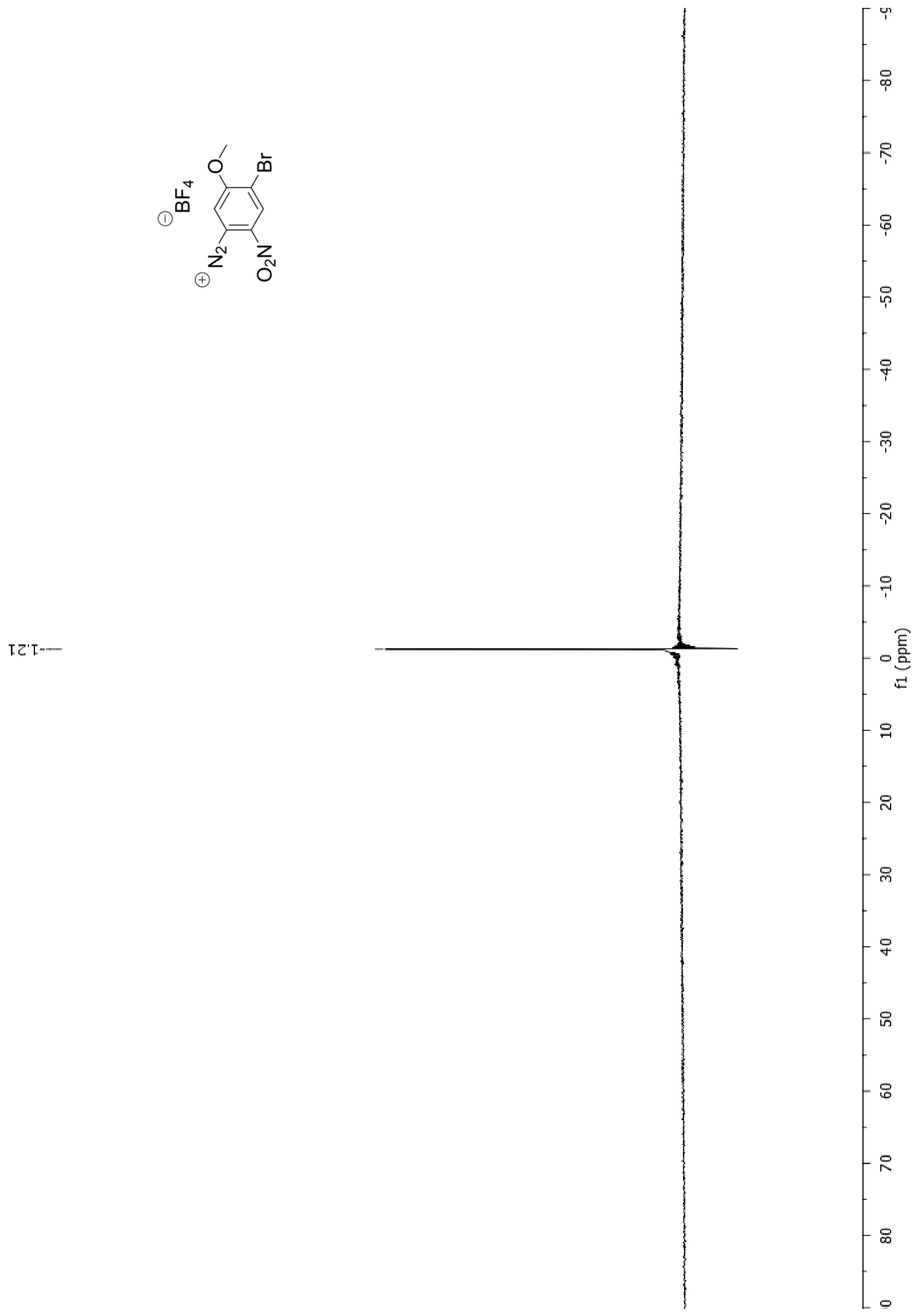

^{1}H NMR of **9**

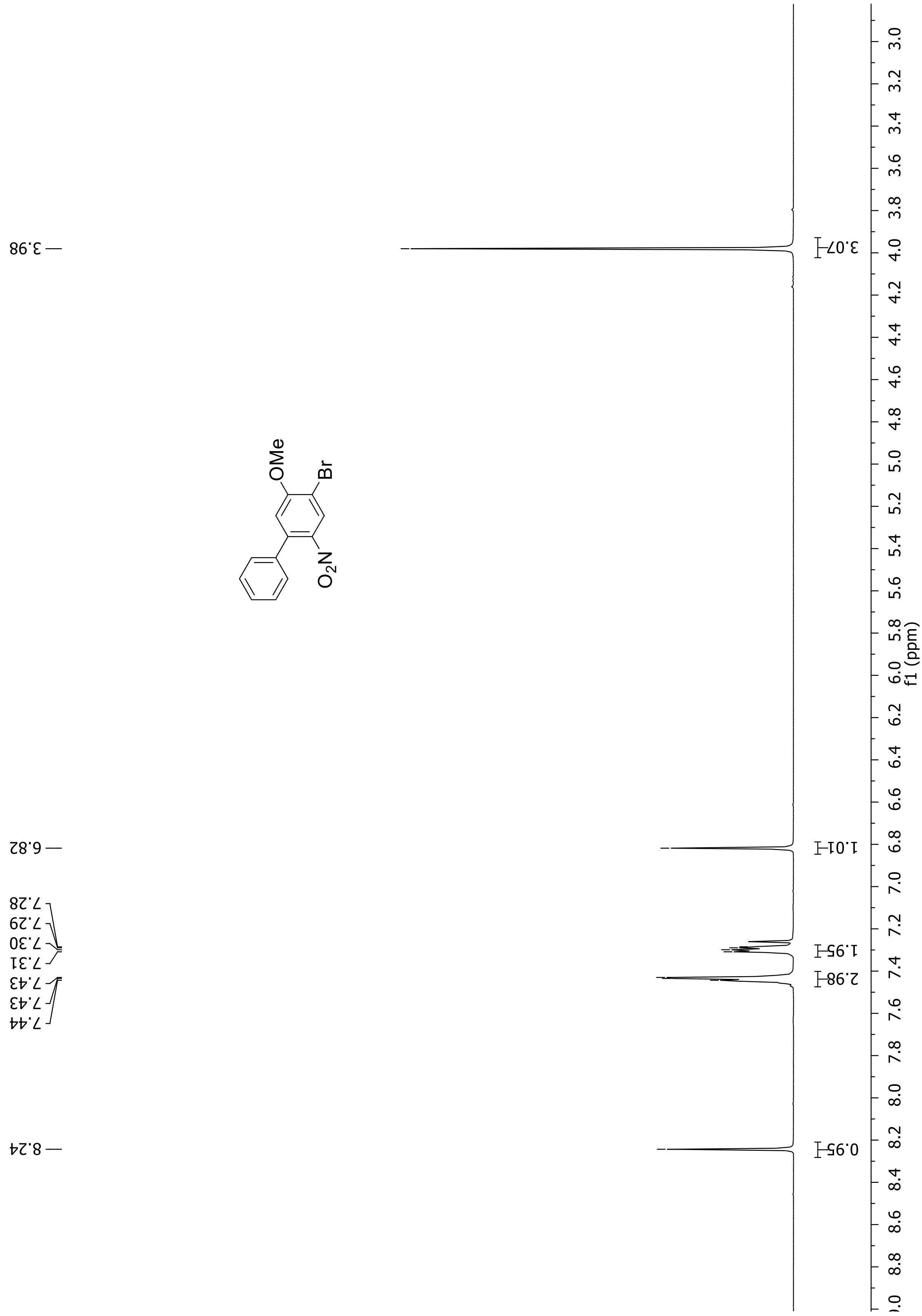
3.98
6.82
7.28
7.29
7.30
7.31
7.43
7.43
7.44
8.24
3.07
1.01
1.95
2.98
0.95
OMe
Br
O2N
f1 (ppm)

^{13}C NMR of **9**

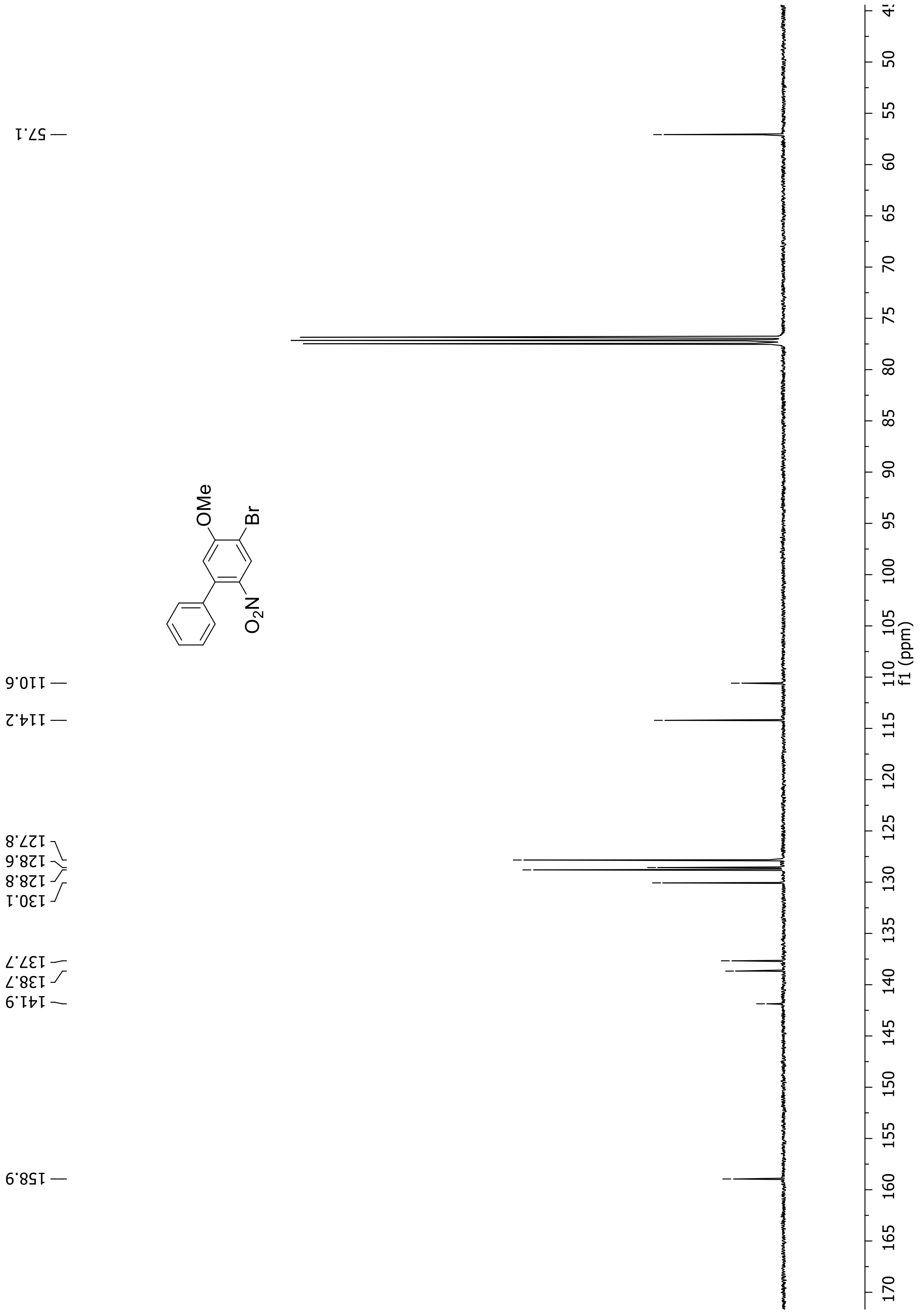
57.1
110.6
114.2
127.8
128.6
128.8
130.1
137.7
138.7
141.9
158.9
OMe
Br
O_2N
f1 (ppm)

^{1}H NMR of **10**

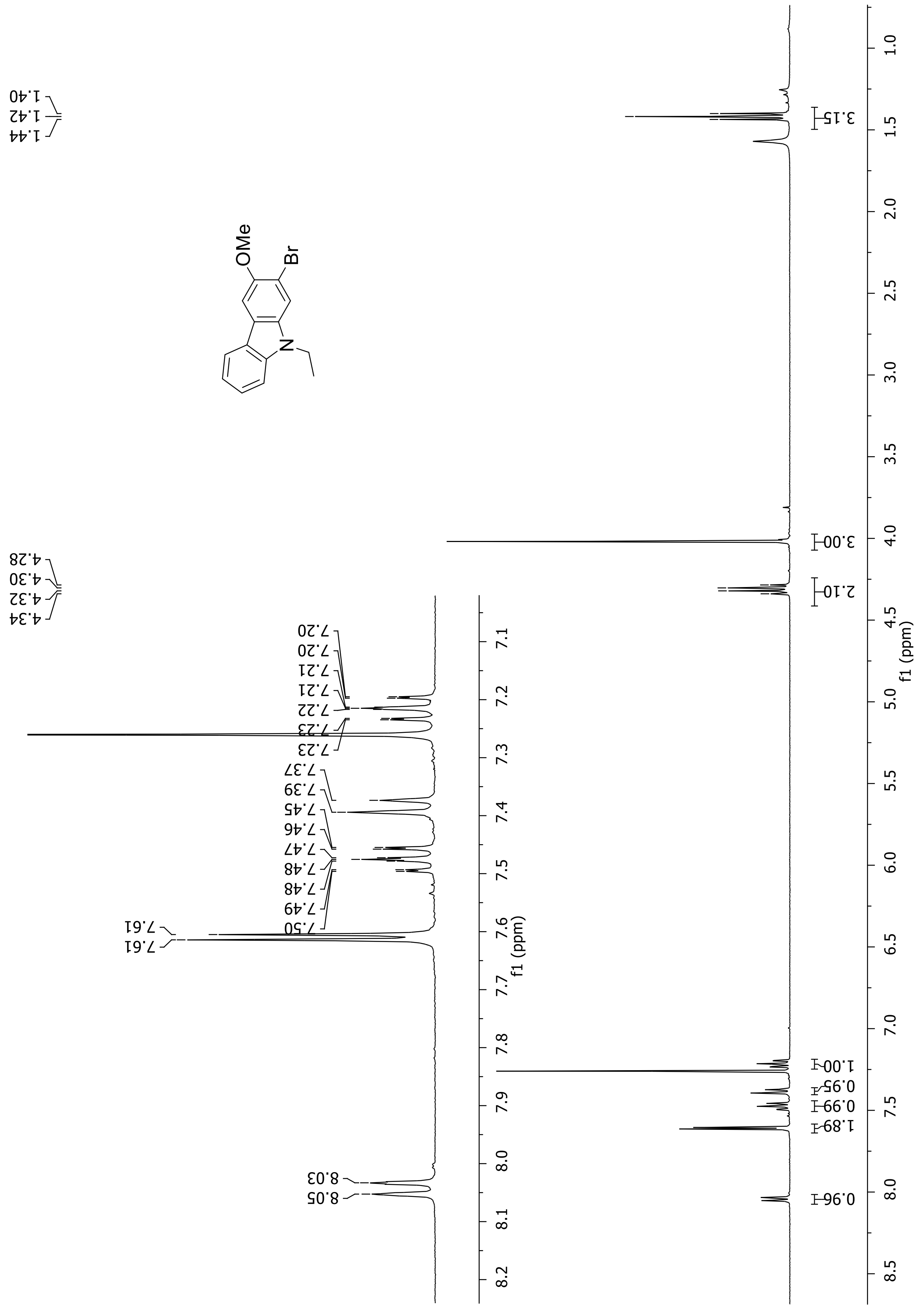

^{13}C NMR of **10**

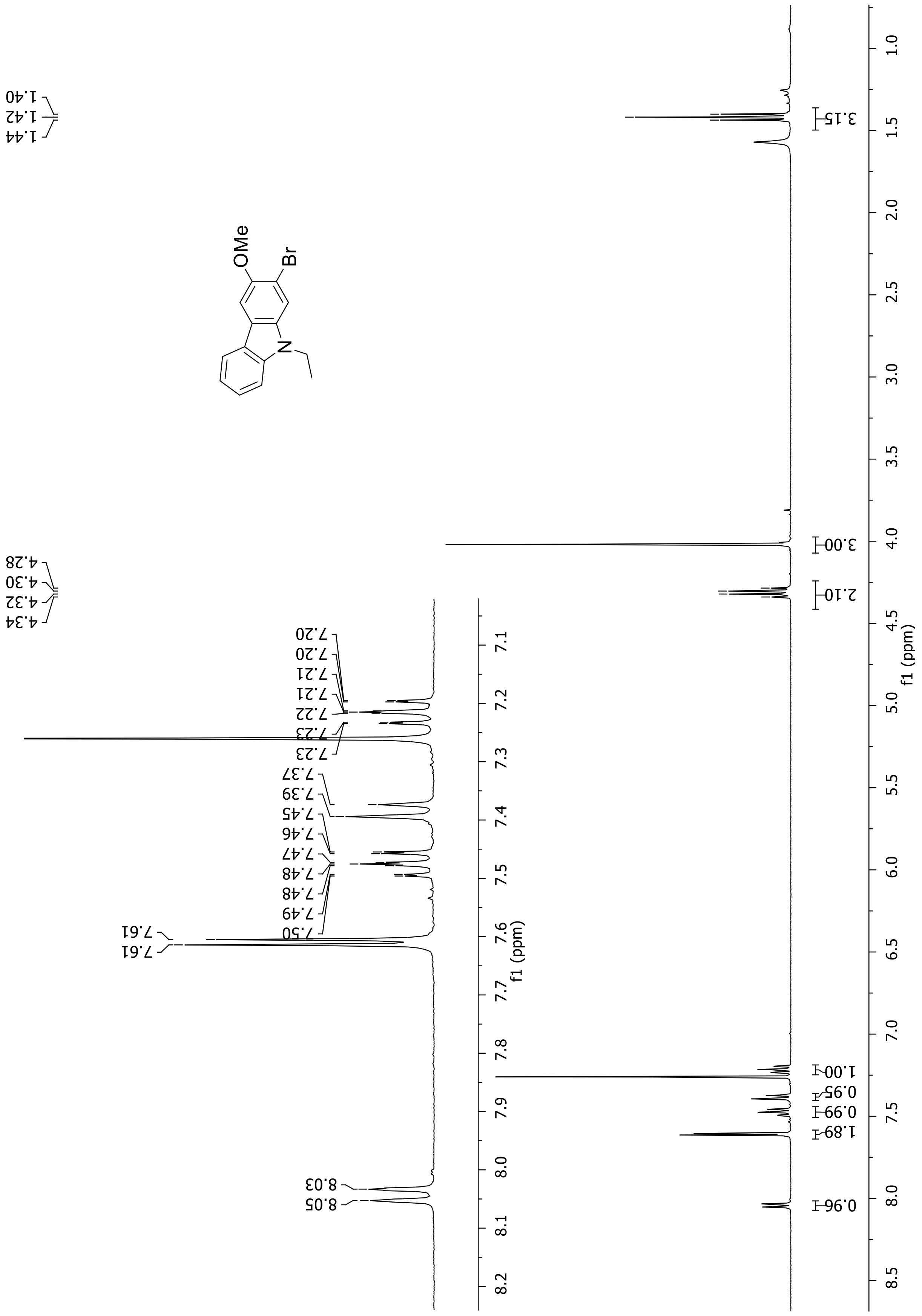

^{1}H NMR of **22Cz**

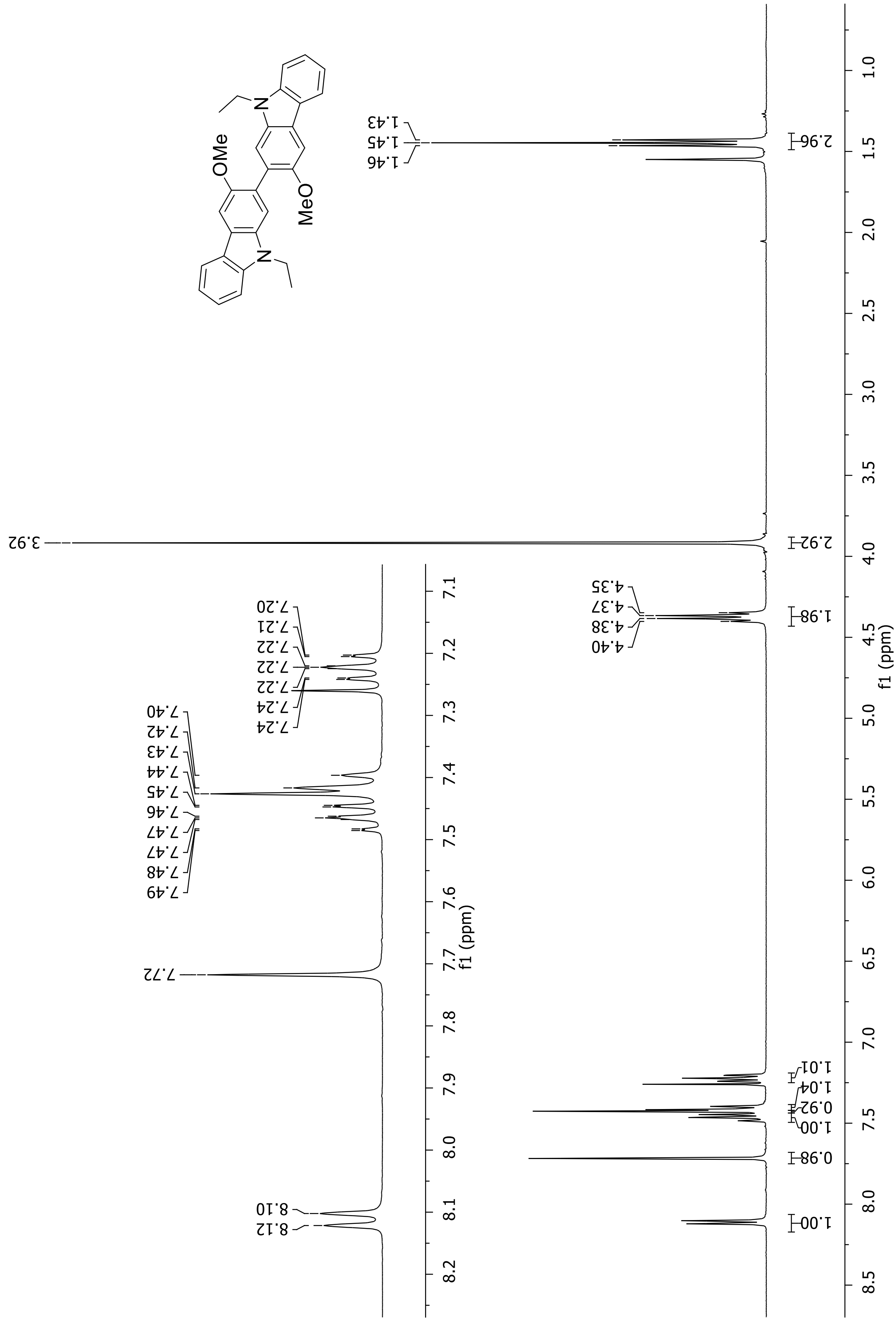

^{13}C NMR of **22Cz**

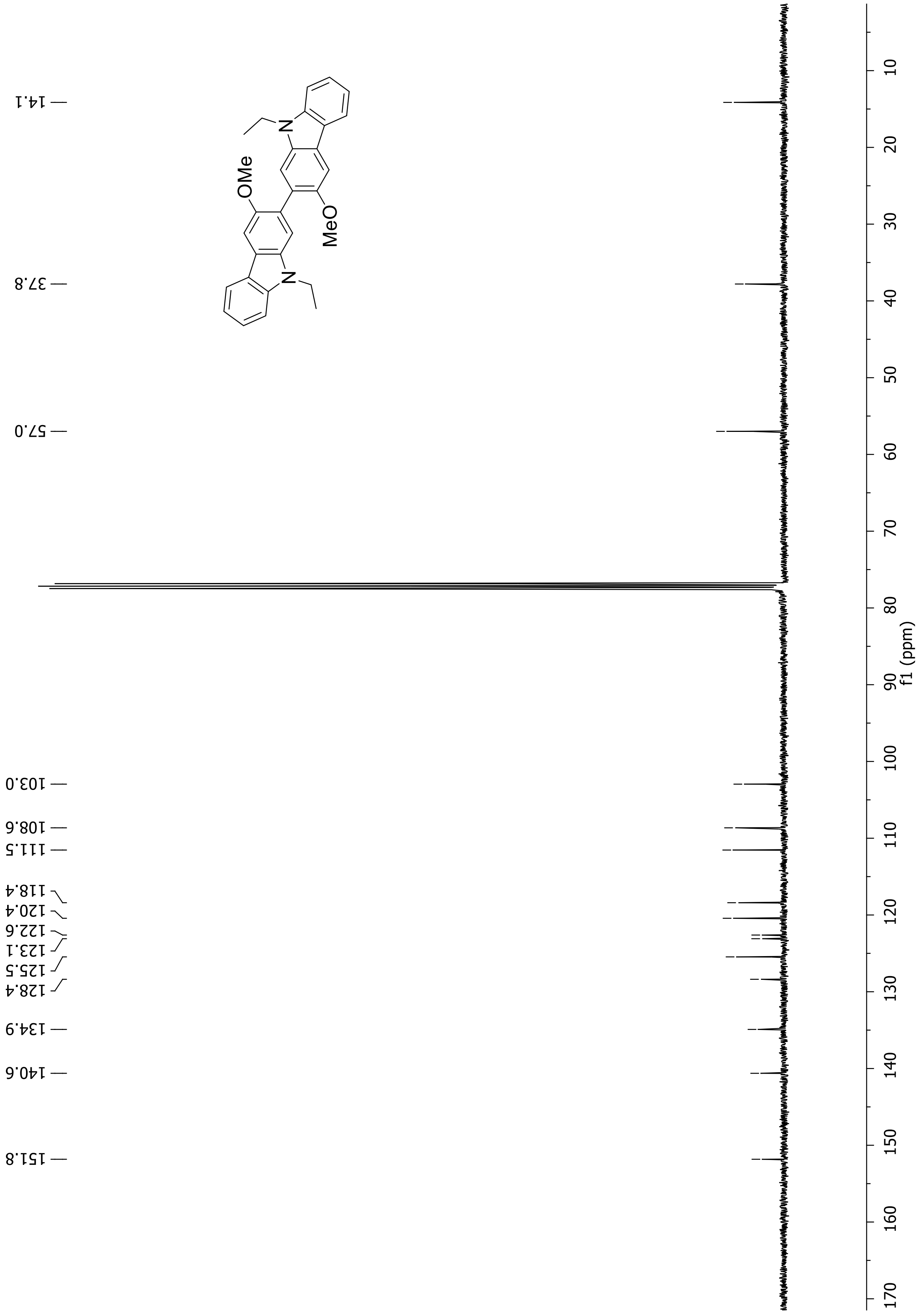

^{1}H NMR of **7**

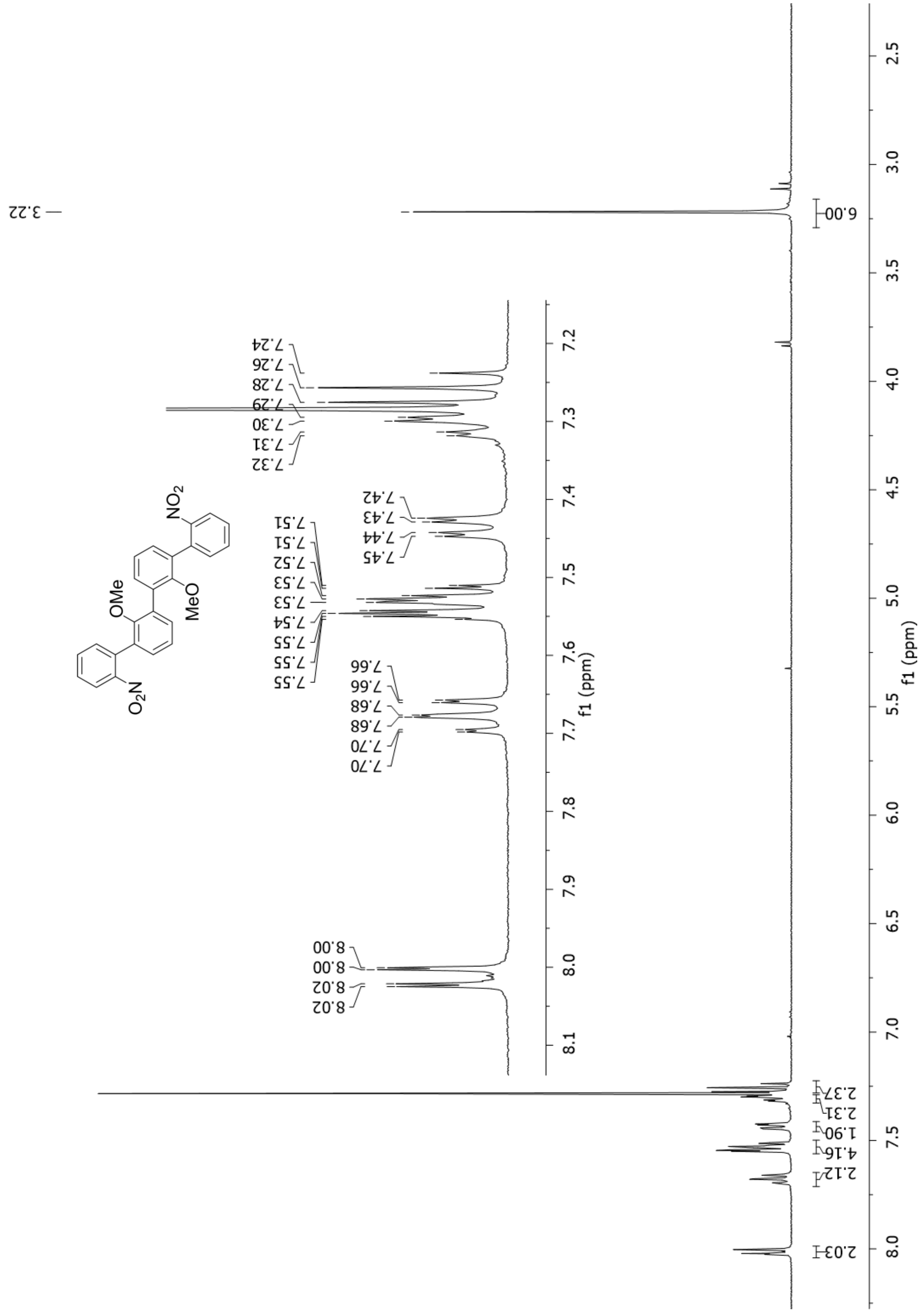

^{13}C NMR of **7**

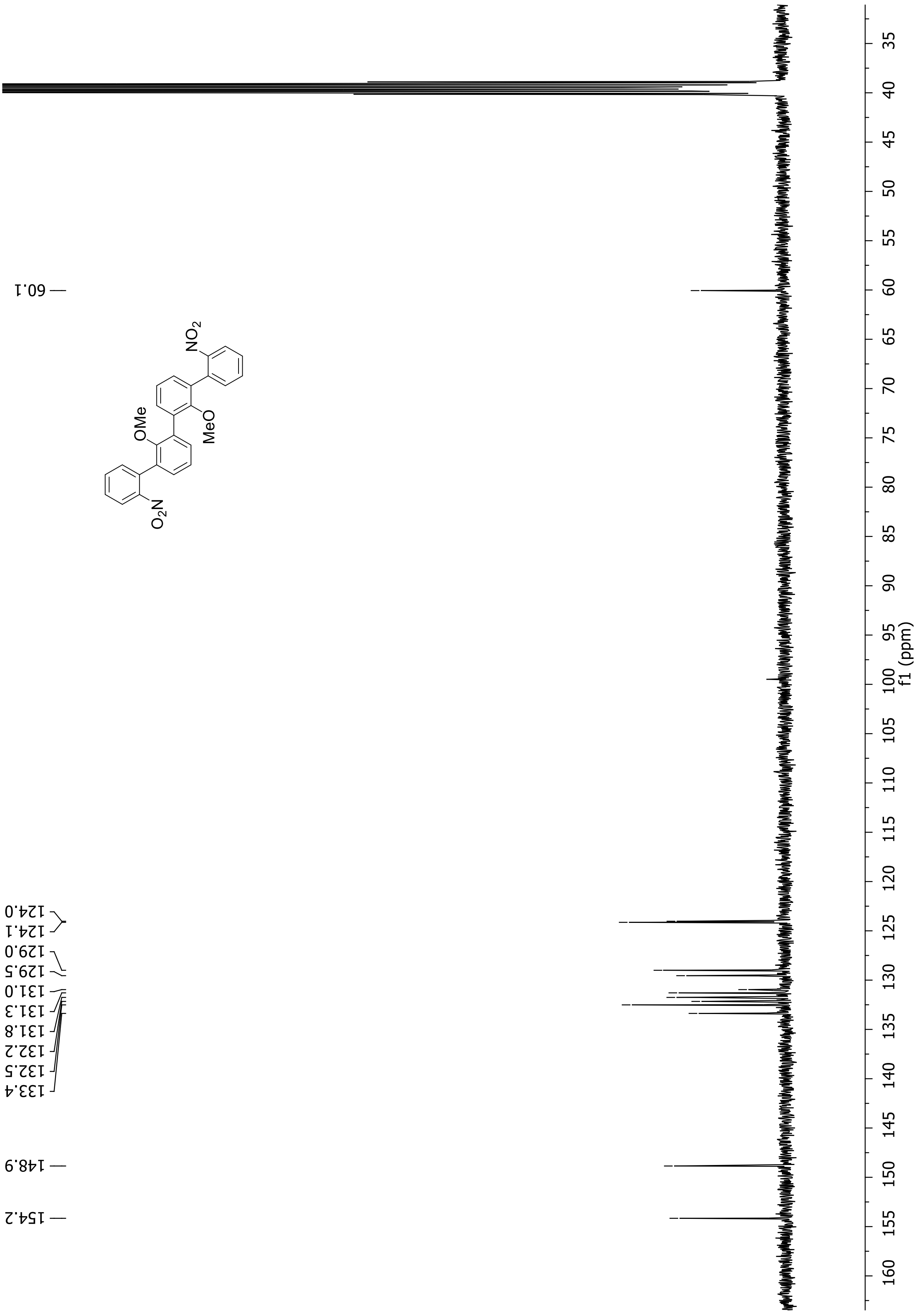
60.1
124.0
124.1
129.0
129.5
131.0
131.3
131.8
132.2
132.5
133.4
148.9
154.2
NO_2
OMe
MeO
O_2N
f1 (ppm)

^{1}H NMR of **33Cz**

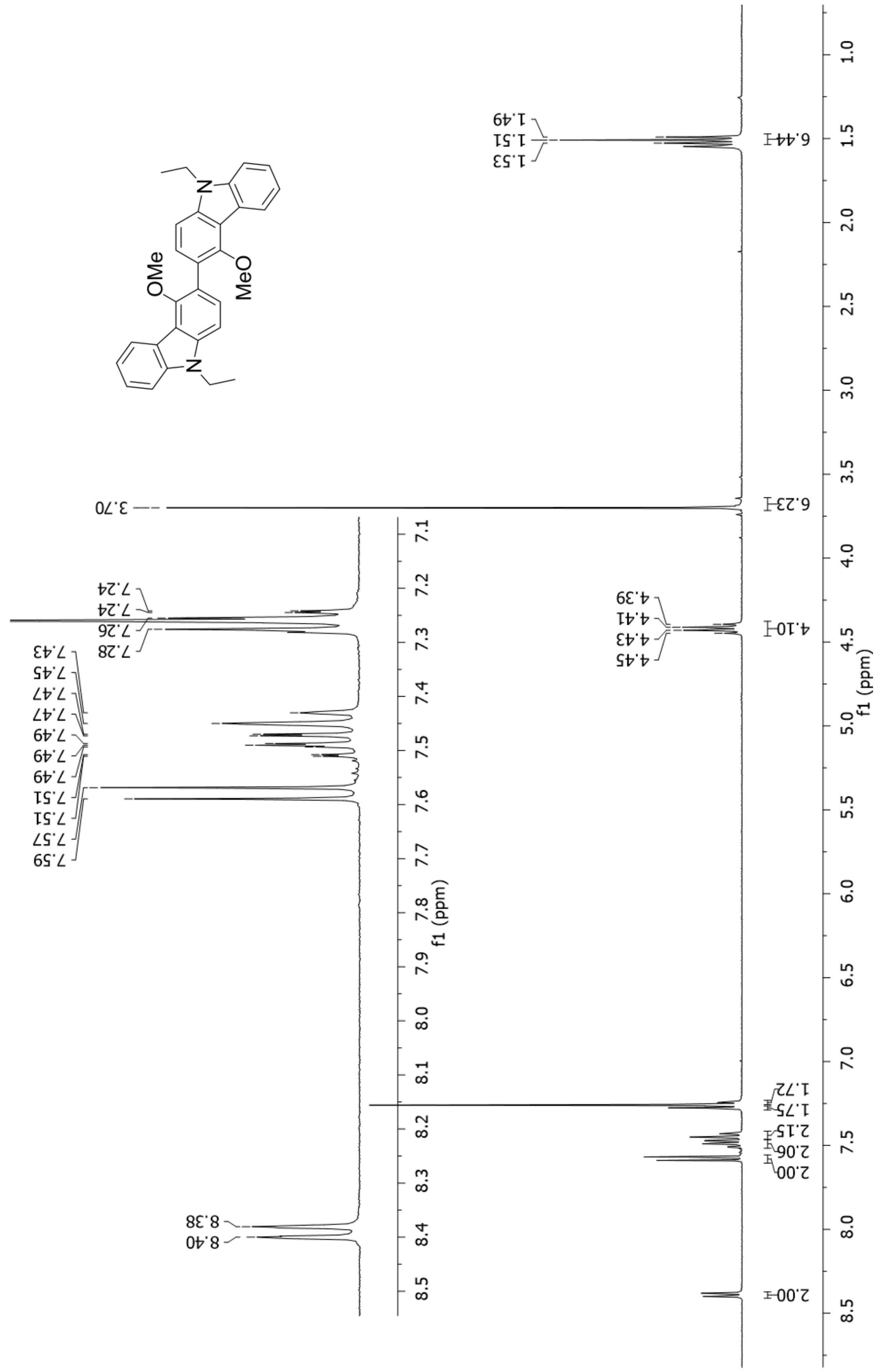

^{13}C NMR of **33Cz**

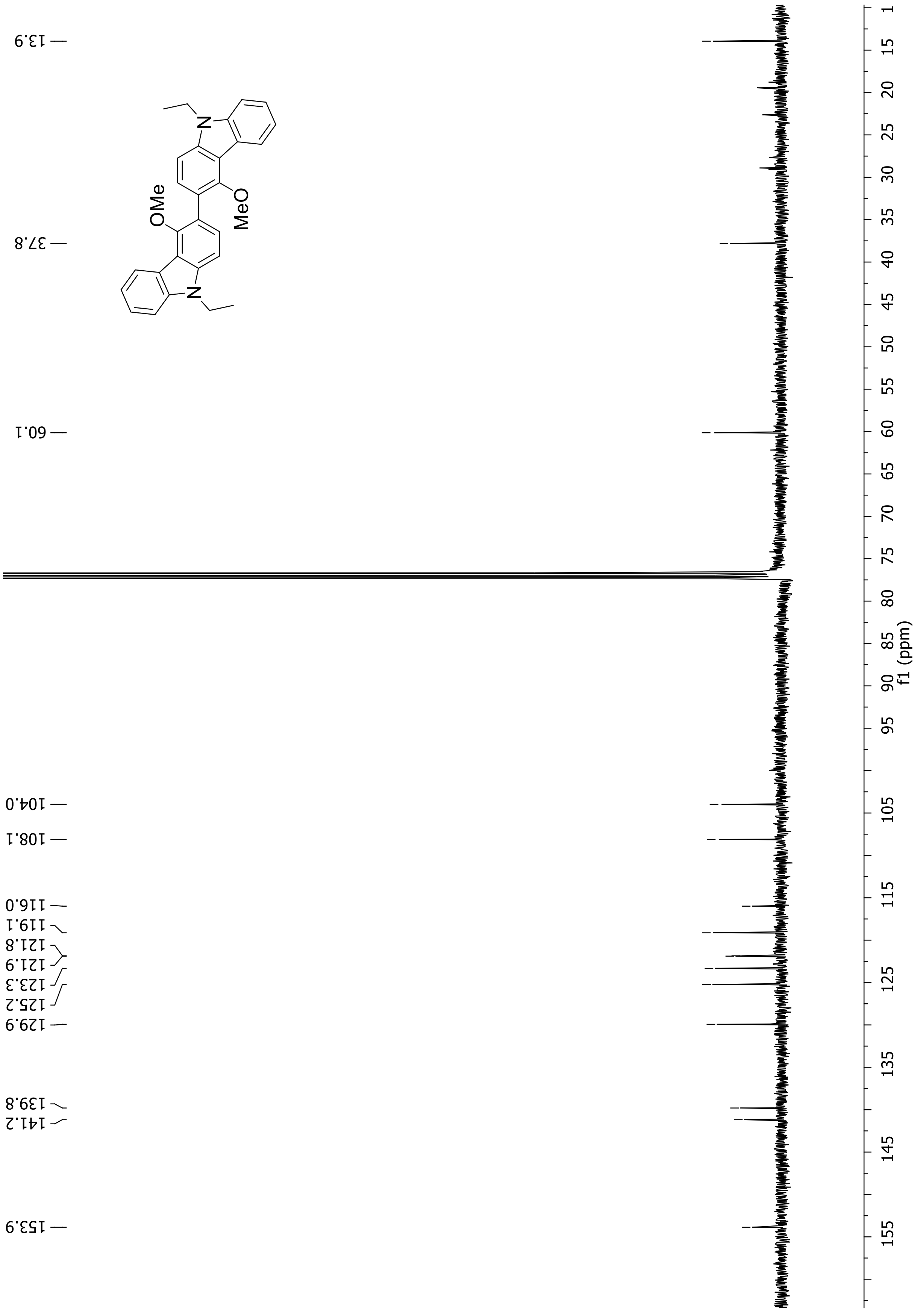

^{1}H NMR of **12**

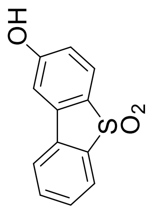
OH
S
O2

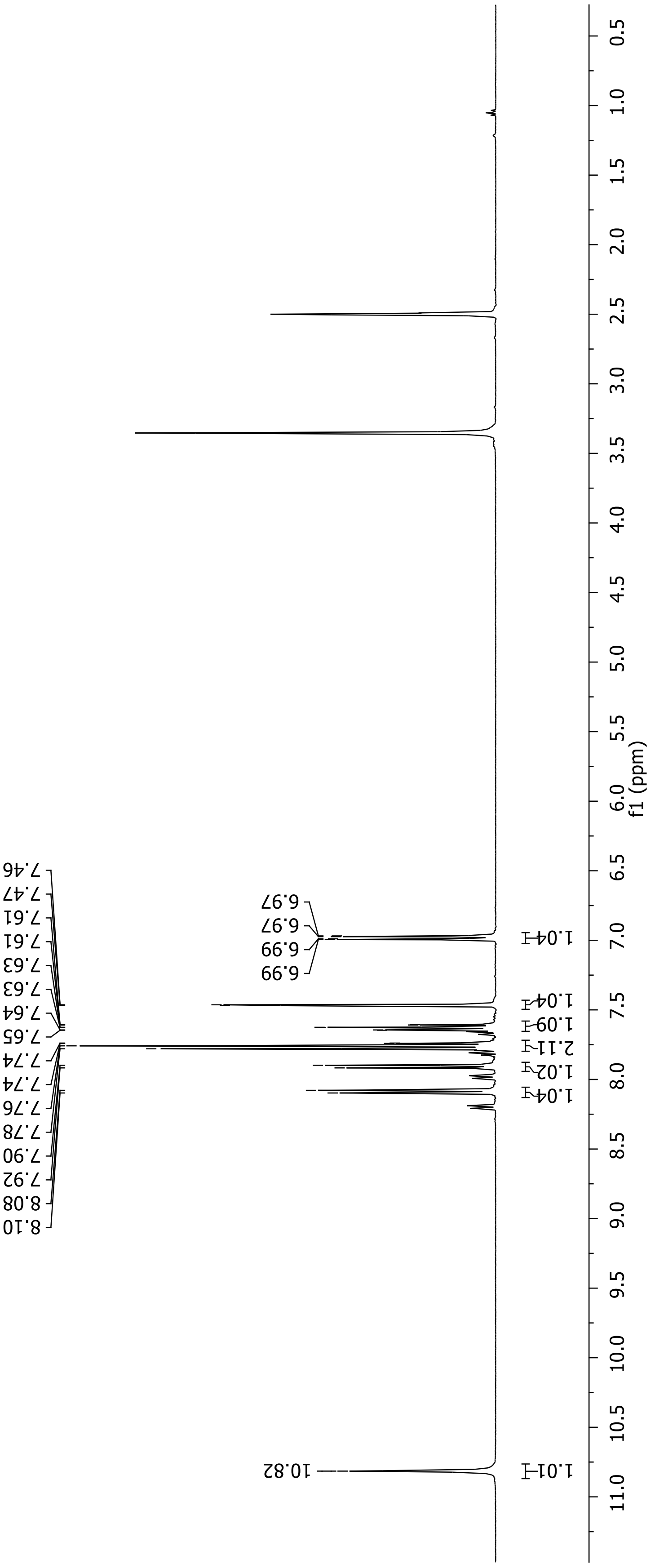
f1 (ppm)
10.82
6.97
6.97
6.99
6.99
7.46
7.47
7.61
7.61
7.63
7.63
7.64
7.65
7.74
7.74
7.76
7.78
7.90
7.92
8.08
8.10
1.04
1.04
1.09
2.11
1.02
1.04
1.01

^{13}C NMR of **12**

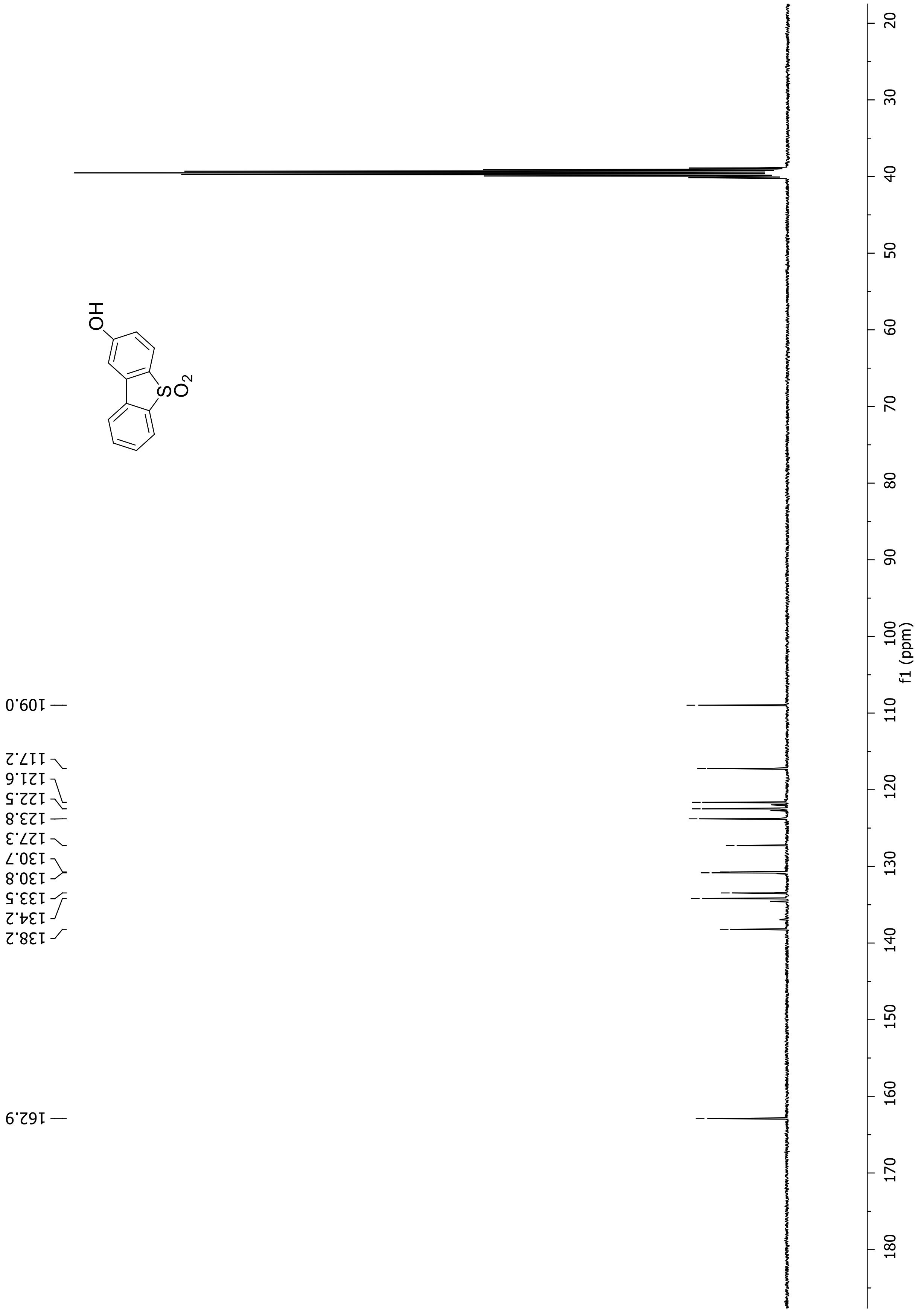

OH
SO2
109.0
117.2
121.6
122.5
123.8
127.3
130.7
130.8
133.5
134.2
138.2
162.9
f1 (ppm)
20
30
40
50
60
70
80
90
100
110
120
130
140
150
160
170
180

^{1}H NMR of **13**

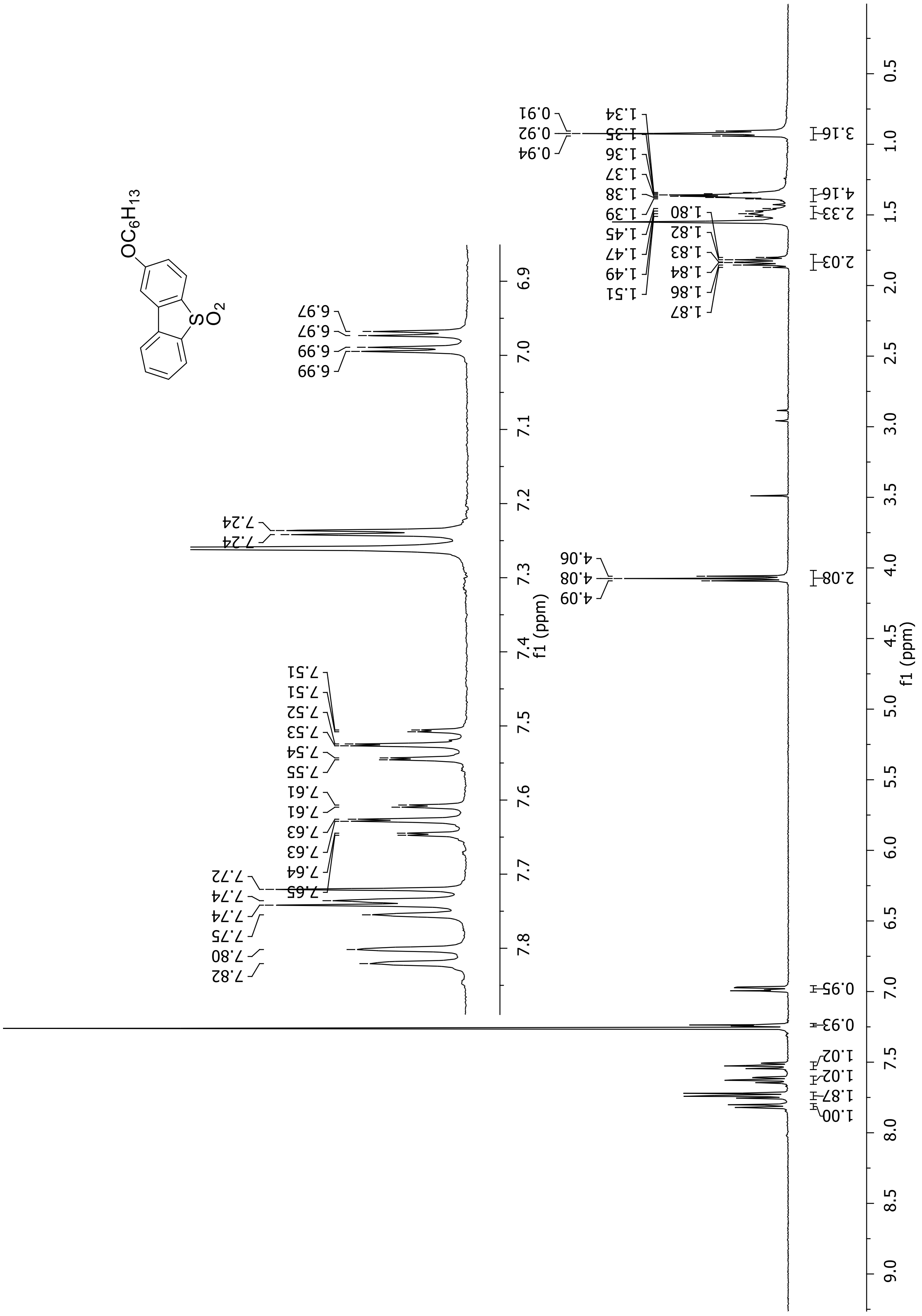

^{13}C NMR of **13**

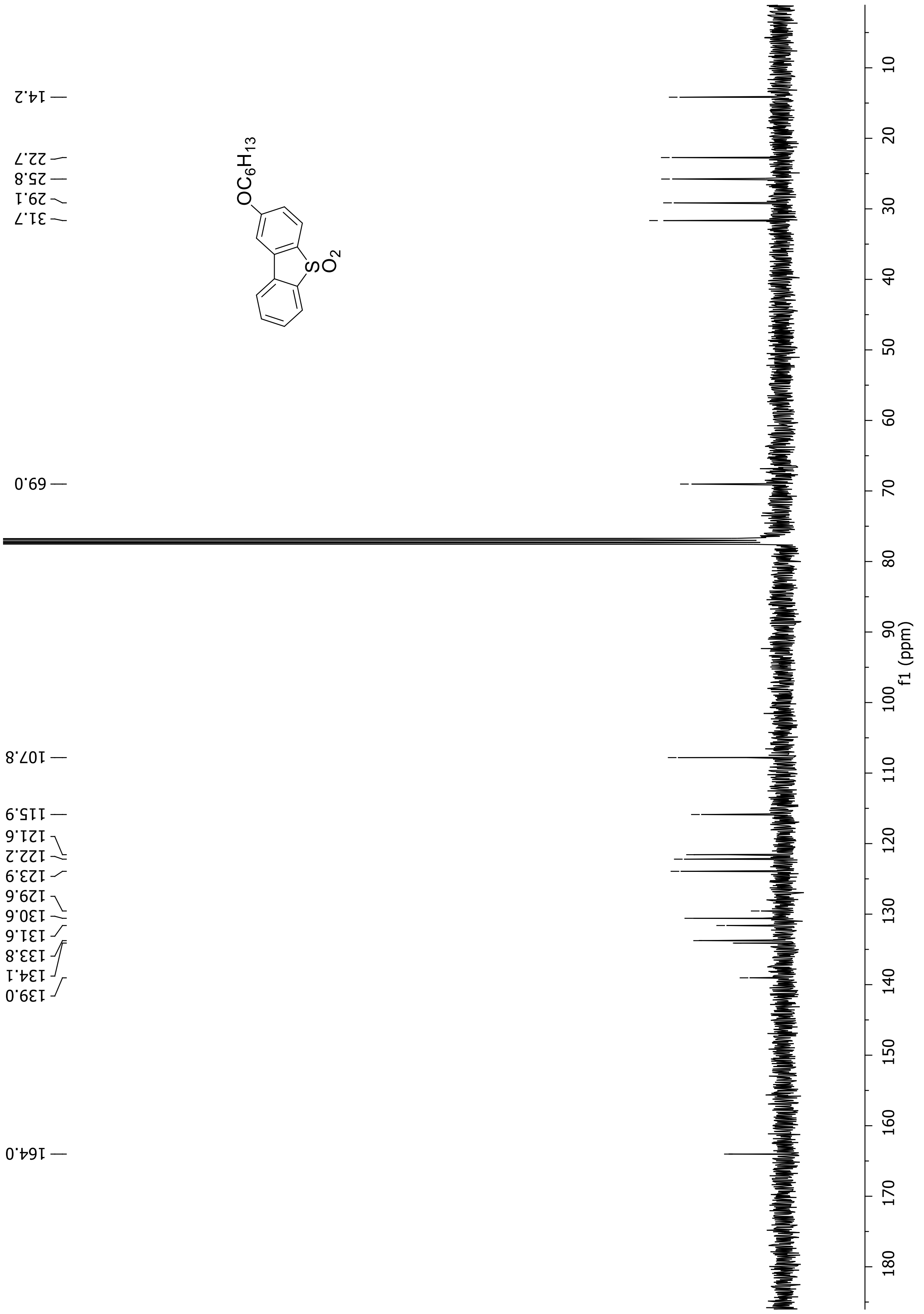
OC6H13
SO2
14.2
22.7
25.8
29.1
31.7
69.0
107.8
115.9
121.6
122.2
123.9
129.6
130.6
131.6
133.8
134.1
139.0
164.0
f1 (ppm)

^{1}H NMR of **14**

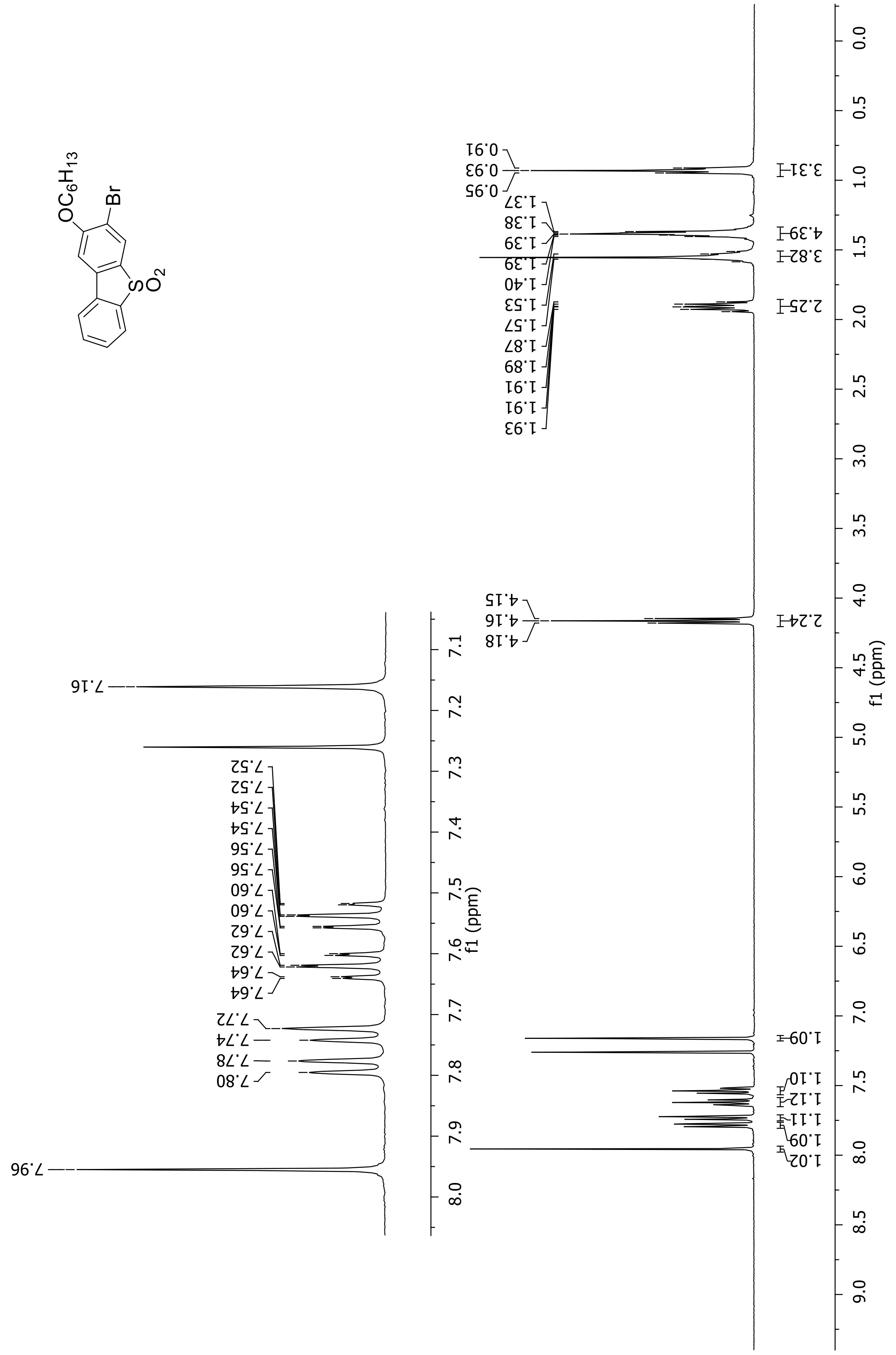

^{13}C NMR of **14**

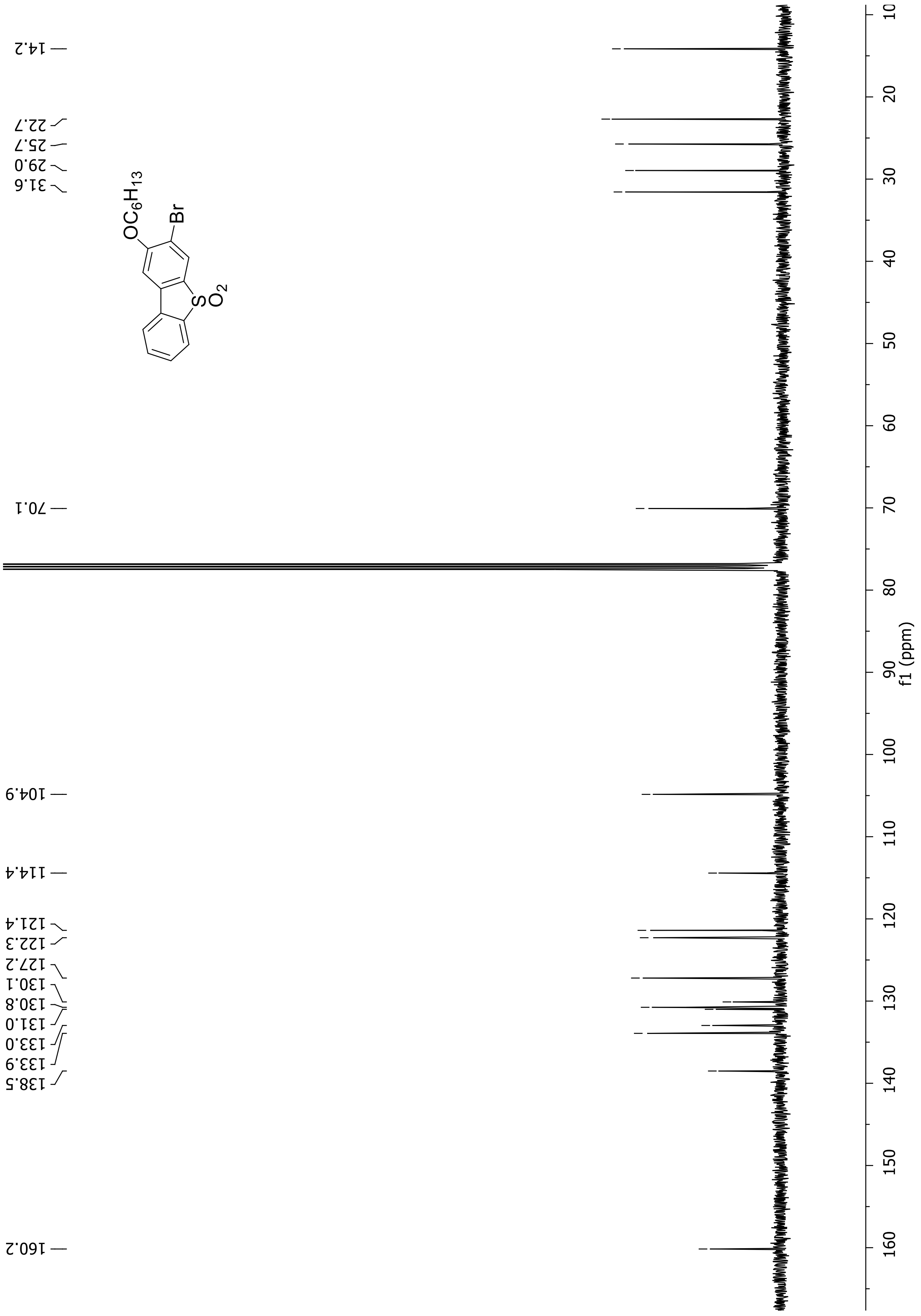
14.2
22.7
25.7
29.0
31.6
70.1
104.9
114.4
121.4
122.3
127.2
130.1
130.8
131.0
133.0
133.9
138.5
160.2
OC6H13
Br
S
O2
f1 (ppm)

^{1}H NMR of **15**

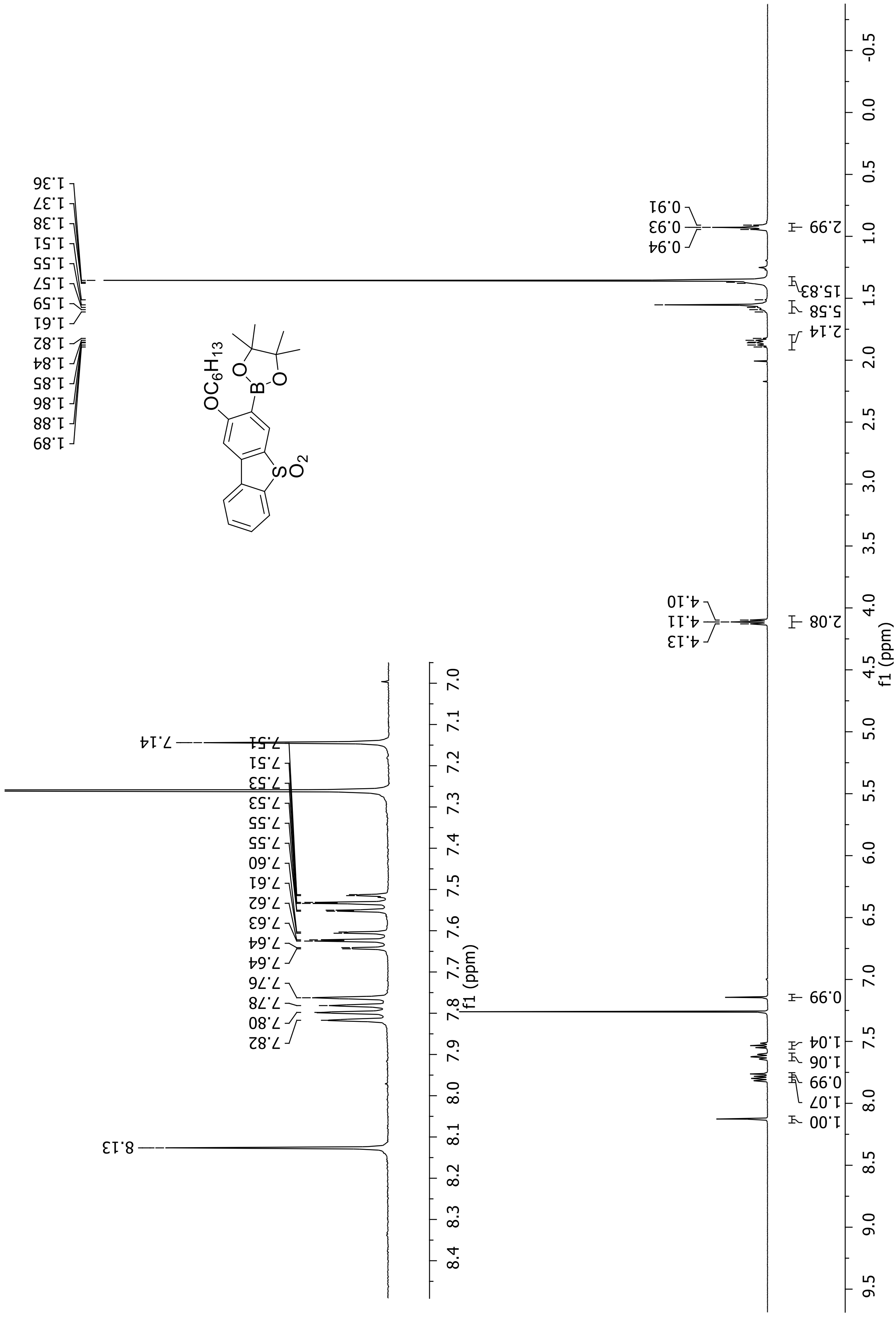
1.36
1.37
1.38
1.51
1.55
1.57
1.59
1.61
1.82
1.84
1.85
1.86
1.88
1.89
OC6H13
O
B
O
S
O2
0.91
0.93
0.94
4.10
4.11
4.13
7.14
7.51
7.51
7.53
7.53
7.55
7.55
7.60
7.61
7.62
7.63
7.64
7.64
7.76
7.78
7.80
7.82
8.13
2.99
15.83
5.58
2.14
2.08
0.99
1.04
1.06
0.99
1.07
1.00
f1 (ppm)

^{13}C NMR of **15**

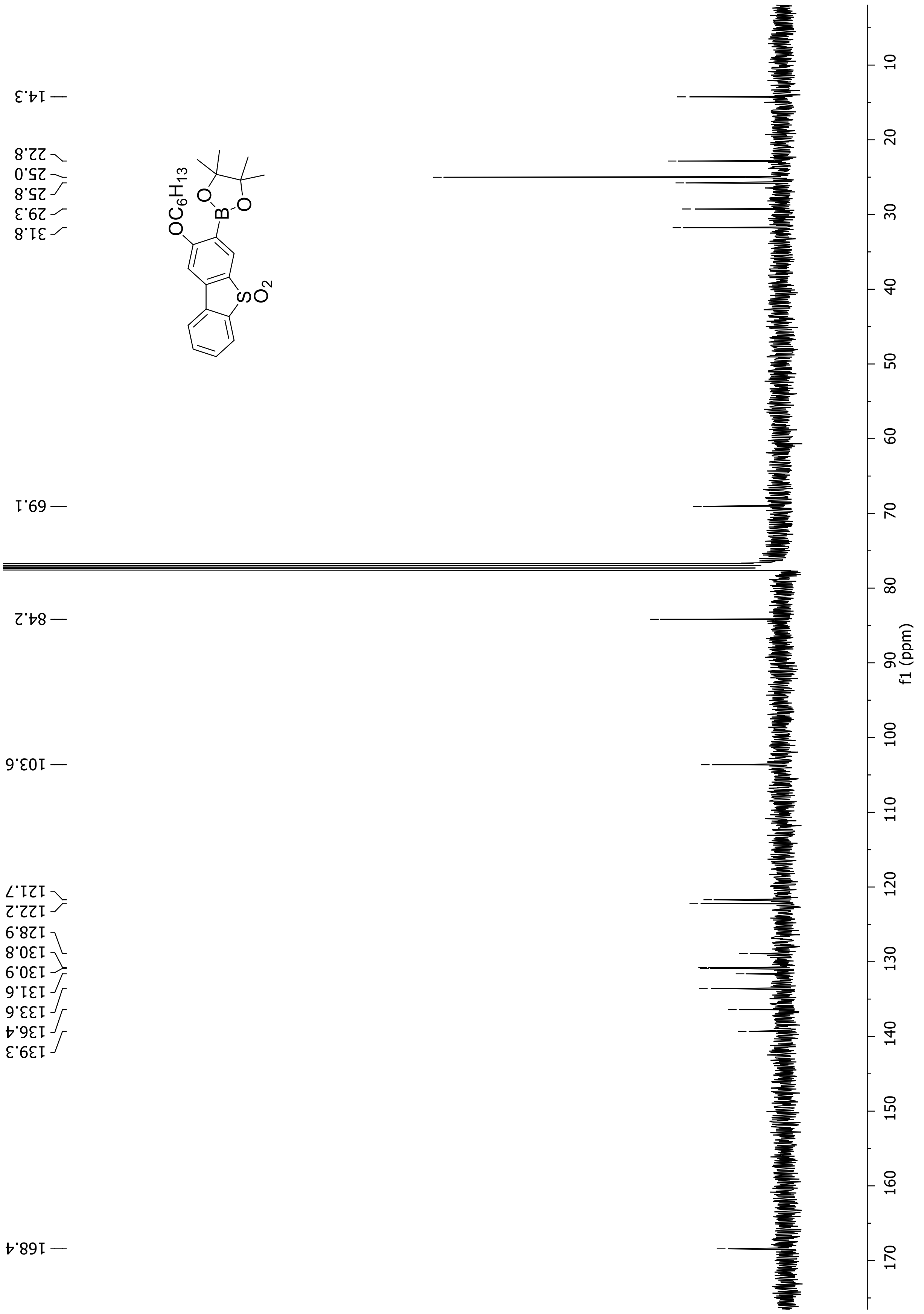
14.3
22.8
25.0
25.8
29.3
31.8
69.1
84.2
103.6
121.7
122.2
128.9
130.8
130.9
131.6
133.6
136.4
139.3
168.4
OC6H13
O
B
O
S
O2
f1 (ppm)

^{11}B NMR of **15**

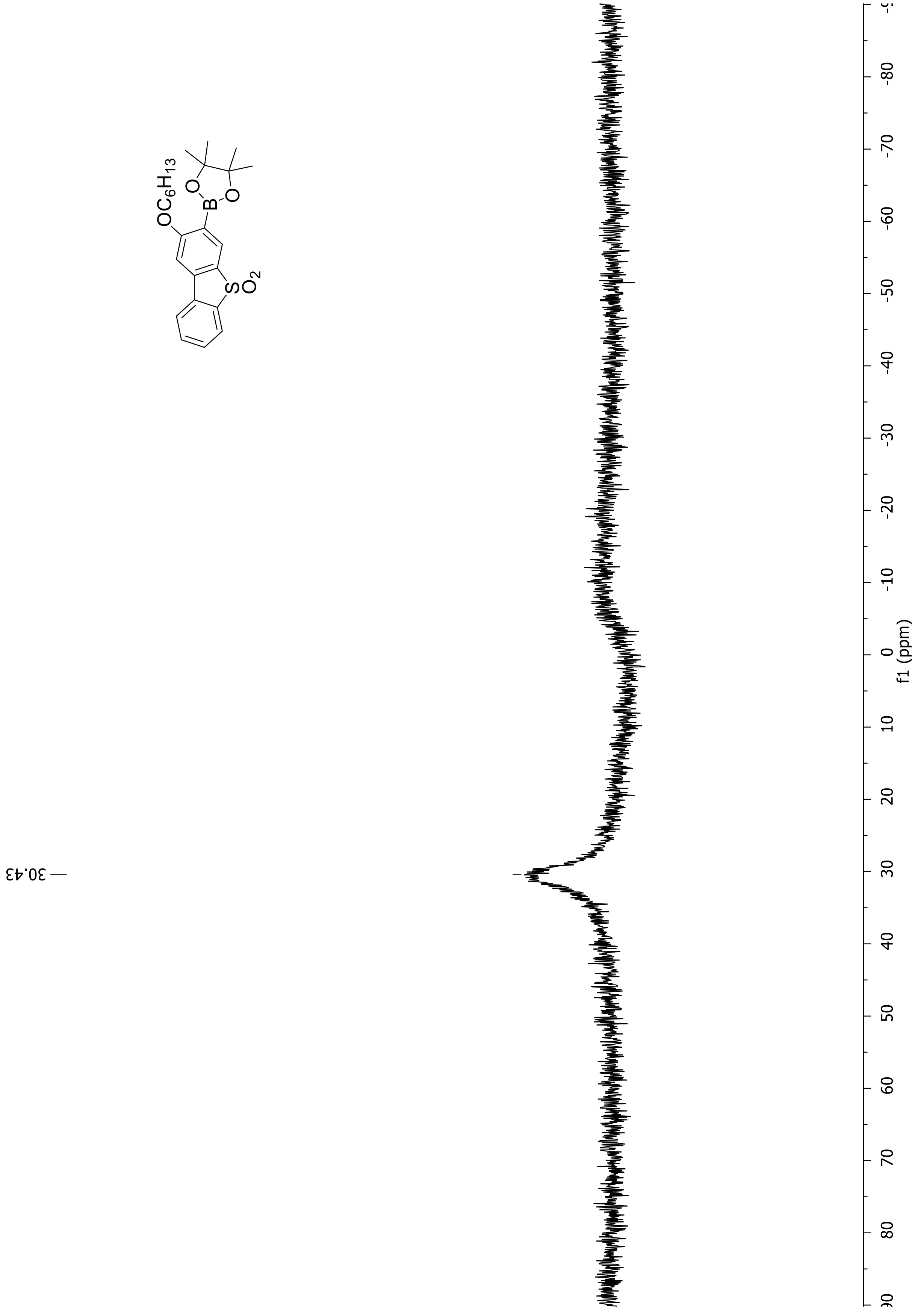

^{1}H NMR of **33DBS**

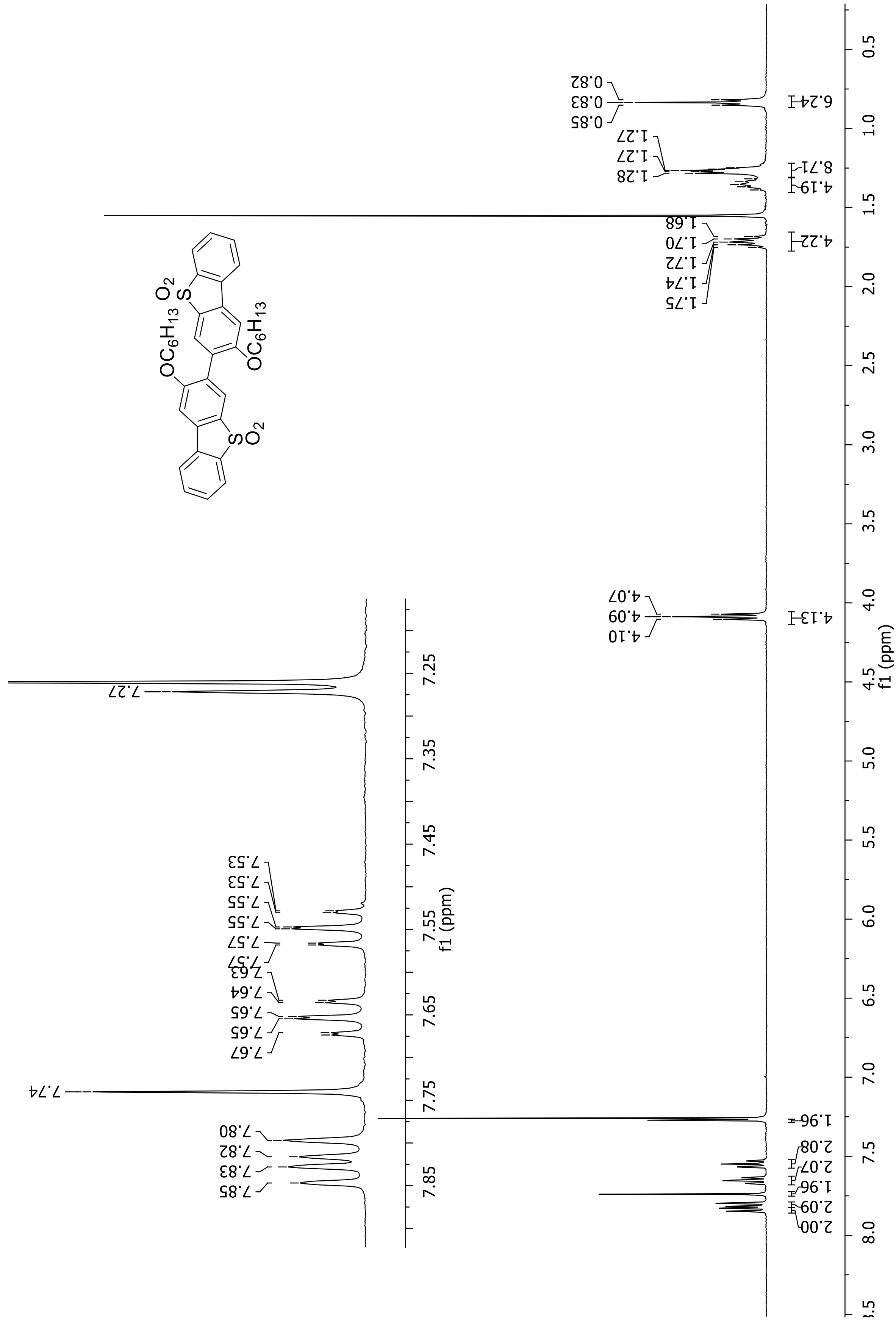

^{13}C NMR of **33DBS**

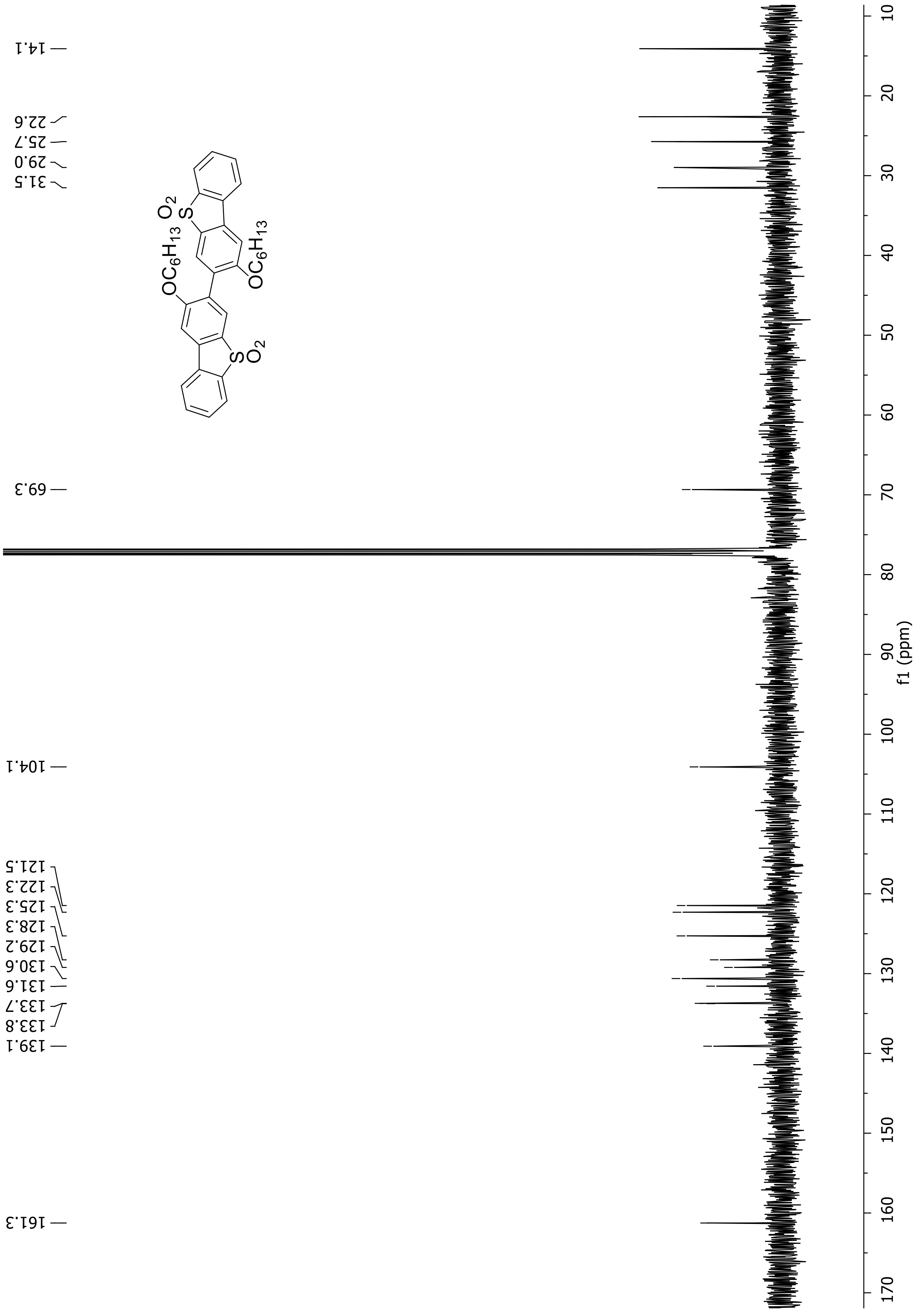

^{1}H NMR of **17**

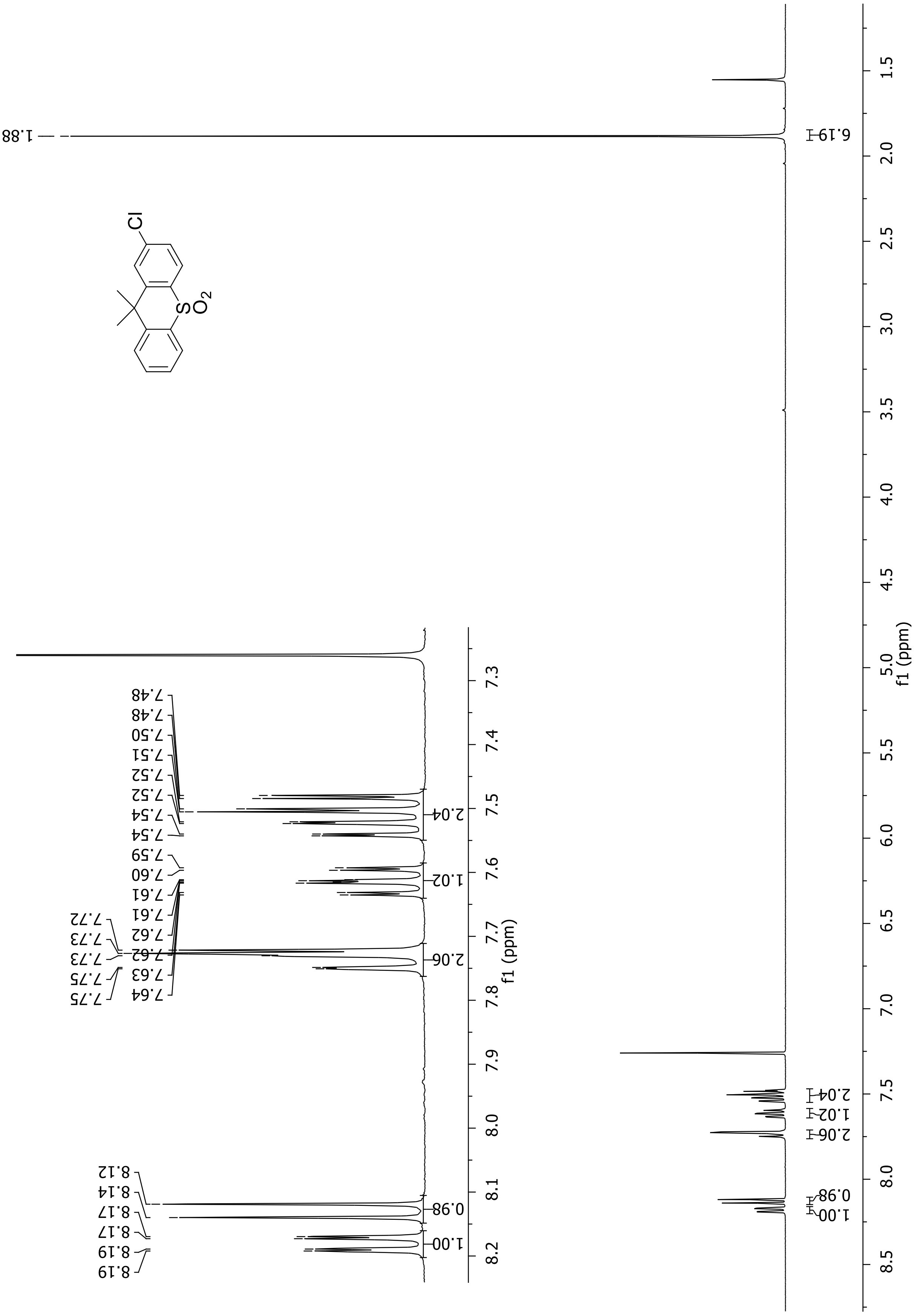
Cl
S
O2
1.88
7.48
7.48
7.50
7.51
7.52
7.52
7.54
7.54
7.59
7.60
7.61
7.61
7.62
7.62
7.63
7.64
7.72
7.73
7.73
7.75
7.75
8.12
8.14
8.17
8.17
8.19
8.19
2.04
1.02
2.06
0.98
1.00
6.19
f1 (ppm)

^{13}C NMR of **17**

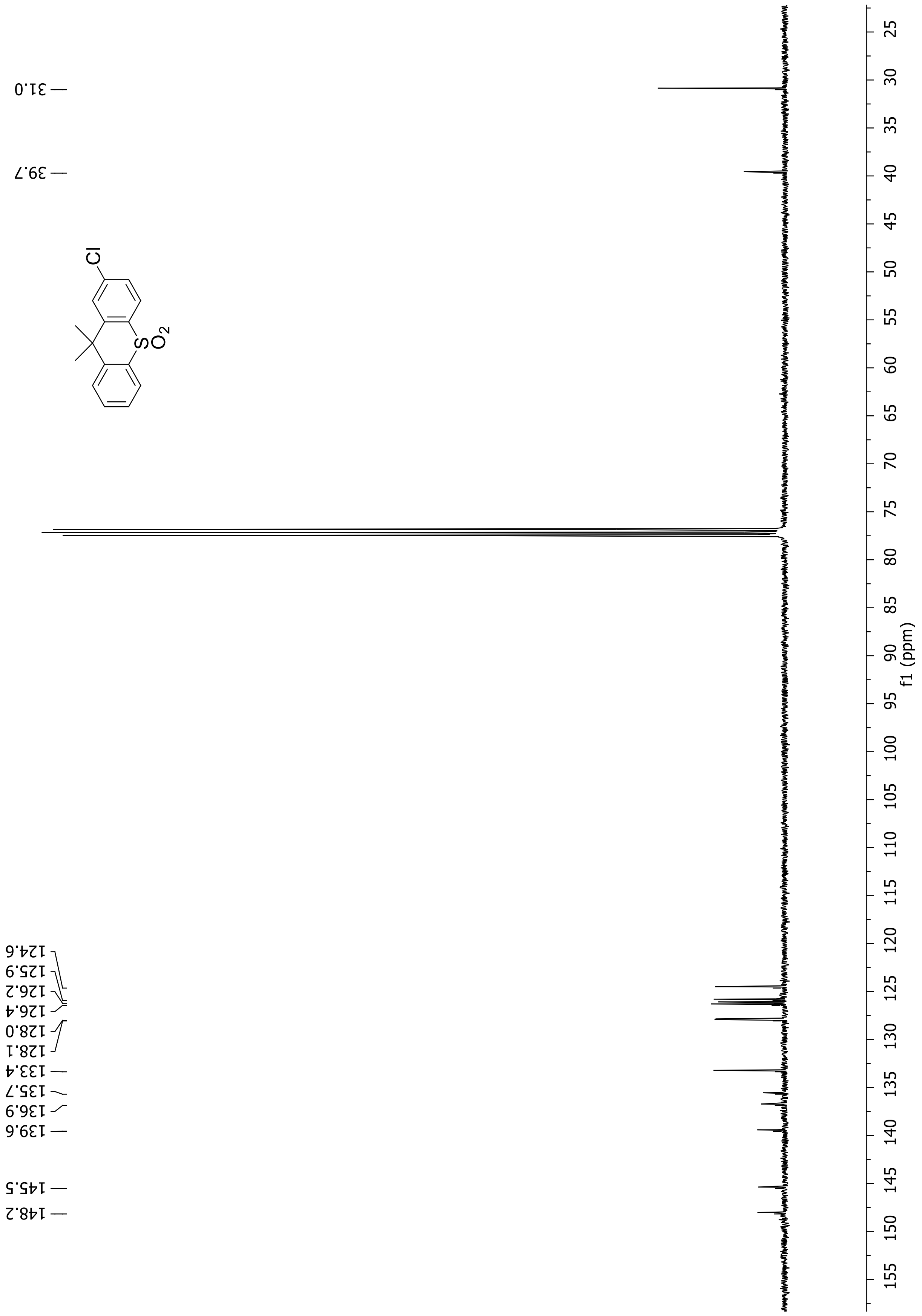
31.0
39.7
124.6
125.9
126.2
126.4
128.0
128.1
133.4
135.7
136.9
139.6
145.5
148.2
Cl
S
O2
f1 (ppm)

^{1}H NMR of **18**

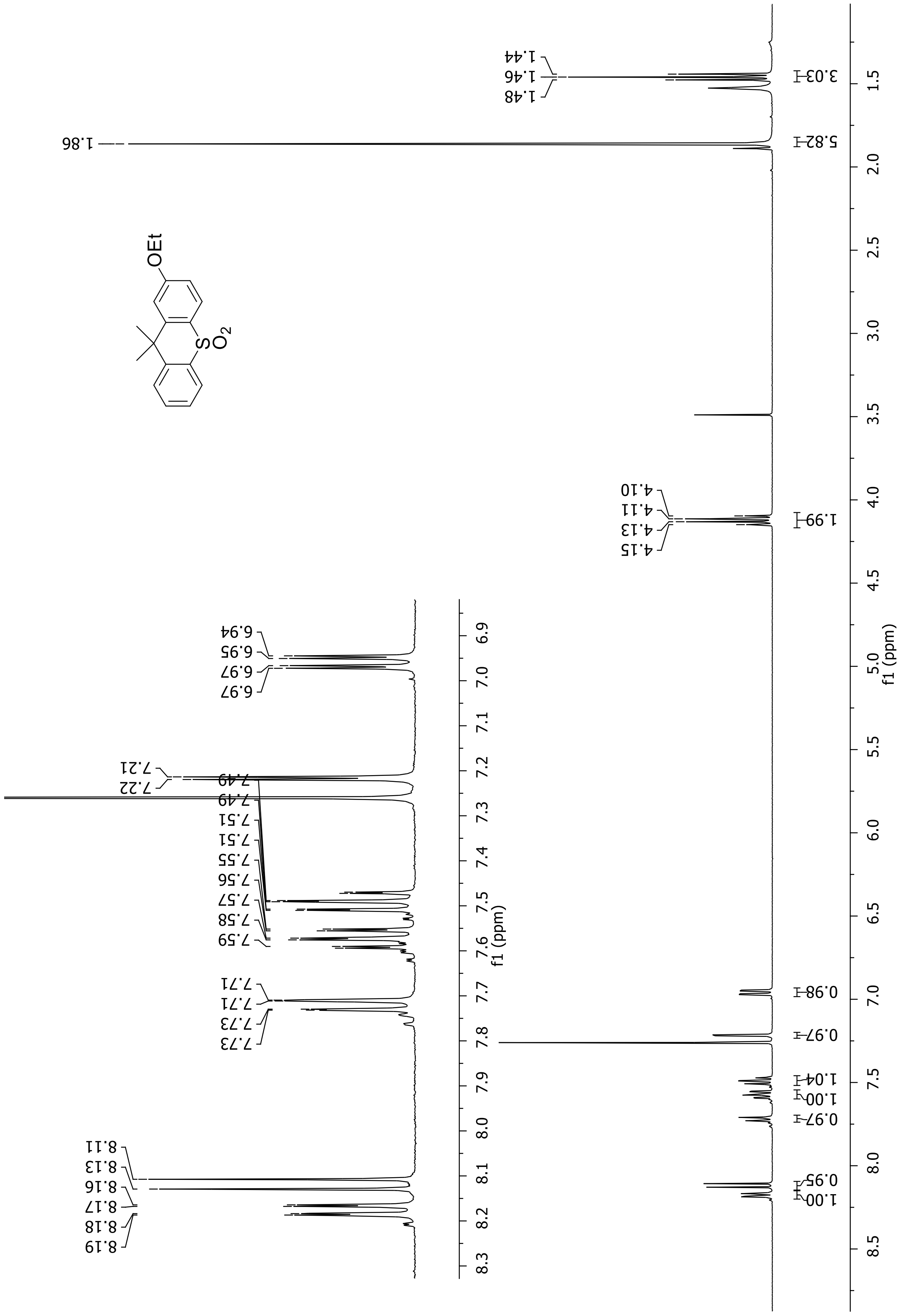

^{13}C NMR of **18**

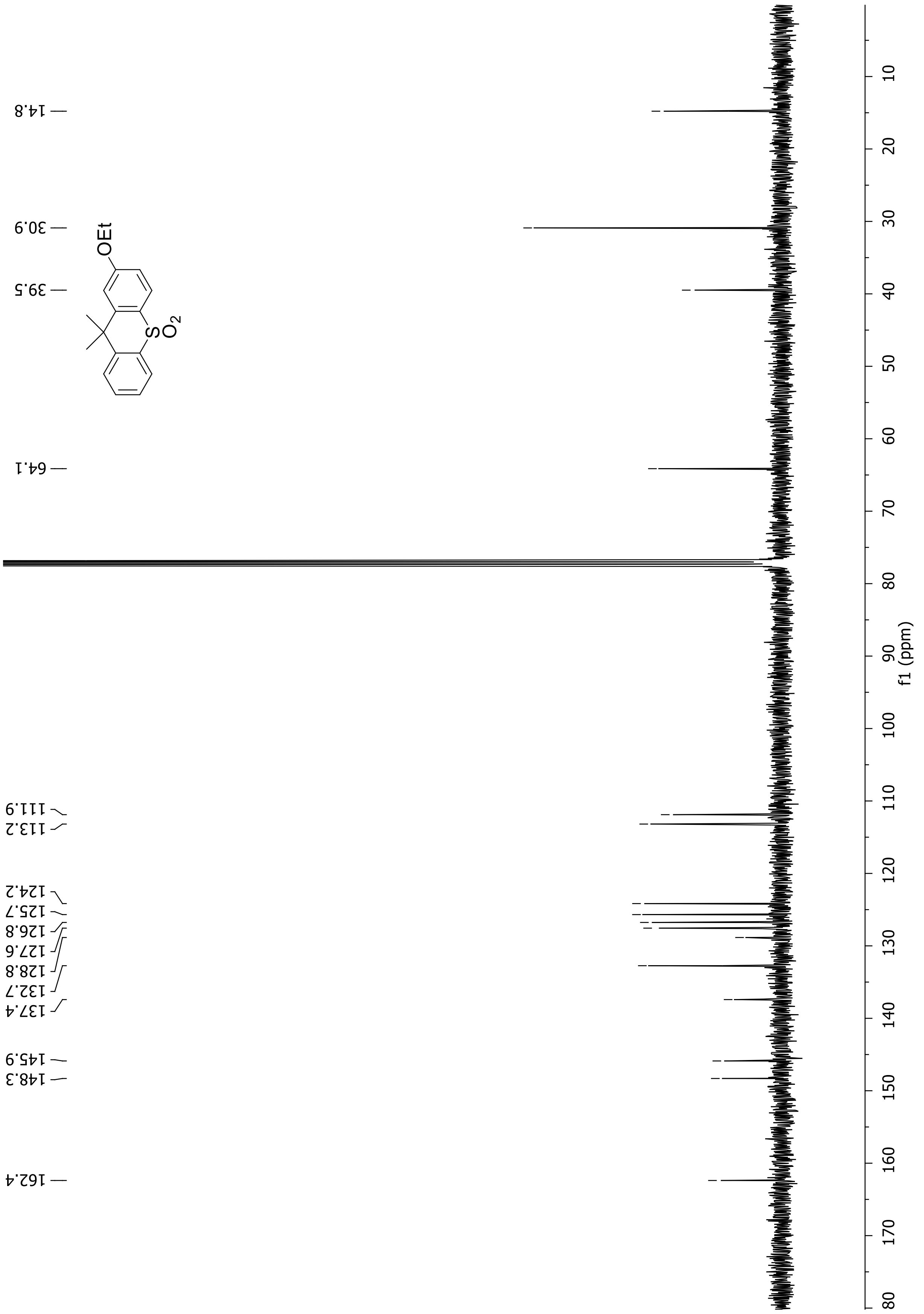
14.8
30.9
39.5
64.1
111.9
113.2
124.2
125.7
126.8
127.6
128.8
132.7
137.4
145.9
148.3
162.4
OEt
S
O2
f1 (ppm)
10
20
30
40
50
60
70
80
90
100
110
120
130
140
150
160
170
180

^{1}H NMR of **19**

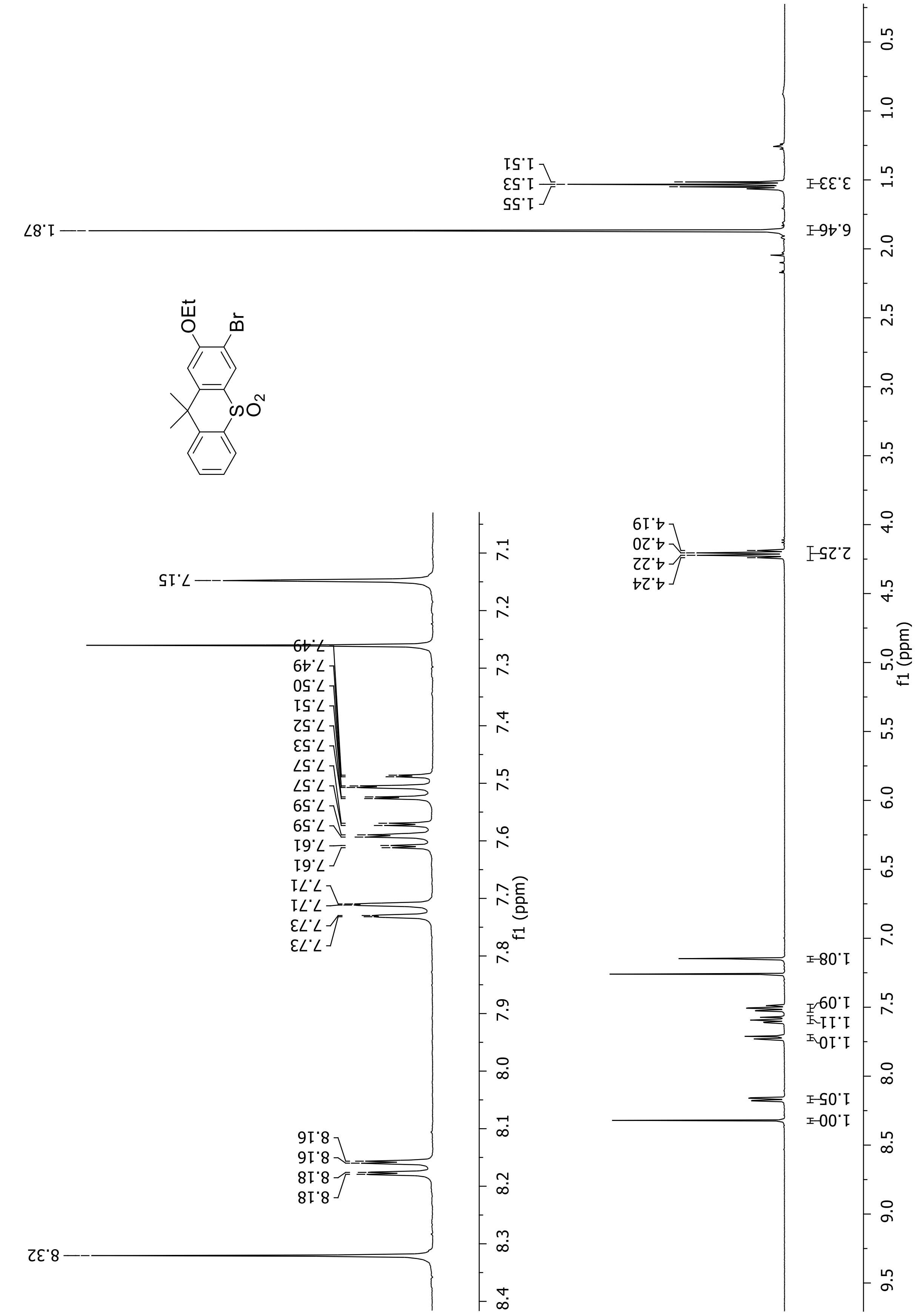

^{13}C NMR of **19**

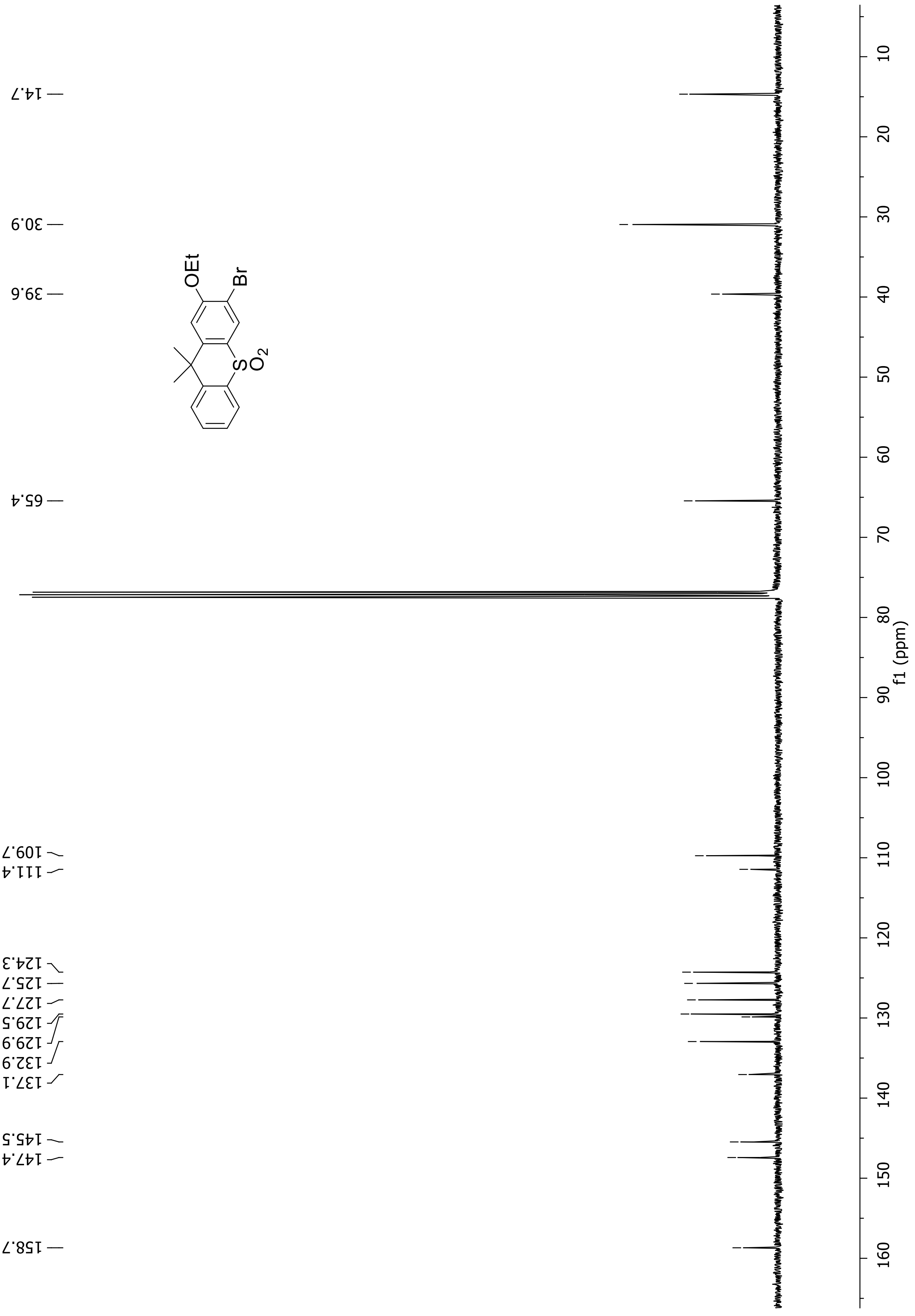
14.7
30.9
39.6
65.4
109.7
111.4
124.3
125.7
127.7
129.5
129.9
132.9
137.1
145.5
147.4
158.7
OEt
Br
S
O2
f1 (ppm)

^{1}H NMR of **20**

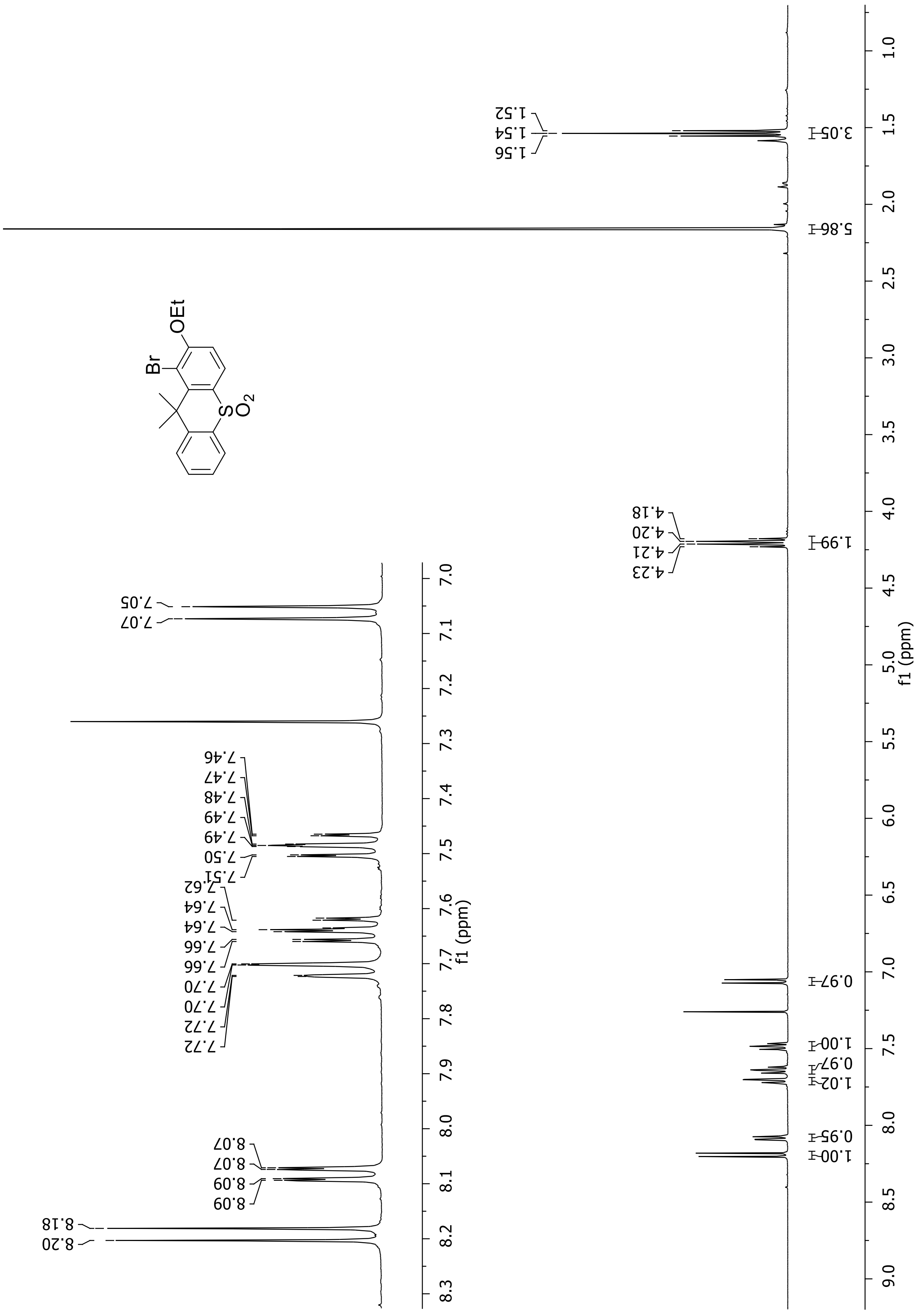

^{13}C NMR of **20**

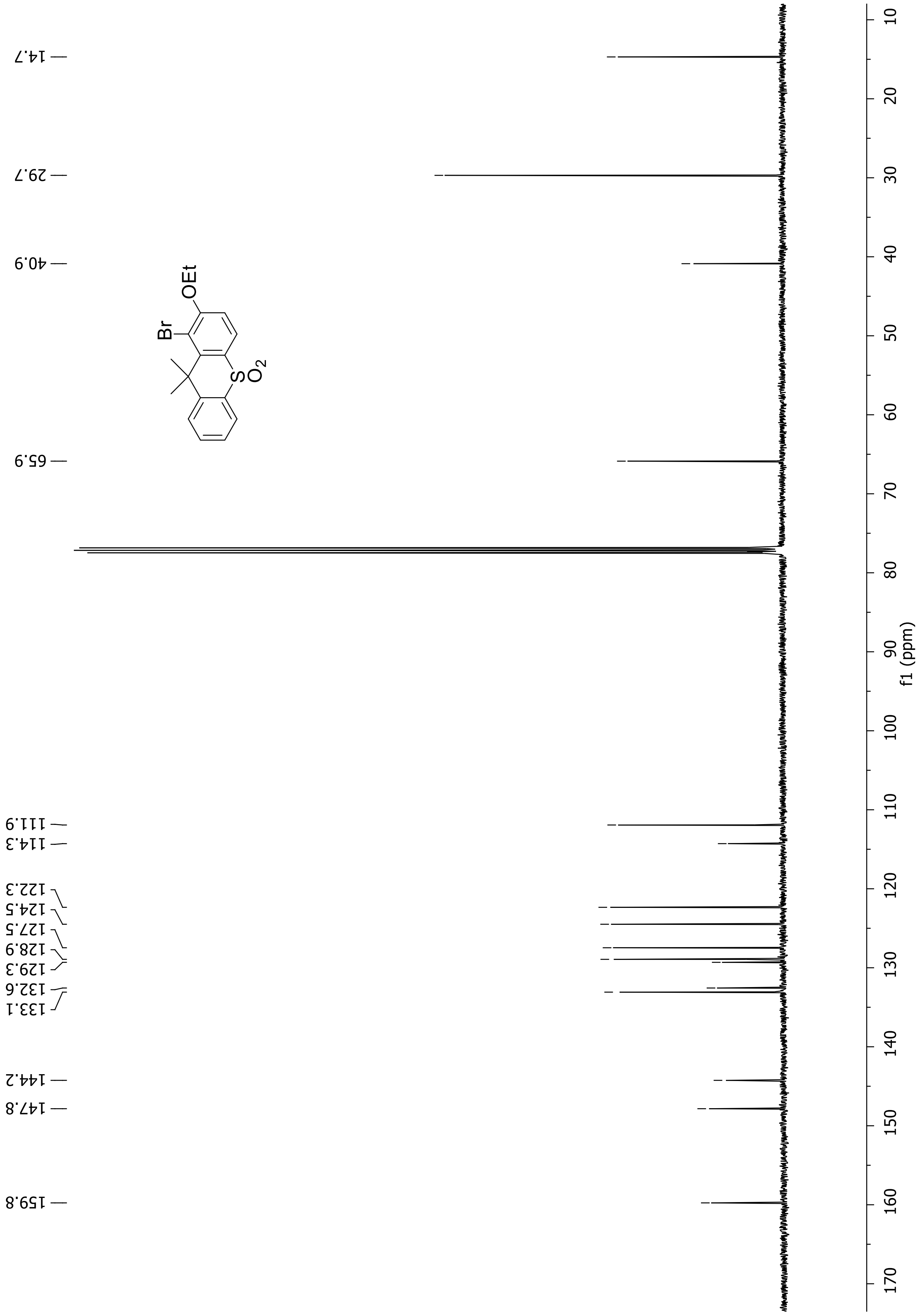
14.7
29.7
40.9
65.9
111.9
114.3
122.3
124.5
127.5
128.9
129.3
132.6
133.1
144.2
147.8
159.8
Br
OEt
S
O2
f1 (ppm)

^{1}H NMR of **33TXS**

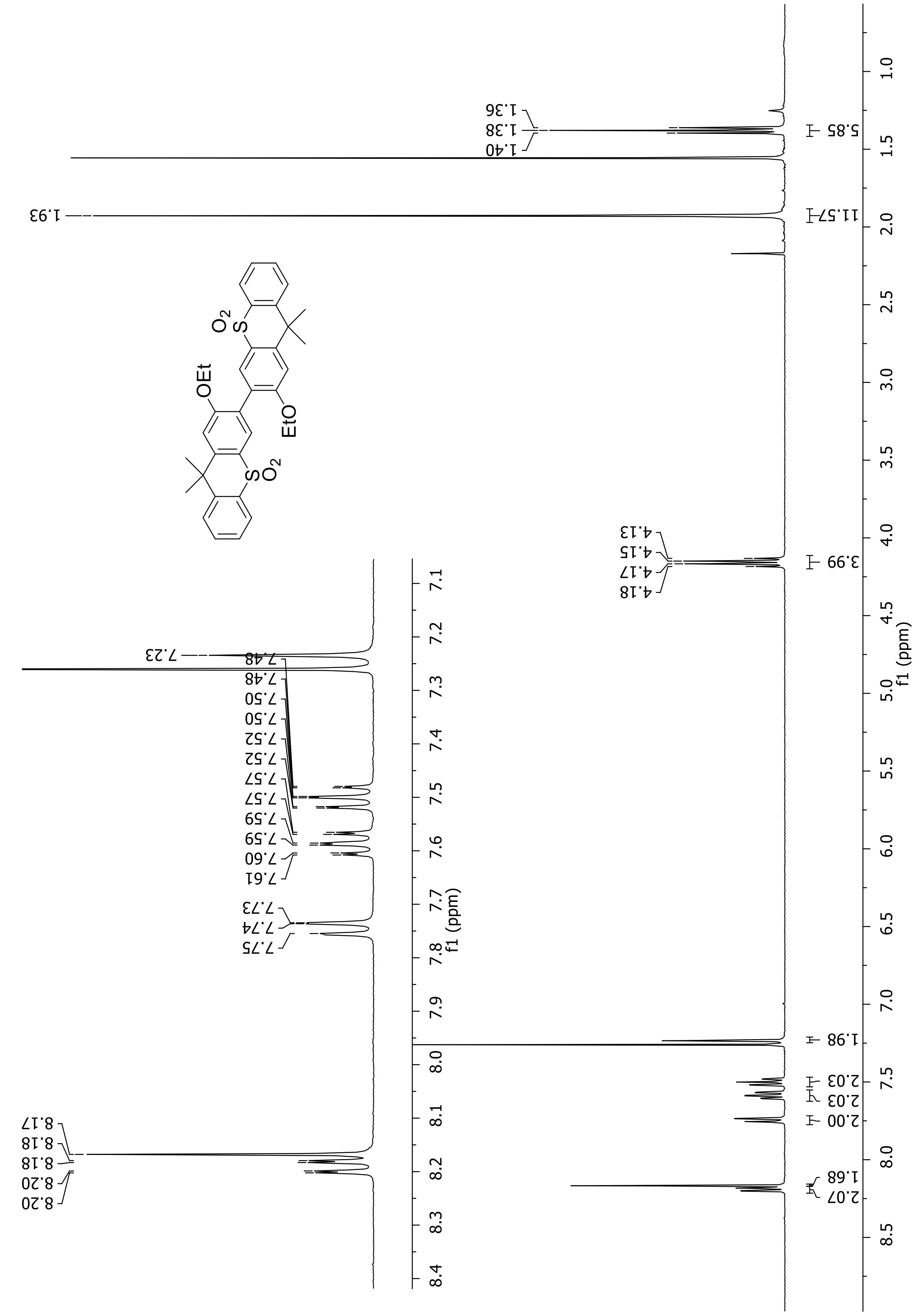

^{13}C NMR of **33TXS**

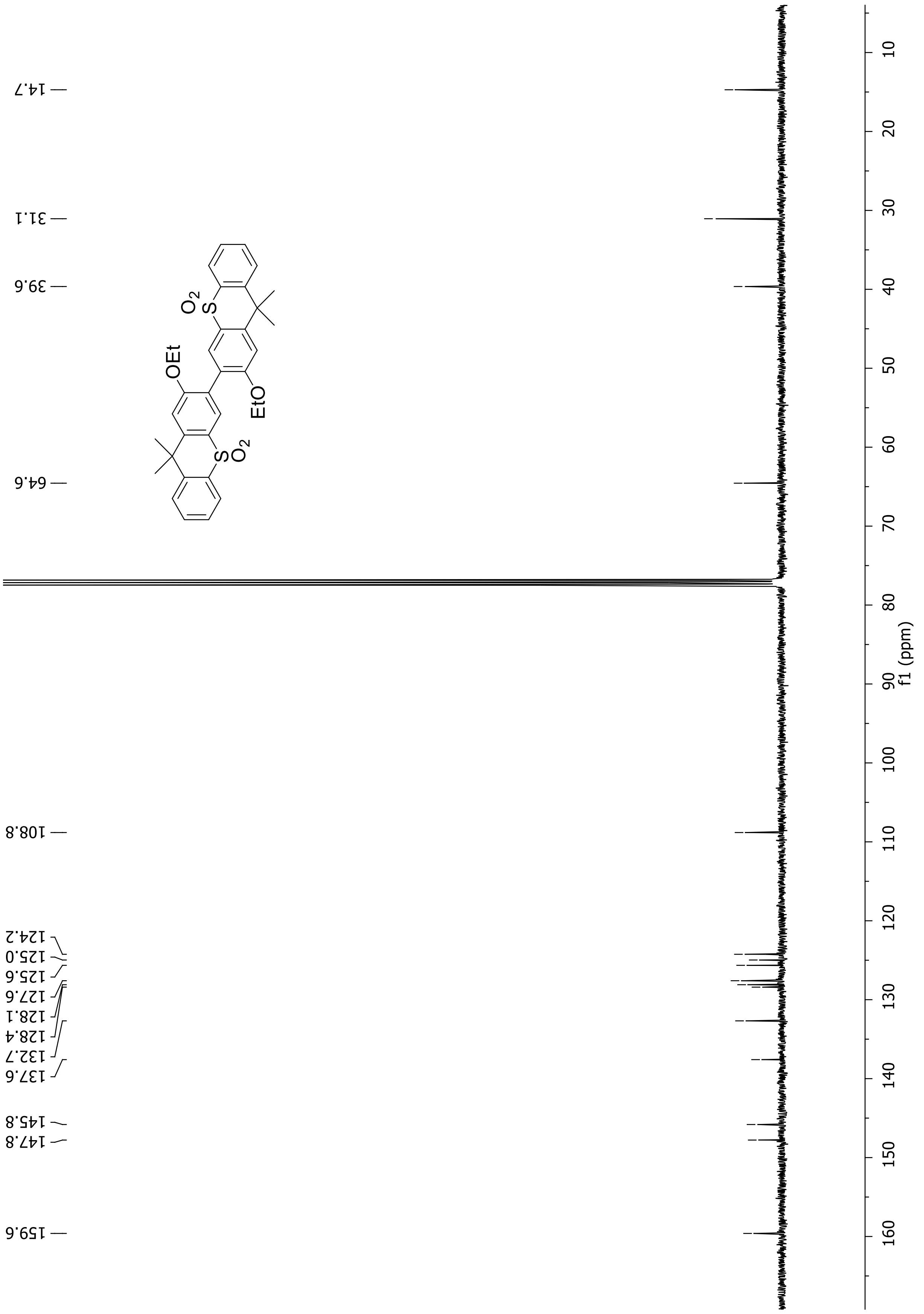

References

(1) Kauffman, J. M.; Litak, P. T.; Boyko, W. J. Syntheses and Photophysical Properties of Fluorescent Dibenzofurans, a Dibenzothiophene, and Carbazoles Substituted with Benzoxazole and Hydroxyl Groups to Produce Excited State Intramolecular Proton-transfer. *J. Heterocycl. Chem.* **1995**, *32* (5), 1541–1555. https://doi.org/10.1002/jhet.5570320523.

(2) Wrackmeyer, B. Carbon-13 NMR Spectroscopy of Boron Compounds. *Prog. Nucl. Magn. Reson. Spectrosc.* **1979**, *12* (4), 227–259. https://doi.org/10.1016/0079-6565(79)80003-5.

(3) Sheldrick, G. M. A Short History of SHELX. *Acta Crystallogr. Sect. A Found. Crystallogr.* **2008**, *64* (1), 112–122. https://doi.org/10.1107/S0108767307043930.

(4) Sheldrick, G. M. Crystal Structure Refinement with SHELXL. *Acta Crystallogr. Sect. C Struct. Chem.* **2015**, *71* (1), 3–8. https://doi.org/10.1107/S2053229614024218.

(5) Dolomanov, O. V.; Bourhis, L. J.; Gildea, R. J.; Howard, J. A. K.; Puschmann, H. OLEX2: A Complete Structure Solution, Refinement and Analysis Program. *J. Appl. Crystallogr.* **2009**, *42* (2), 339–341. https://doi.org/10.1107/S0021889808042726.

(6) Gavezzotti, A. Calculation of Lattice Energies of Organic Crystals: The PIXEL Integration Method in Comparison with More Traditional Methods. *Zeitschrift fur Krist.* **2005**, *220* (5–6), 499–510. https://doi.org/10.1524/zkri.220.5.499.65063.

(7) Turner, M. J.; McKinnon, J. J.; Wolff, S. K.; Grimwood, D. J.; Spackman, P. R.; Jayatilaka, D.; Spackman, M. A. *CrystalExplorer17*. University of Western Australia **2017**. http://hirshfeldsurface.net.

(8) Mackenzie, C. F.; Spackman, P. R.; Jayatilaka, D.; Spackman, M. A. CrystalExplorer Model Energies and Energy Frameworks: Extension to Metal Coordination Compounds, Organic Salts, Solvates and Open-Shell Systems. *IUCrJ* **2017**, *4* (5), 575–587. https://doi.org/10.1107/S205225251700848X.

(9) Neese, F. Software Update: The ORCA Program System, Version 4.0. *Wiley Interdiscip. Rev. Comput. Mol. Sci.* **2018**, *8* (1). https://doi.org/10.1002/wcms.1327.

(10) Neese, F. The ORCA Program System. *Wiley Interdiscip. Rev. Comput. Mol. Sci.* **2012**, *2* (1), 73–78. https://doi.org/10.1002/wcms.81.

(11) Hanwell, M. D.; Curtis, D. E.; Lonie, D. C.; Vandermeerschd, T.; Zurek, E.; Hutchison, G. R. Avogadro: An Advanced Semantic Chemical Editor, Visualization, and Analysis Platform. *J. Cheminform.* **2012**, *4* (8), 17. https://doi.org/10.1186/1758-2946-4-17.

(12) Hehre, W. J.; Ditchfield, K.; Pople, J. A. Self-Consistent Molecular Orbital Methods. XII. Further Extensions of Gaussian-Type Basis Sets for Use in Molecular Orbital Studies of Organic Molecules. *J. Chem. Phys.* **1972**, *56* (5), 2257–2261. https://doi.org/10.1063/1.1677527.

(13) Dill, J. D.; Pople, J. A. Self-Consistent Molecular Orbital Methods. XV. Extended Gaussian-Type Basis Sets for Lithium, Beryllium, and Boron. *J. Chem. Phys.* **1975**, *62* (7), 2921–2923. https://doi.org/10.1063/1.430801.

(14) Francl, M. M.; Pietro, W. J.; Hehre, W. J.; Binkley, J. S.; Gordon, M. S.; DeFrees, D. J.; Pople, J. A. Self-Consistent Molecular Orbital Methods. XXIII. A Polarization-Type Basis Set for Second-Row Elements. *J. Chem. Phys.* **1982**, *77* (7), 3654–3665. https://doi.org/10.1063/1.444267.
